# Smelting copper in decorated pottery: communities of practice in the Niari Basin, Republic of the Congo, fifteenth–seventeenth centuries CE

**DOI:** 10.1007/s12520-022-01653-9

**Published:** 2022-10-06

**Authors:** Braden W. Cordivari, Nicolas Nikis, Marcos Martinón-Torres

**Affiliations:** 1 Department of Archaeology, University of Cambridge, Cambridge, UK; 2 Present Address: Institute for the Study of the Ancient World, New York University, New York, NY USA; 3 Centre d’Anthropologie Culturelle, Université libre de Bruxelles, Brussels, Belgium; 4 Heritage Studies Unit, Royal Museum for Central Africa, Tervuren, Belgium

**Keywords:** Archaeometallurgy, African archaeology, Copper, Crucibles, Chaîne opératoire, Technology

## Abstract

This paper considers copper production in the Niari Basin, Republic of the Congo, during a period dated to the mid-fifteenth–mid-seventeenth centuries CE. Using a combination of pXRF, OM, SEM–EDS, and FTIR, it assesses the microstructure and composition of slags and technical ceramics from sites associated with two different regional pottery traditions: Moubiri-type at the site of Kingoyi near Mindouli and Kindangakanzi-type at Kindangakanzi near Boko-Songho. Both sites are characterised by the use of refractory domestic pottery as crucibles for copper smelting. Moubiri-type pottery is alumina-rich, while Kindangakanzi-type pottery is formed from a magnesia-rich clay, a crucible type unique in sub-Saharan Africa. Similarities in chaînes opératoires at Kingoyi and Kindangakanzi suggest sharing of knowledge at mining and smelting sites, interactions we reconstruct as a metallurgical constellation of practice comprised of the distinct potting communities of practice (see Supplementary information for abstract in Lingala and French).

**Supplementary Information:**

The online version contains supplementary material available at 10.1007/s12520-022-01653-9.

## Introduction

**Fig. 1:**
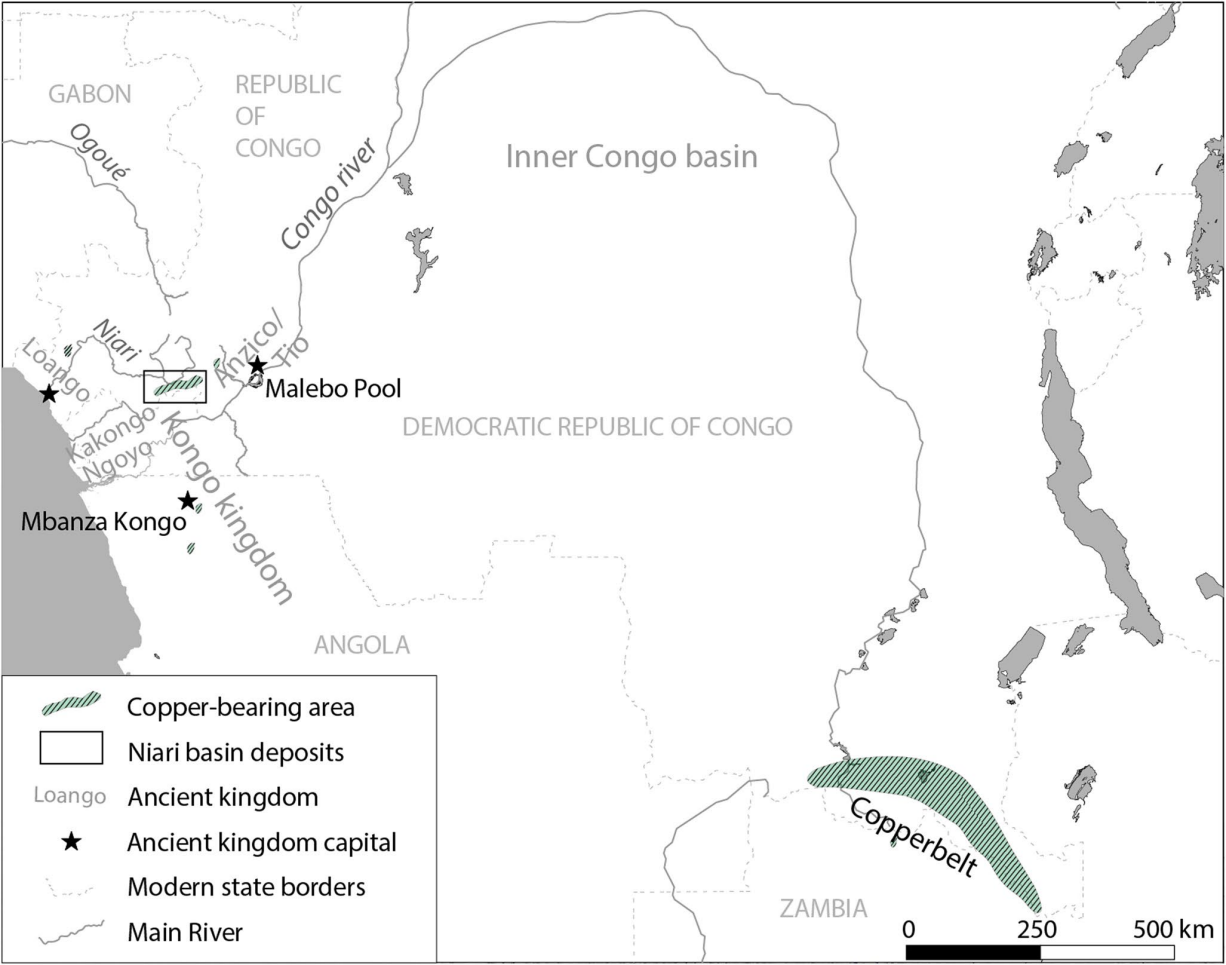
Niari Basin in relation to principal kingdoms of the fifteenth–seventeenth centuries and modern borders

This paper assesses copper production in the Niari Basin, southern Republic of the Congo, during a period dated to the fifteenth–seventeenth century CE (Fig. 1). Since its first exploitation in Central Africa in the mid-first millennium CE and for most of Central African history, copper, not gold, was the most highly valued metal, esteemed for its colour, sheen, and sonic properties (Herbert 1984). Copper was used for the creation of status items (Volavka 1998) and displays a close relationship with social/political cachet, evident from the early second millennium onward in the elite graves of the Upemba Depression (Connah 2016, pp. 299–329; de Maret 1999). Copper also played an important role in long distance trade as ingots (Bisson 1975; de Maret 1995; Nikis and Livingstone Smith 2017). In Central Africa in the course of the nineteenth century, similar value to unalloyed copper was also placed on imported European brass (Bisson 2000; Cline 1937; Dupré and Pinçon 1997; Herbert 1984; Nikis 2020).

The Niari Basin was the primary copper deposit historically exploited in West Central Africa, situated at a nexus of trade between the coast and the Inner Congo via the Malebo Pool. In the fifteenth–seventeenth centuries, it lay between the Kingdom of Kongo across the Congo River to the south (Clist et al. 2015, 2018; de Maret 2013; Hilton 1985; MacGaffey 2018; Ndinga-Mbo 1984; Thornton 2018; Thornton and Windmuller-Luna 2015), Anzico/Tio around the Malebo Pool (Vansina 1973), and the coastal polities, principally Loango (Denbow 2012, 2014; Martin 1972).

The Portuguese were active on the coast from the late fifteenth century onwards, particularly in their colony of Angola to the south and within the Kongo Kingdom, while Dutch influence grew quickly after their arrival in 1597, bolstered by trade with Loango north along the coast (Martin 1972). Copper, ivory, and redwood were traded with the Europeans there until the mid-seventeenth century, when the demand for slaves seemed to eclipse commerce, as it had in the sixteenth century in Portuguese Angola.

Prior to Belgian and French colonisation in the late nineteenth century, no Europeans visited the Niari mines (Dupont 1889; Pleigneur 1888). Europeans on the coast in the fifteenth–seventeenth century relied on generalised second-hand accounts, and it is not clear from these sources who controlled the mines: Kongo, Anzico, Loango, or locals (Dapper 1676; Hilton 1985, p. 55; Jones 1983; Ndinga-Mbo 1984). In the fifteenth–seventeenth centuries, the location of mines within the Kongo Kingdom was kept secret from the Portuguese, and the mines in the Niari may have been similarly deliberately safeguarded from European interest (Herbert 1984, pp. 140–141; Volavka 1998, pp. 196–210). The evidence for copperworking in the period, then, is archaeological data.

Archaeological research in the region began with excavations of a few copper production sites near Mindouli in the 1950s (Clist 2012) and 1970s–80s (Lanfranchi and Manima-Moubouha 1984; Manima-Moubouha 1987, 1988). The most recent research programme was conducted between 2013 and 2015, led by Nicolas Nikis, whose doctoral thesis presents, contextualises, and interprets the results of the project (Clist et al. 2014; Nikis et al. 2013; Nikis 2018a; Nikis and Champion 2014). Over 100 sites were surveyed around Mindouli, Boko-Songho, and Mfouati, 20 of which were excavated; collected material from 37 sites is catalogued by Nikis. Ores and artefacts from the Niari Basin were also analysed for lead isotope and chemical data (Rademakers et al. 2018).

**Fig. 2:**
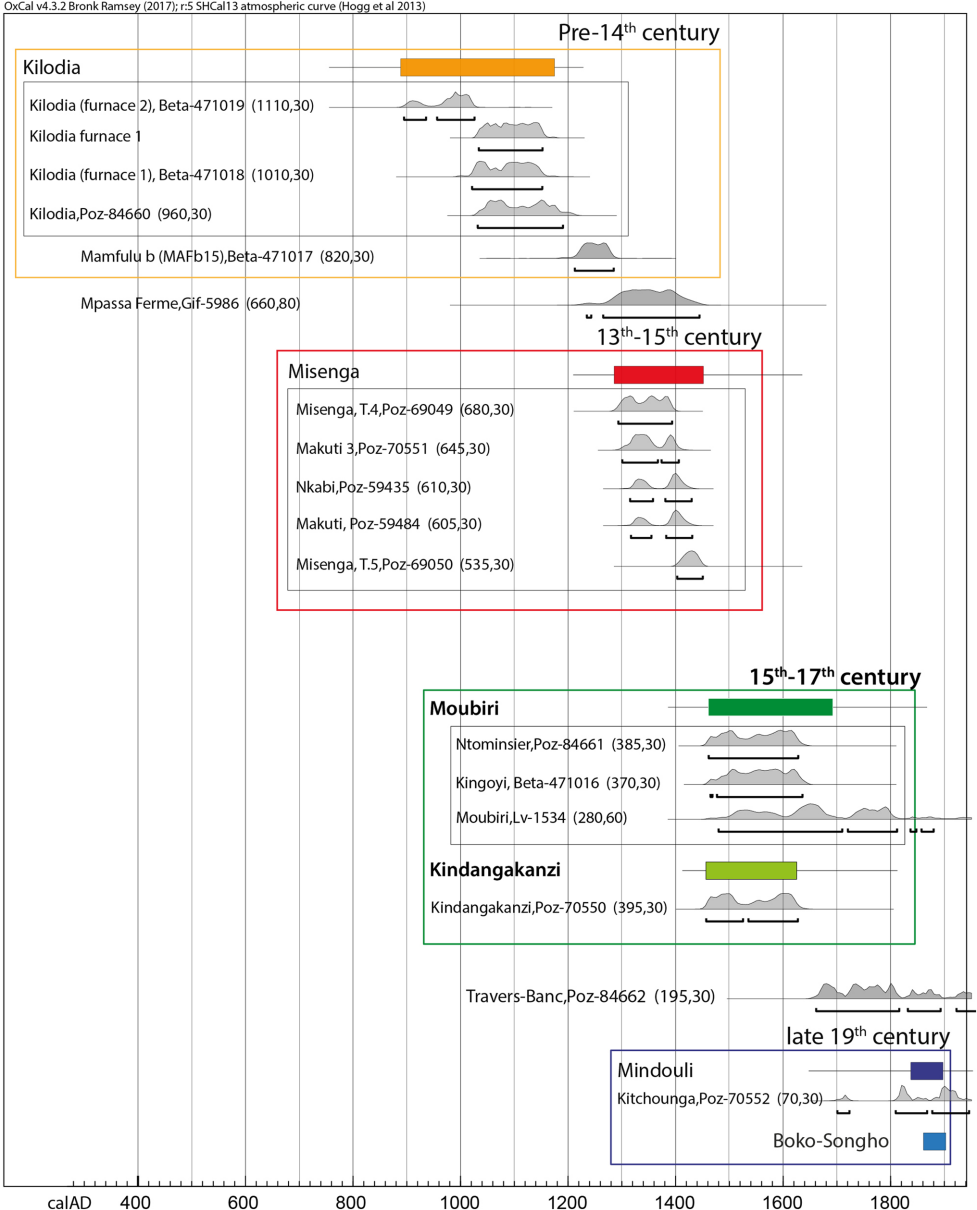
Chronological sequence for the Niari showing fifteenth–seventeenth century dates for production at Kingoyi and Kindangakanzi. Dates are 2 sigma ranges according to SHCal13 atmospheric curve (Hogg et al. 2013)

Nikis identified four major phases of copper production in the Niari from the tenth–nineteenth centuries CE, dated by radiocarbon and associated material culture (Fig. 2). This research demonstrated the diachronic importance of the Niari as a nexus of trade, which operated on varying scales and in different networks in each period (Nikis 2018a). By the fifteenth–seventeenth centuries, earlier local networks had likely expanded such that goods including ivory and copper flowed between the coast and the Malebo Pool via the Niari (Martin 1972). This route was increasingly used for the movement of enslaved peoples from the seventeenth into the early nineteenth century, when copper production slowed and possibly ceased altogether. In the late nineteenth century, following the cessation of the slave trade, local copper mining and smelting resumed and included the production of copper-lead alloys (Martin 1972; Nikis 2018a; Volavka 1998).

This paper concerns the third phase, dated to the fifteenth–seventeenth centuries CE. Unlike other periods, no finished metal objects from this period have been found, only traces of production. Copper production evidence from the fifteenth–seventeenth centuries, then, is a window into a key aspect of both the local social/economic dynamic and an articulation into larger regional and superregional narratives.

**Fig. 3:**
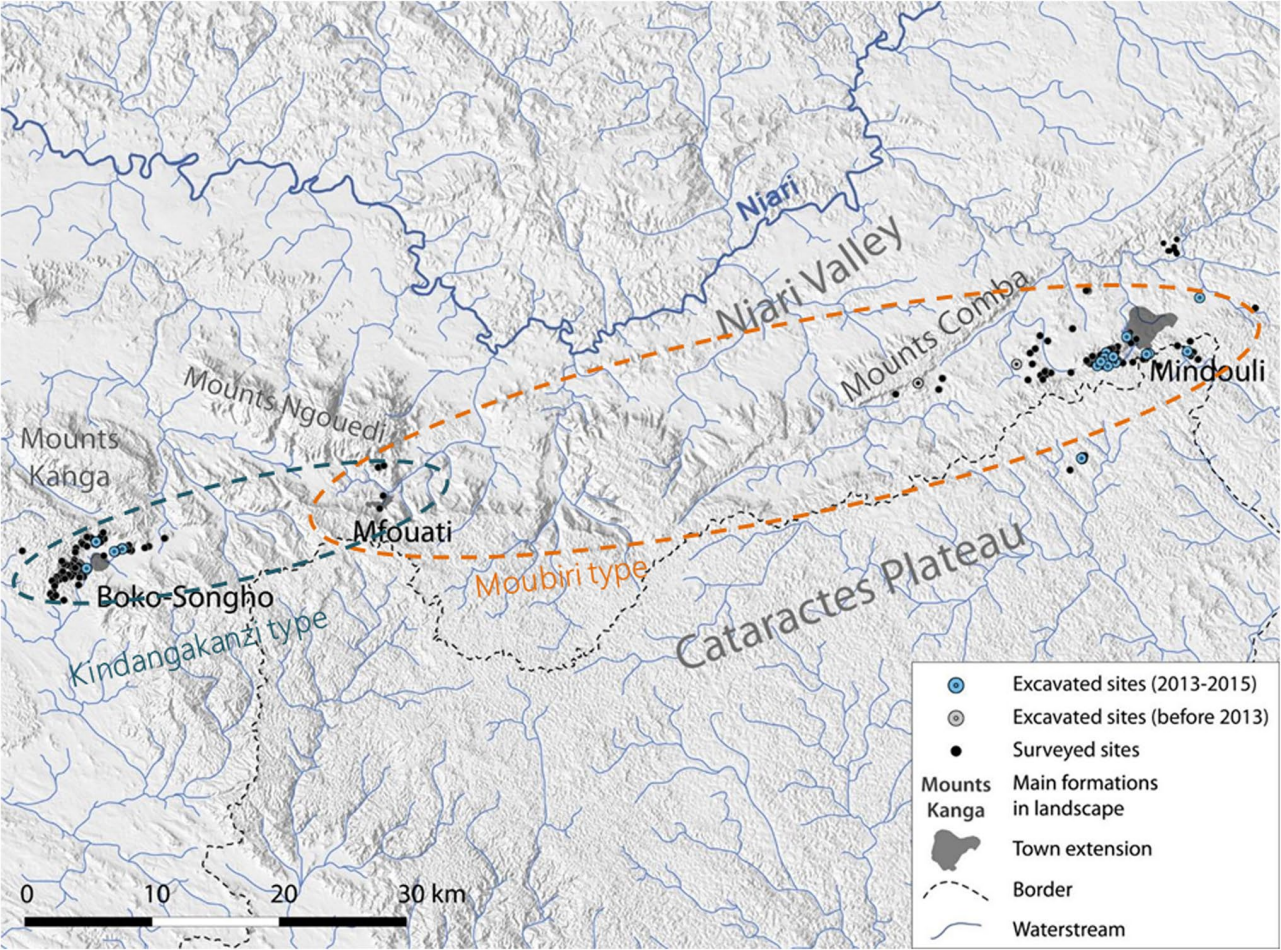
The Niari Basin region, showing sites around Boko-Songho, Mfouati, and Mindouli

**Fig. 4:**
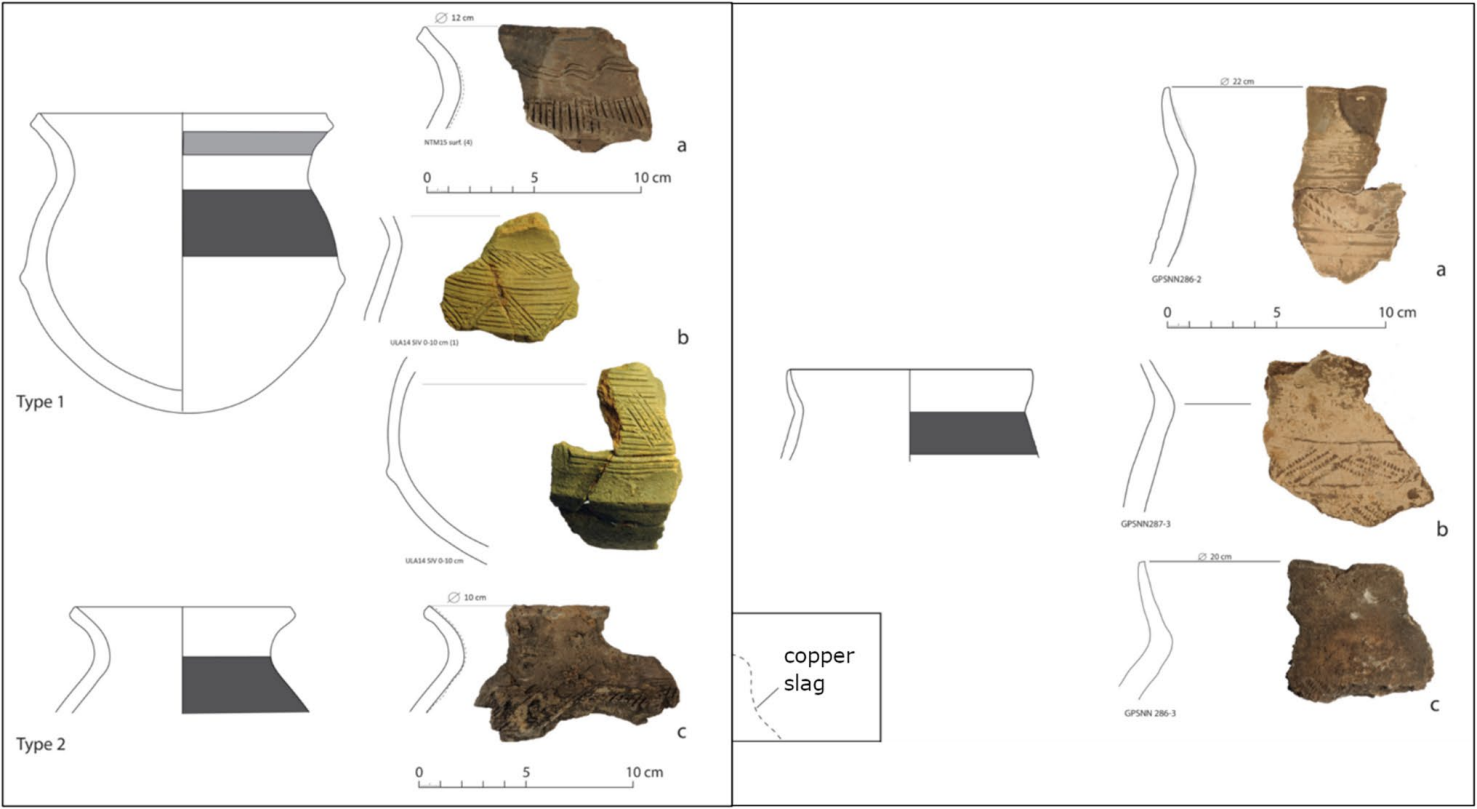
Moubiri-type pottery (left) and Kindangakanzi-type (right) (Nikis 2018a, Figs. 5.18, 5.21)

Miners in the Niari exploited mineralisation formed in faults between sandstone and limestone and within the limestone, in particular vein-filling malachite (Nikis and De Putter 2015, 2016; Rademakers et al. 2018, Supplementary information). Pottery found at copper production sites from this period can be differentiated typologically into two types: Moubiri- and Kindangakanzi-type. Moubiri-type pottery is principally found at sites around Mindouli to the east, and Kindangakanzi-type around Boko-Songho to the west (Figs. 3 and 4). Both pottery types are found together around Mfouati between these two zones. This copresence helps establish the contemporaneity of the two pottery types and suggests potential for inter-group interaction. Whether these groups resided in the Niari at unidentified settlement sites or were in the valley temporarily to exploit copper is unknown (Nikis 2018a, pp. 299–318). Sites across the Niari in this period are located close to mines and show no signs of permanent habitation, and neither pottery type has previously been associated with any known regional production.

This paper characterises copper production around Mindouli, associated with Moubiri-type pottery, and around Boko-Songho, associated with Kindangakanzi-type pottery. It seeks to understand whether and how miners, smelters, and potters in the two regions related to each other technologically and socially. We examine material here from one site within each region: Kindangakanzi near Boko-Songho and Kingoyi near Mindouli. Both sites were surveyed and excavated by Nikis in 2014–2015 (see Supplementary information). This study is the first investigation of Central African technical ceramics using laboratory techniques.

We consider similarities and differences in chaînes opératoires, assessing choices in materials and actions at each step as potential evidence for knowledge sharing (Coupaye 2015; Kuijpers 2018; Martinón-Torres 2002). A significant element is the common practice of reusing decorated domestic pottery as crucibles, Moubiri-type around Mindouli and Kindangakanzi-type around Boko-Songho. This shared practice stands in contrast to both the preceding and following periods in the Niari, when the people mining and smelting employed specialised crucibles (Nikis 2018a, pp. 251–298, 339–378).

## Materials and methods

**Fig. 5:**
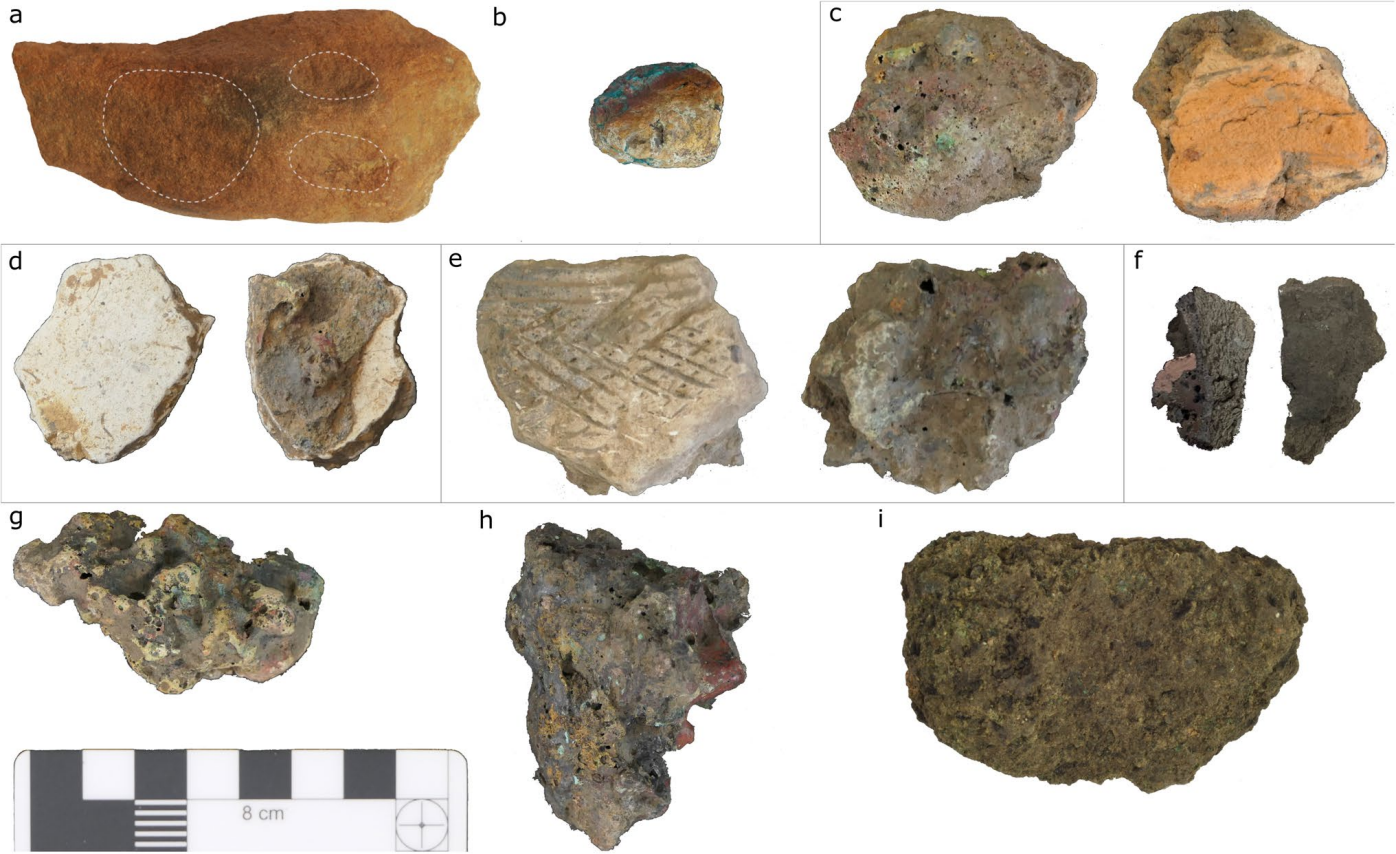
Metallurgical debris from Kingoyi. **a** Fragment of sandstone possibly used as anvil. **b** Ore MKU3b14_7. **c** Tuyère MKU3b15_2. **d** Crucible MKU3b14_3 (Kindangakanzi-type). **e** Crucible MKU3b15_5 (Moubiri-type). **f** Crucible MKU3b15_13 (Moubiri-type). **g** Slag lump MKU3b14_8. **h** Slag lump MKU3b14_5. **i** Soil aggregate MKU3b15_26

**Fig. 6:**
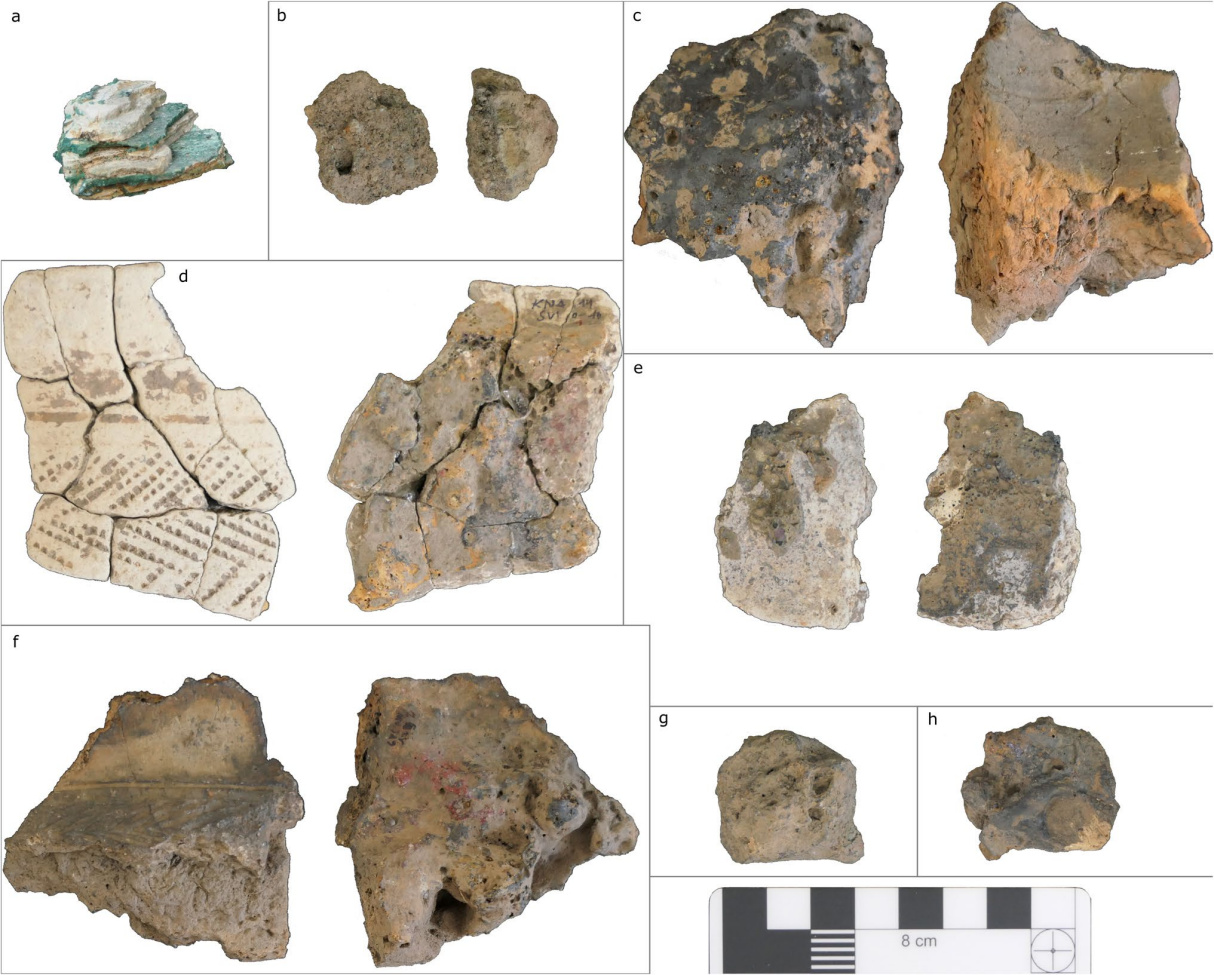
Metallurgical debris from Kindangakanzi. **a** Malachite ore GPSNN272_5 (from Malembe near Kindangakanzi). **b** Furnace wall fragment KNA14_37. **c** Tuyère KNA14_10. **d** Crucible KNA14_9. **e** Crucible KNA14_29 slagged on both sides. **f** Crucible KNA14_11. **g** Slag lump KNA14_25. **h** Slag lump KNA14_26

All analyses were undertaken at the archaeological science laboratories of the Department of Archaeology, University of Cambridge. Of the roughly 10.5 kg of metallurgical debris from the two sites, 85 samples of slags, ores, crucibles, tuyères, furnace wall, and domestic pottery were selected for analysis (Table 1). These were initially screened via macroscopic observation and pXRF (see Supplementary information for analytical details and data quality assessment). A subset of samples (*n* = 17) was then prepared as polished blocks for optical microscopy and SEM–EDS (Figs. 5 and 6). These were chosen in batches according to an adaptive sampling strategy, seeking to cover all the variability documented by pXRF and ongoing microstructural analyses.

**Table 1:** Samples prepared as polished blocks for OM/SEM–EDS (*n* = 17)

Sample	Area	Site	Type
KNA14_9	Boko-Songho	Kindangakanzi	Crucible
KNA14_11	Boko-Songho	Kindangakanzi	Crucible
KNA14_29	Boko-Songho	Kindangakanzi	Crucible
KNA14_37	Boko-Songho	Kindangakanzi	Furnace wall
KNA14_8	Boko-Songho	Kindangakanzi	Pottery
KNA14_25	Boko-Songho	Kindangakanzi	Slag
KNA14_26	Boko-Songho	Kindangakanzi	Slag
KNA14_10	Boko-Songho	Kindangakanzi	Tuyère
MKU3b15_5	Mindouli	Kingoyi	Crucible
MKU3b15_13	Mindouli	Kingoyi	Crucible
MKU3b14_3	Mindouli	Kingoyi	Crucible
MKU3b14_9	Mindouli	Kingoyi	Ore
MKU3b15_3	Mindouli	Kingoyi	Pottery
MKU3b15_4	Mindouli	Kingoyi	Pottery
MKU3b14_5	Mindouli	Kingoyi	Slag
MKU3b14_8	Mindouli	Kingoyi	Slag
MKU3b15_2	Mindouli	Kingoyi	Tuyère

Samples were cut and mounted in epoxy resin, cured overnight, and then ground and polished to 1 μm. Polished blocks were first examined on a Keyence VHX-6000 digital microscope in both plane- and cross-polarised light for assessing macro-scale sizes and distributions of inclusions and for locating and contextualising areas for SEM analysis. Composite images were stitched at 200 × in plane-polarised light.

Samples were then analysed by SEM on a Hitachi TM3000 SEM with Oxford Instruments x-stream-2 EDS system and AztecOne software. Accelerating voltage was 15 kV and working distance 9.5 mm. The EDS was referenced to USGS basalt standard BIR-1G to assess data quality, which showed excellent precision and accuracy for all elements in concentrations above quantification limits (estimated around 0.1–0.3%) (Supplementary information).

In addition to spot analyses for slag phase identification and qualitative identification of impurities in metal prills, bulk chemical composition was calculated by averaging multiple readings at 500 × for typical ceramic and slag areas, avoiding large pores/inclusions. Spectra were assessed visually, quantified as stoichiometric oxides where appropriate, and normalised to 100% by weight. All percentages given in text and tables refer to weight percent (wt%).

While SEM–EDS quantification is limited by the porous nature of ceramics/slags, and by the presentation of the data in the form of a single oxide, its ability to gather contextually specific compositions, a combination of microstructural and chemical data, is key to reconstructing reaction parameters. In this study, spot-specific analysis of small grains was hampered by the electron beam collecting data from the surrounding surface volume. This did not affect bulk chemistry.

Samples were also analysed via FTIR (*n* = 13) for compound identification of ores and ceramic inclusions and to investigate the presence of high-temperature silica polymorphs in slags. Analysis was undertaken via the KBr method on a Thermo-Nicolet iS5 with iD1 transmission, with spectra collected between 4000 and 400 cm^−1^. Compounds were identified with reference to the Kimmel Center for Archaeological Science Infrared Standards Library, Weizmann Institute of Science (Weiner 2010) and RRUFF database (Lafuente et al. 2015).

## Results

### Ores

Three samples of ore were analysed (see Supplementary information). Two samples from Kingoyi were identified as goethite-dioptase and dioptase, and one sample from Malembe around Boko-Songho as malachite intergrown with talc-calcite. Given the small sample size, these results are broadly indicative of the available ore and gangue and are consistent with the exploitation of secondary carbonates/silicates, identifiable to ancient prospectors by their green colour. The occurrence of small fragments of unburnt goethite- and dioptase-rich ores on-site at Kingoyi may suggest a process of crushing/sorting and the rejection of non-malachite ores.

### Technical ceramics

**Fig. 7:**
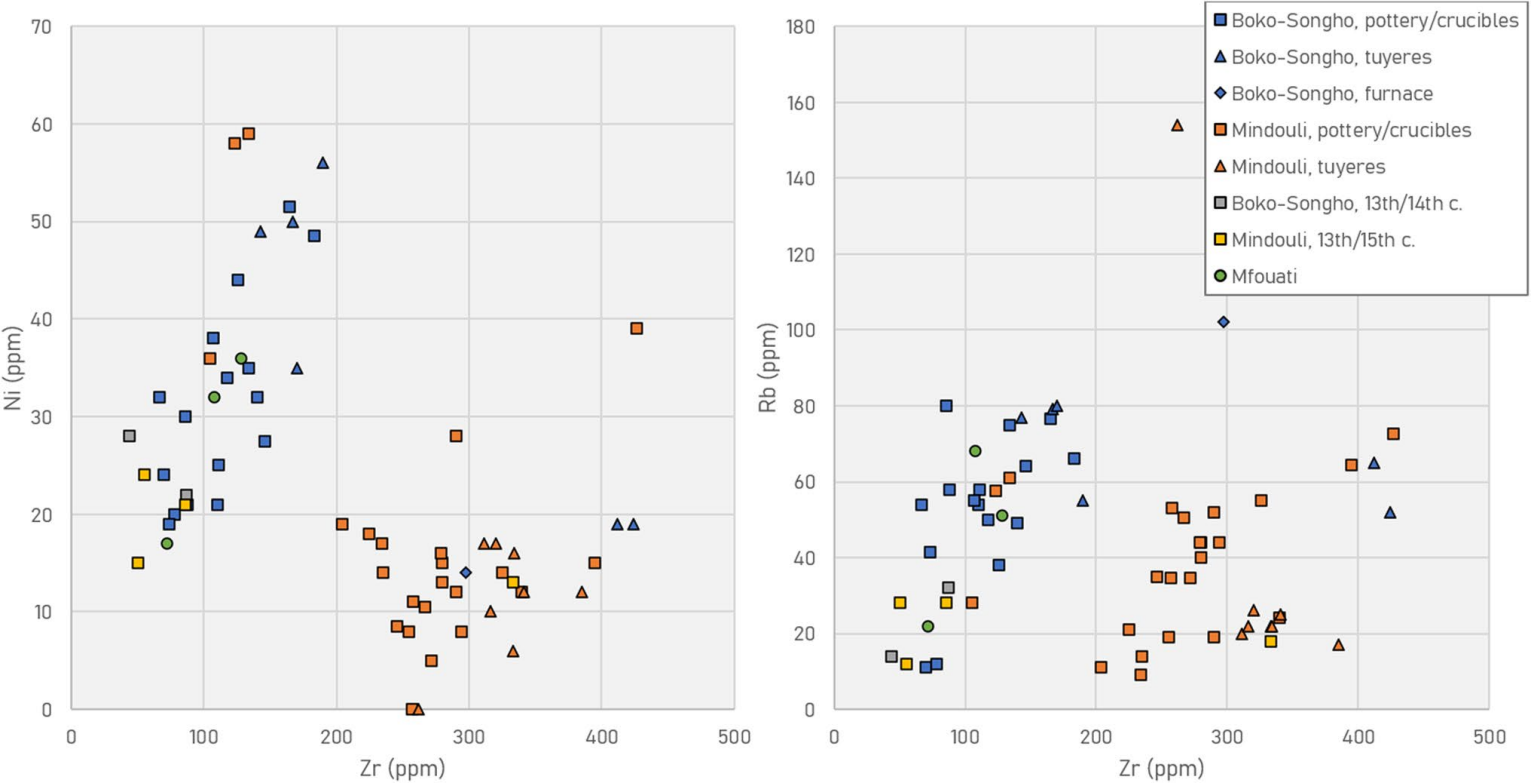
pXRF plots of Zr vs Ni (L) and vs Rb (R). Objects from Mfouati group with those from Boko-Songho. Kindangakanzi-type vessels found at Kingoyi group with the same type found at Kindangakanzi, while some technical ceramics from the site of Kindangakanzi (furnace, tuyères) are consistent with the Moubiri-type pottery around Mindouli. Note also the general grouping of fifteenth–seventeenth century pottery/crucibles with locally made tuyères of the same period and of pottery/crucibles from earlier periods, suggesting production within the Niari

Technical ceramics in the Niari include domestic pottery reused as crucibles and purpose-made tuyères and furnaces. Trace element patterns show a relatively cohesive group from around Mindouli, including Moubiri-type pottery from Kingoyi, and a second group from around Boko-Songho, including Kindangakanzi and its pottery style (Fig. 7). It is not presently possible to discriminate subgroups, and full study of pottery production in the Niari is beyond the present scope.

**Fig. 8:**
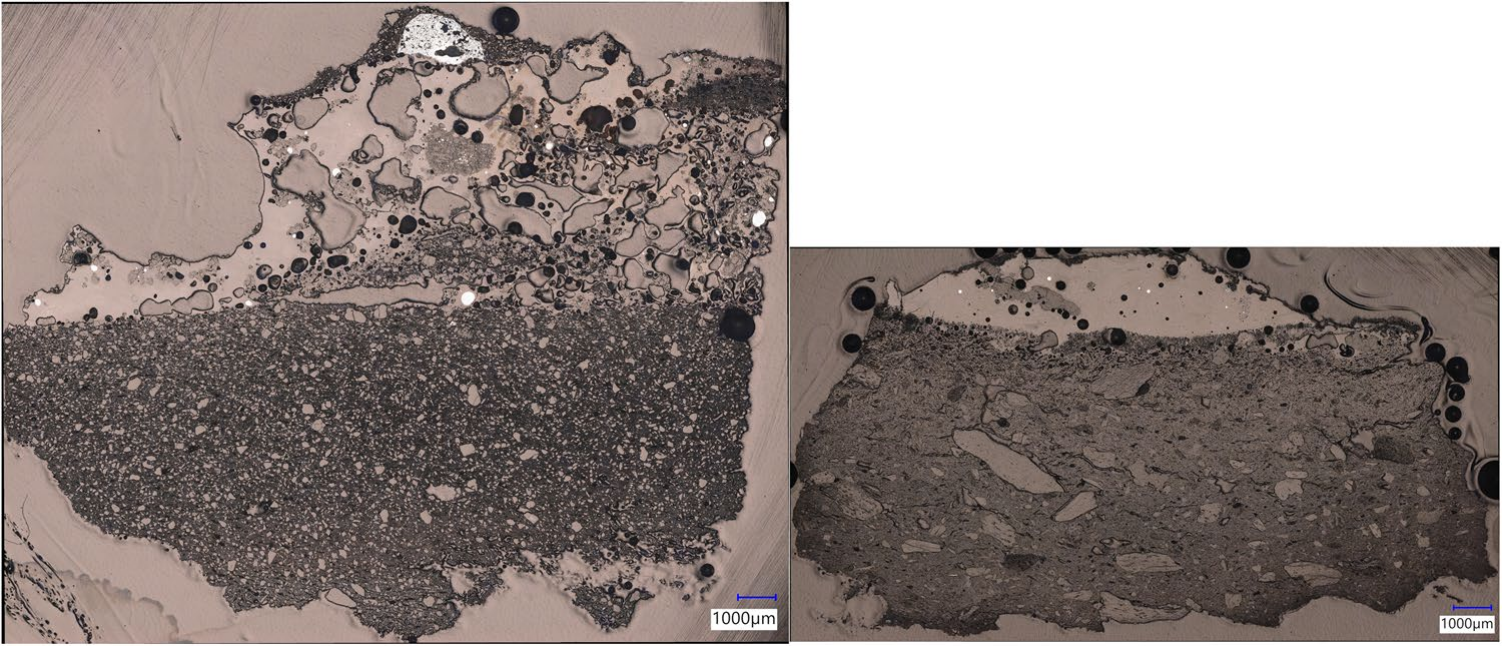
Cross-section of typical fabric of a quartz-based Moubiri-type crucible (MKU3b15_5) (L) and a talc-based Kindangakanzi-type crucible (KNA14_9) (R)

These elemental groups correspond to different fabric types: quartz-rich for Moubiri-type pottery and talc-rich for Kindangakanzi-type (Fig. 8), chemically differentiated by the percentage of alumina (Al_2_O_3_) versus magnesia (MgO). FTIR indicates that Moubiri-type pottery is consistent with being kaolinitic, while Kindangakanzi-type pottery is characterised by saponite, a variety of smectite, although exact identification of clay minerals solely by FTIR is difficult and multiple clay minerals are often present (Table 2, see also Supplementary information).

**Table 2:** Pottery types, regions, studied sites, and key characteristics in the present study

	Kindangakanzi-type pottery	Moubiri-type pottery
Region	Boko-Songho	Mindouli
Site	Kindangakanzi	Kingoyi
Clay mineral type	Smectite	Kaolinite
Principal inclusion	Talc/saponite	Quartz
Refractoriness	Magnesia-rich	Alumina-rich

**Fig. 9:**
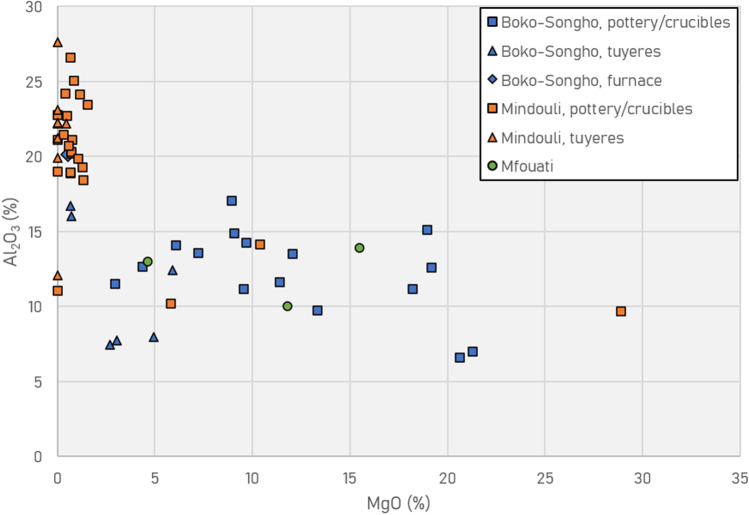
pXRF plot of MgO vs Al_2_O_3_ showing grouping of high-alumina Moubiri-type pottery/crucibles found around Mindouli, including at the site of Kingoyi, and high-magnesia Kindangakanzi-type pottery found around Boko-Songho, including at Kindangakanzi. Note that the high-magnesia vessels from Kingoyi are Kindangakanzi-type pottery. Note also that tuyères around Boko-Songho are made from both fabric types and that the furnace at Kindangakanzi in the region of Boko-Songho is made of a high-alumina clay

Both fabrics are highly refractory and display a remarkable lack of bloating, with a relatively sharp interface between ceramic and slag layers. Typical refractoriness plots of alumina vs alkali to assess ceramic refractoriness (Martinón-Torres and Rehren 2014) misrepresent the Niari material, as the high magnesia content of Kindangakanzi-type pottery overcomes the offset of the other alkalis. In fact, the quantity of magnesia in Kindangakanzi crucibles renders them truly refractory. It is more appropriate here to plot Al_2_O_3_ vs MgO. The SEM–EDS data, discussed below, demonstrate the high values of alumina and magnesia for Moubiri-type and Kindangakanzi-type pottery, respectively, meaning that the relative qualitative patterns identified in the broader pXRF screening can be associated with these different types of refractoriness (Fig. 9).

#### Tuyères/furnace

Tuyères from both sites were formed around reeds, with organic impressions visible on the interior surfaces. They were not fired prior to use, becoming transformed into ceramic by the heat of the metallurgical reaction, and are generally heavily slagged. Tuyères from Kindangakanzi are slightly larger and more conical than those from Kingoyi.

The internal diameters of the tuyères are ca. 3.5 cm around Mindouli, including Kingoyi, and 4–5 cm from sites around Boko-Songho, including Kindangakanzi (Nikis 2018a, pp. 308, 323). These are typical diameters for tuyères operated with bellows rather than blowpipes, for which the opening would be narrower (ca. 0.5–1.0 cm), or natural draught, for which it would be wider (> 5 cm) (Rehder 1994). Wooden drum bellows used in copper production were reported in the area in the nineteenth century (Laman 1953, p. 124; Pleigneur 1888, p. 279).

Tuyère MKU3b15_2 is made of the refractory quartz-based fabric and displays little bloating and high structural integrity. The other Mindouli tuyères examined via pXRF cluster together, with slightly more Zr than Moubiri-type pottery, while MKU3b15_2 lies outside both groups (Fig. 7). This suggests a relatively discrete local clay source exploited for tuyères, related but separate from those used for making Moubiri-type pottery, and a second source for tuyère MKU3b15_2.

The fragments of furnace wall (KNA14_37) and the examined tuyère from Kindangakanzi (KNA14_10) are made of high-alumina, low-alkali quartz-rich clay, similar to the Moubiri-type pottery/crucibles in use around Mindouli in terms of material properties (Table 3). They are, however, from separate sources as distinguished by their trace elements (Fig. 7). There are also talc-based tuyères at Kindangakanzi with similar trace element signatures and refractory properties to talc-based Kindangakanzi-type pottery. The creation of purpose-made technical ceramics in both fabrics at Kindangakanzi indicates that both quartz- and talc-based clays were available in close proximity to the site.

**Table 3:** Bulk chemical composition of ceramics (SEM–EDS), averaged from at least three readings per sample. All data are normalised to 100 wt% with oxygen added via stoichiometry. Blank cells denote values below detection limits

Site	Sample	Type	Na_2_O	MgO	Al_2_O_3_	SiO_2_	P_2_O_5_	K_2_O	CaO	TiO_2_	MnO	FeO	CuO	PbO
Kindangakanzi	KNA14_11	Crucible		22.9	11.5	58.1		1.2	0.9	0.5		4.9		
Kindangakanzi	KNA14_29	Crucible	0.2	20.7	10.4	63.5		1.3	0.4	0.3		3.1		
Kindangakanzi	KNA14_9	Crucible	0.2	13.7	11.9	64.4	1.1	1.6	1.6	0.5		5.0		
Kindangakanzi	KNA14_37	Furnace		0.9	22.7	60.3	0.5	2.1	0.2	0.9		7.2	2.8	2.7
Kindangakanzi	KNA14_8	Pottery		15.1	18.2	57.6		1.7	0.8	0.5		6.2		
Kindangakanzi	KNA14_10	Tuyère		0.9	21.6	66.4		1.9	0.1	1.0		7.9	0.5	
Kingoyi	MKU3b14_3	Crucible	0.2	21.5	10.6	59.5	0.7	1.9	1.4	0.3		3.8		
Kingoyi	MKU3b15_13	Crucible		0.9	29.4	62.9		0.2	0.2	0.5		5.8		
Kingoyi	MKU3b15_5	Crucible		1.6	19.5	71.7		1.0	0.1	0.5		5.6		
Kingoyi	MKU3b15_3	Pottery		1.5	18.8	72.9		0.9	0.2	0.4		5.2		
Kingoyi	MKU3b15_4	Pottery		12.8	14.5	63.1	0.3	3.0	0.6	0.4	0.1	5.3		
Kingoyi	MKU3b15_2	Tuyère		0.3	16.1	76.8		0.4	0.1	0.9		5.3		

#### Crucible fabrics

There are two subtypes of Moubiri pottery known: both are varieties of a closed hemispherical shape, with a rim diameter ca. 11–16 cm and decoration primarily around the shoulder, usually in the form of incised lines and raised bands of false carination (Fig. 4). Typical thicknesses are between 6 and 8 mm.

The fabric contains angular to subrounded quartz throughout the coarse fraction (largest size < 0.6 mm), continuing into the fine fraction (defined here as < 0.125 mm), with occasional Fe- or Ti–rich nodules (< 20 μm). A typical fabric microphotograph is shown in Fig. 8 and bulk chemical composition gathered via SEM–EDS is shown in Table 3.

Naturally quartz-rich clays, derived from nearby Mpioka Subgroup quartzites/siltstones, may have been exploited without the need for tempering (Cailteux et al. 2015). The similarity in matrix and bulk chemical compositions indicates that the clay composition is either natural or tempered with aplastic inclusions from the same geological formation (Supplementary information). While quartz increases refractoriness and is a common temper in pre-industrial crucibles (Kilikoglou et al. 1998; Martinón-Torres and Rehren 2014; Tite et al. 2001), these vessels are reused domestic pottery, meaning that the choice of quartz-rich fabrics, whether natural or artificially tempered, would have been made in regard to them as pottery, not as crucibles.

Kindangakanzi-type pottery is known in closed shapes, generally ellipsoidal with a flared neck, and is characterised by combed decoration on the shoulder and by a paler, almost blue, surface colour. The vessels are larger in size than Moubiri-type pottery, with a maximum rim diameter of 22 cm. Thicknesses are between 6 and 12 mm.

The fabric contains angular to rounded talc/saponite inclusions (< 4.9 mm), which contribute to high bulk magnesia values, up to 22.9%. Kindangakanzi-type pottery is a truly refractory ceramic which performed extremely well under high temperatures, with only limited bloating at the interior, and a sharp ceramic/slag interface.

Talc occurs in the Niari in two distinct types of deposits (Bigotte 1955; Cailteux et al. 2015; Guenot 1970; Noack et al. 1989): as oolites in a silica matrix in layers a few cm thick within dolomite of the Bangu Formation (‘Kisantu oolite’ of the Schisto-Calcaire), and within veins of hydrothermal alteration associated with copper mineralisation.

Kindangakanzi-type ceramics are non-calcareous (< 1.6% bulk CaO, < 1.5% matrix CaO) and lack dolomitic calcite, indicating the clays do not derive from Bangu dolomite. The similarity in matrix and bulk composition (Supplementary information) indicates that the clay was naturally talc-rich.

**Fig. 10:**
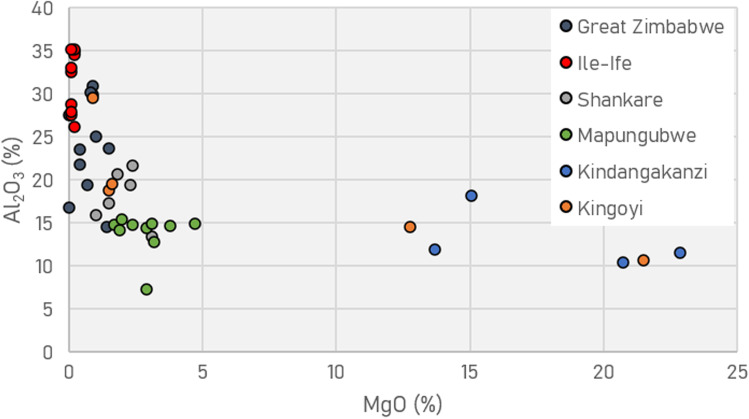
Comparison of crucibles from Kindangakanzi and Kingoyi (SEM–EDS data) with other sub-Saharan examples (Babalola et al. 2018; Bandama et al. 2017; Chirikure et al. 2015; Thondhlana et al. 2016). Note that the two vessels from Kingoyi with high MgO are both Kindangakanzi-type pottery

Refractory crucibles with similar alumina content to Moubiri-type pottery at Kingoyi are known elsewhere in sub-Saharan Africa, namely crucibles used for non-ferrous metallurgy at Great Zimbabwe (Bandama et al. 2017) and Shankare, South Africa (Thondhlana et al. 2016), and glassmaking crucibles of eleventh–fifteenth century Ile-Ife, Nigeria (Babalola et al. 2018) (Fig. 10). These all display greater refractoriness than crucibles from Mapungubwe in southern Africa (Chirikure et al. 2015). Beyond African contexts, high-alumina clays were selected and used to make refractory crucibles from the Roman period onward in Europe, best typified by post-Medieval Hessian examples, and in vessels used for eighth–twelfth century Central Asian crucible steel (Hein et al. 2019; Martinón-Torres et al. 2006, 2008; Martinón-Torres and Rehren 2009, 2014; Rehren and Papachristou 2003).

**Fig. 11:**
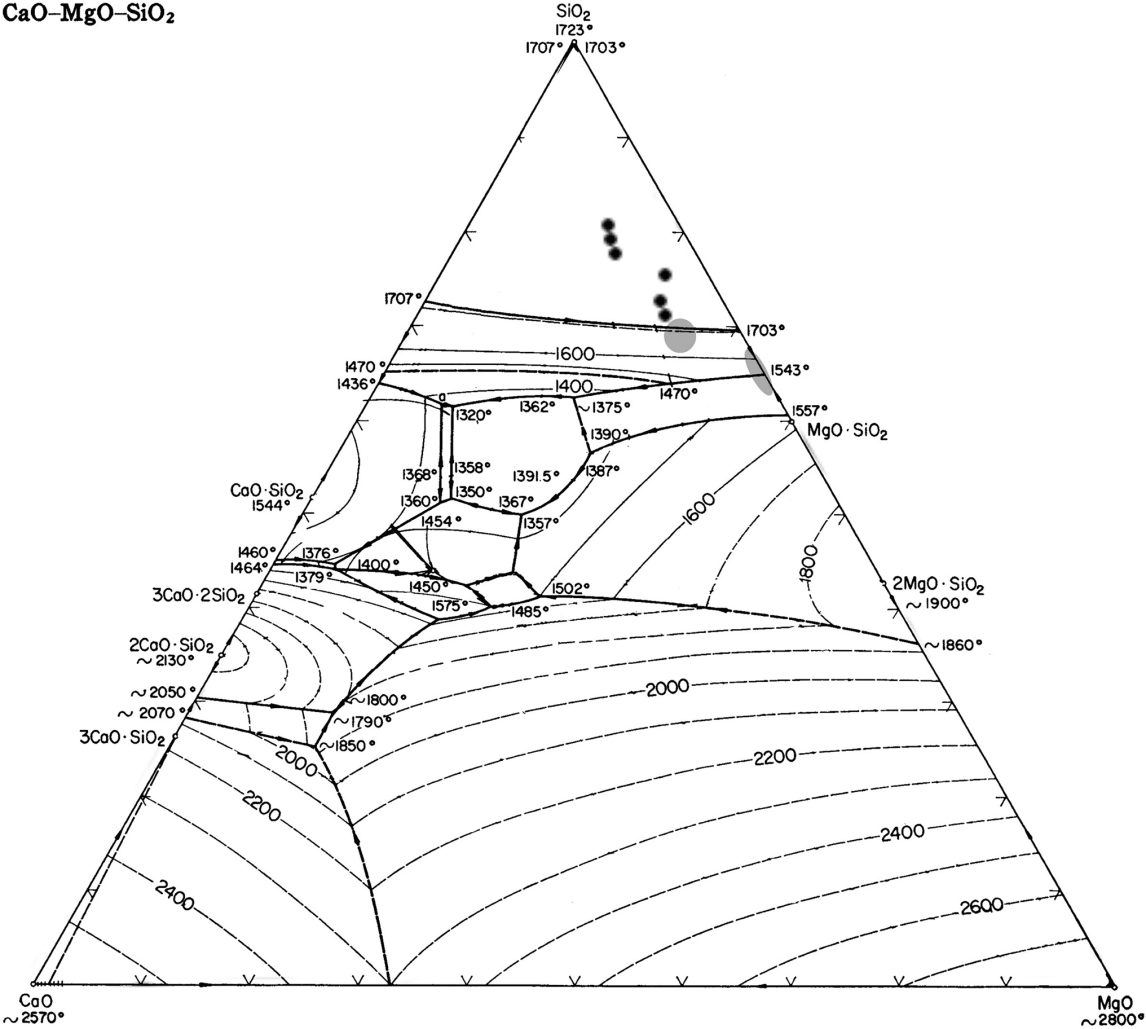
Kindangakanzi-style pottery (black dots) shown on (Thornton and Rehren 2009, Fig. 15): ternary plot of CaO-MgO-SiO_2_ system with Tepe Hissar refractory crucible (grey circle) and the composition of pure steatite (grey ellipse). Al_2_O_3_ has been added to SiO_2_ and FeO to CaO in keeping with the Tepe Hissar comparison. The actual melting temperature of the pottery would be lower based on other oxide impurities, but the point stands that the Kindangakanzi-style pottery is exceptionally refractory due to its high magnesia content. This and all subsequent ternary projections made using Tri-plot Excel extension (Graham and Midgley 2000)

The magnesia-rich clay of Kindangakanzi-type pottery used as crucibles around Boko-Songho, however, is unparalleled within Africa. The only other known archaeological examples of a magnesia-rich refractory crucible come from fourth millennium BCE Tepe Hissar, Iran, where crushed talc was added to local clay not naturally including talc to produce a refractory crucible heated externally for arsenical copper metallurgy (Thornton and Rehren 2009), and the talc-tempered crucibles of the Iron Age Trans-Urals, Russia (Stepanov et al. 2021). The situation is different here in that Kindangakanzi-type vessels were made first as decorated pottery, not as purpose-made crucibles, and that the clay was naturally talc-based. Nevertheless, the use of talc-based clays for Kindangakanzi-type pottery and their subsequent reuse as crucibles represents a unique technical choice in African metallurgy that afforded exceptionally beneficial refractory properties. This is seen in the CaO-MgO-SiO_2_ ternary phase diagram, which indicates that Kindangakanzi-type pottery could withstand high temperatures (Fig. 11). Other pottery types made with talc rich clay have been reported in the region (Cranshof et al. 2018; Denbow 2014; Nikis 2018a, p. 157; Tsoupra et al. 2022).

Both types of pottery, then, afforded highly refractory properties suitable for crucible metallurgy; at less than 12 mm, the vessel walls of both types are relatively thin compared to typical smelting furnaces/crucibles. In terms of the chaîne opératoire, the reuse of domestic pottery as crucibles suggests that the production of the vessels was a separate process from the metallurgical activity.

### Metallurgical debris

Despite the lack of extant finished artefacts, metal prills trapped within the crucible/tuyère slag residues and slag lumps clearly indicate that the target and product of smelting at both Kingoyi and Kindangakanzi was unalloyed copper. Impurities derived from the raw materials carried through the process and distinguish the two types of production.

**Fig. 12:**
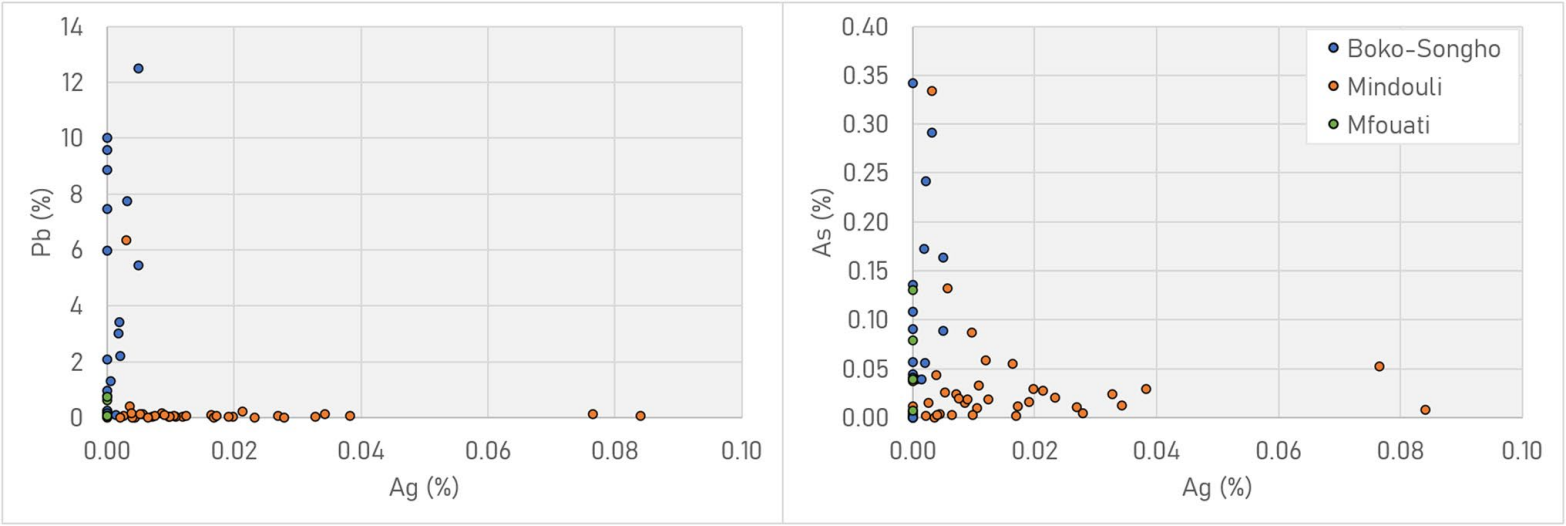
pXRF plot of Ag vs Pb (L) and vs As (R) for all metallurgical material (ores, crucible/tuyère/furnace residues, slag lumps). Note the sharp distinction among Pb and Ag for Boko-Songho (including Kindangakanzi) and Mindouli (including Kingoyi)

pXRF screening indicated that metallurgical debris from the Boko-Songho region could be distinguished from Mindouli by the concentrations of lead, silver and, to a lesser degree, arsenic (Fig. 12). Ores around Boko-Songho contain variable amounts of lead, meaning that slags and crucible/tuyère residues contain these impurities in enriched amounts. The same is true for silver in ores from Mindouli, where minor/trace quantities in the ore are enriched in the metal (Hauptmann 2007, p. 205). Debris from Mfouati, located between the two regions, is more consistent with Boko-Songho in that it contains lead, but it also contains small amounts of silver.

**Fig. 13:**
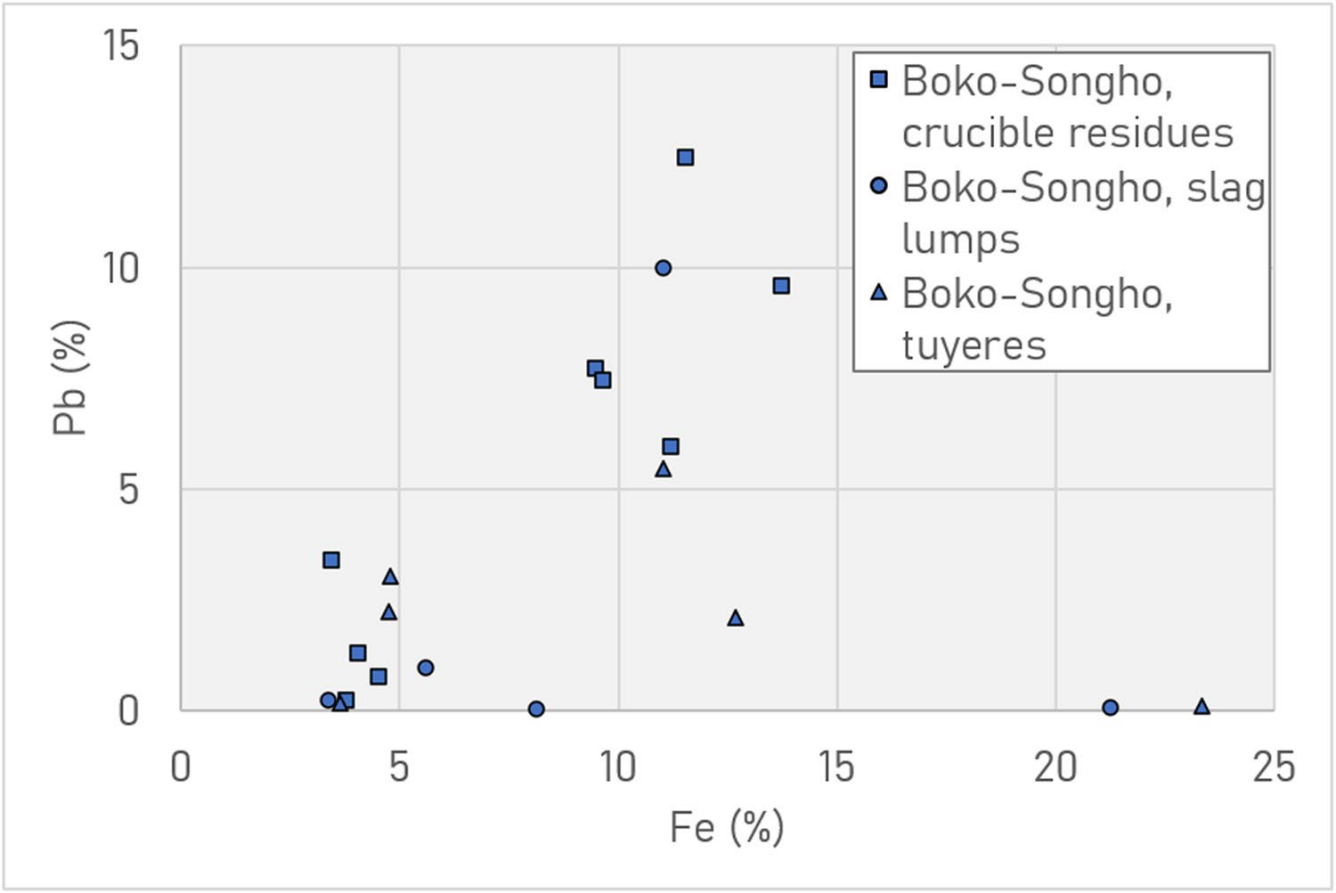
pXRF plot of Fe vs Pb in the metallurgical debris from the Boko-Songho region, including the site of Kindangakanzi. Note the wide distribution of both crucible residues and slag lumps. It is unclear whether there are two groups (low iron/lead, high iron/lead) or whether it is simply a spectrum

**Fig. 14:**
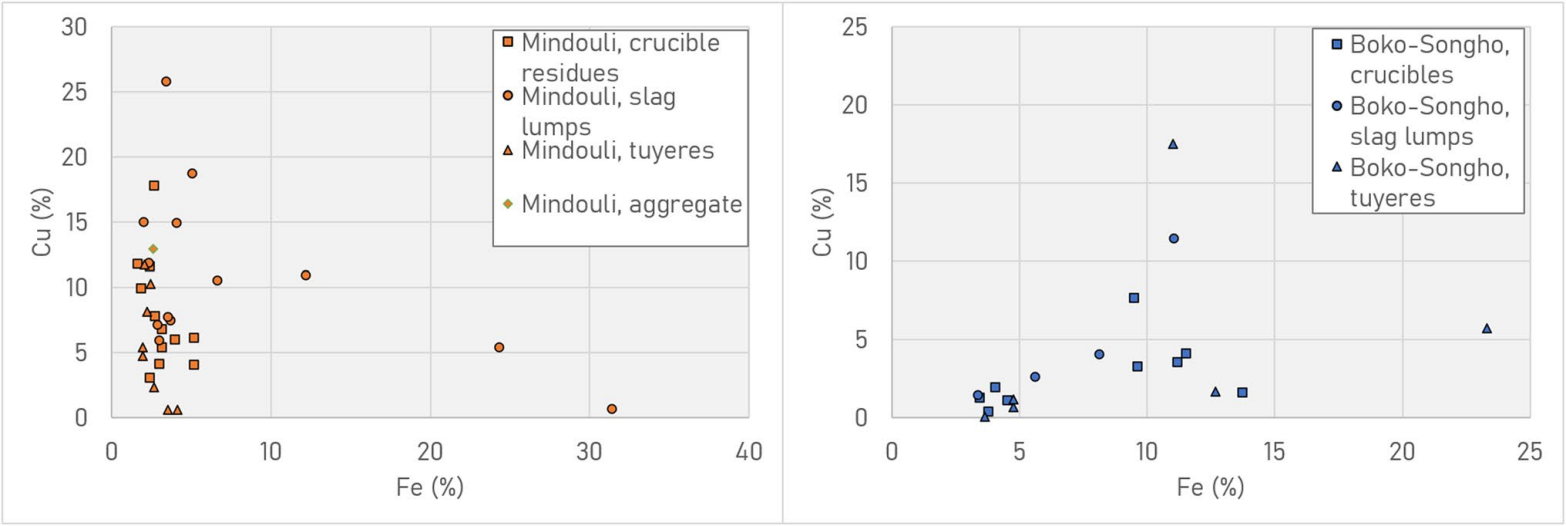
pXRF plots of Fe vs Cu for the region of Mindouli, including Kingoyi (top), and Boko-Songho, including Kindangakanzi (bottom). Note the overlap in crucible residues/slag lumps. Note also that Mindouli debris is relatively consistent in its concentration of iron (< 5%), while there is a greater range around Boko-Songho

There are conceivably two groups within the Boko-Songho material (Fig. 13): one clustering around 5% iron and less than 2% lead, the other ca. 10–15% iron and 6–12% lead. However, this may simply be an artefact of the sampling process and the spectrum of concentrations may be continuous. Around Mindouli, the amount of iron in debris is generally low (< 5%). The fact that at both sites crucible residues and slag lumps are broadly similar compositionally is significant (Fig. 14).

### Crucible slag layers and slag lumps

Crucibles at both Kindangakanzi and Kingoyi display an absence of any external marker of heat and the presence of bloating at the interior, albeit limited, points to an internal heating of the vessels. Furthermore, the slag in general has formed at the rim or shoulder of the vessels, indicating they were likely used in a complete or nearly complete state with the charge filling most of the vessel.

The fragmentation of crucibles at both Kingoyi and Kindangakanzi, evident in the typical sherd size of only a few cm max dimension, and the lack of extant bases for Kindangakanzi-type pottery, may suggest breaking of the crucibles to extract metal. The significant erosion of both sites, however, leaves open the possibility of post-depositional alteration.

Surviving unslagged vessels from other sites (Fig. 4) allow for an estimate using the ULB capacity calculator (Engels et al. 2009). A typical type 1 Moubiri vessel could hold ca. 0.9 L from base to rim. The largest surviving Kindangakanzi-type vessel, despite only stretching from rim to shoulder, could hold over 2.8 L, and thus a substantially larger charge when used as a crucible (Supplementary information).

#### Kingoyi

Kingoyi crucible slag residues vary in thickness between ca. 0.5 and 7.6 mm and in surface colour, with localised areas of green and red copper compounds. In section, they are glassy with large voids (< 2.8 mm), numerous metal prills, and discrete mineral grains. The refractory Moubiri-type pottery performed well under the heat of the metallurgical reaction, retaining angular porosity and forming a sharp separation between slag layer and crucible, evincing relatively little interaction. There is no evidence for the addition of a protective layer of clay on the interior, as initially hypothesised (Nikis 2018a, pp. 334–338).

The Kingoyi slag lumps are generally small in size (< 6 cm) and macroscopically heterogeneous, with angular, porous surfaces, and an appearance of viscosity. Surface colours include combinations of black/orange and areas of green and red copper compounds. There are numerous visible charcoal impressions, as well as mineral and rock grains (< 1.4 cm, MKU3b14_5). MKU3b14_5 displays a more regular, curving surface, possibly from contact with the wall of a crucible.

The SEM–EDS bulk chemical data (Table 5) indicate a high loss of copper into the slag for both slag layers and lumps (up to 30.5% CuO, MKU3b15_13), a trend paralleled in the pXRF screening data. MKU3b14_3 contains the most iron (10.0% FeO) and is the only sample enriched in iron in the slag relative to the ceramic (Table 4). The general absence of iron is surprising, given that ferrous copper ores are known around Kingoyi, e.g. MKU3b14_9, and that the addition of iron to the charge would have facilitated better slag formation.

**Table 4:** Enrichment values for slag layers of technical ceramics, calculated from average composition values of ceramics (Table 3) and slag layers (Table 5). Net positive values for silica and iron and net negative values for magnesia and lime indicate the contribution of material beyond that simply melted from the ceramic (i.e. present in the charge). Absolute values in the slag are diluted by heavy metals, mainly copper, hence the need to look at relative proportions. Despite the relatively limited interaction between the technical ceramics and slags studied here, we use alumina as a denominator, hence assuming that the bulk of the alumina in the slag would derive from ceramic contributions

Site	Sample	Type	SiO_2_/Al_2_O_3_ ceramic	Slag	*Net*	Al_2_O_3_/MgO ceramic	Slag	*Net*	FeO/Al_2_O_3_ ceramic	Slag	*Net*	SiO_2_/CaO ceramic	Slag	*Net*
Kindangakanzi	KNA14_11	Crucible	5.1	7.3	*2.3*	0.5	1.0	*0.5*	0.4	0.6	*0.2*	66.0	45.5	* − 20.4*
Kindangakanzi	KNA14_29	Crucible	6.1	7.6	*1.5*	0.5	0.6	*0.1*	0.3	3.1	*2.8*	148.9	4.9	* − 144.1*
Kindangakanzi	KNA14_9	Crucible	5.4	7.7	*2.3*	0.9	0.7	* − 0.2*	0.4	1.4	*0.9*	39.8	3.3	* − 36.5*
Kindangakanzi	KNA14_37	Furnace	2.7	3.1	*0.5*	25.1	15.3	* − 9.7*	0.3	0.3	*0.0*	270.1	194.5	* − 75.6*
Kindangakanzi	KNA14_10	Tuyère	3.1	4.3	*1.2*	23.0	6.4	* − 16.6*	0.4	2.3	*2.0*	737.9	16.4	* − 721.4*
Kingoyi	MKU3b14_3	Crucible	5.6	11.2	*5.6*	0.5	1.2	*0.7*	0.4	2.1	*1.8*	42.7	4.2	* − 38.5*
Kingoyi	MKU3b15_13	Crucible	2.1	6.9	*4.8*	33.4	1.6	* − 31.8*	0.2	0.5	*0.3*	410.2	5.0	* − 405.2*
Kingoyi	MKU3b15_5	Crucible	3.7	8.8	*5.1*	12.0	0.8	* − 11.2*	0.3	0.5	*0.2*	1075.2	4.4	* − 1070.8*
Kingoyi	MKU3b15_2	Tuyère	4.8	5.8	*1.0*	61.0	3.8	* − 57.2*	0.3	0.4	*0.1*	698.6	9.9	* − 688.7*

**Table 5:** Bulk composition for metallurgical debris (crucible/tuyère/furnace residues, slag lumps, ore), gathered via SEM–EDS from averages of at least three areas. Oxygen added via stoichiometry; all values normalised to 100 wt%

Sample	Type	MgO	Al_2_O_3_	SiO_2_	P_2_O_5_	K_2_O	CaO	TiO_2_	MnO	FeO	CoO	CuO	ZnO	PbO
KNA14_11	Crucible	7.9	8.1	59.3	0.7	2.7	1.3	0.5	0.2	5.0		7.6	1.0	5.7
KNA14_29	Crucible	5.5	3.4	25.6	7.3	2.9	5.3	0.1	1.1	10.5		5.4	3.3	29.2
KNA14_9	Crucible	6.3	4.5	34.8	1.5	2.7	10.4	0.2	1.0	6.1		24.5		7.9
KNA14_37	Furnace	0.9	14.3	44.7	0.4	1.2	0.2	0.3	0.0	4.0		30.5		3.4
KNA14_25	Slag	0.8	2.3	27.3	0.6	0.5	0.6	0.1	1.3	26.6	0.3	39.6		
KNA14_26	Slag	5.8	3.4	33.9	1.7	3.3	6.4	0.2	0.6	11.9		14.1	2.1	16.5
KNA14_10	Tuyère	1.5	9.4	39.9	0.9	1.5	2.4	0.3	1.6	22.0		20.5		
MKU3b14_3	Crucible	4.0	4.7	52.8	1.3	2.5	12.5	0.4	2.8	10.0		9.0		
MKU3b15_13	Crucible	3.8	6.3	43.4	1.0	1.4	8.6	0.3	1.1	3.1		30.5		
MKU3b15_5	Crucible	6.8	5.3	46.8	1.0	2.3	10.6	0.3	1.9	2.8		22.3		
MKU3b14_9	Ore	0.5	0.2	19.2	0.1	0.0	0.2	0.1	0.2	44.7		34.9		
MKU3b14_5	Slag	7.6	5.7	53.9	1.2	1.4	7.8	0.4	1.7	6.9		13.4		
MKU3b14_8	Slag	12.5	2.5	41.0	1.4	3.2	6.3	0.2	0.9	1.7		30.3		
MKU3b15_2	Tuyère	2.2	8.4	48.9	1.5	2.8	4.9	0.5	0.9	3.3		26.7		

Slags contain relatively large enrichments of MgO and CaO, perhaps from the presence of dolomite/dolomitic calcite gangue. Silica values are also enriched relative to the ceramic, correlating with visible grains of trapped quartz and relict ores. Manganese values (< 2.8% MnO, MKU3b14_3) are consistent with its known presence in Niari ores. All of these elements are consistent with gangue present in locally available ores, making the addition of flux unlikely.

**Fig. 15:**
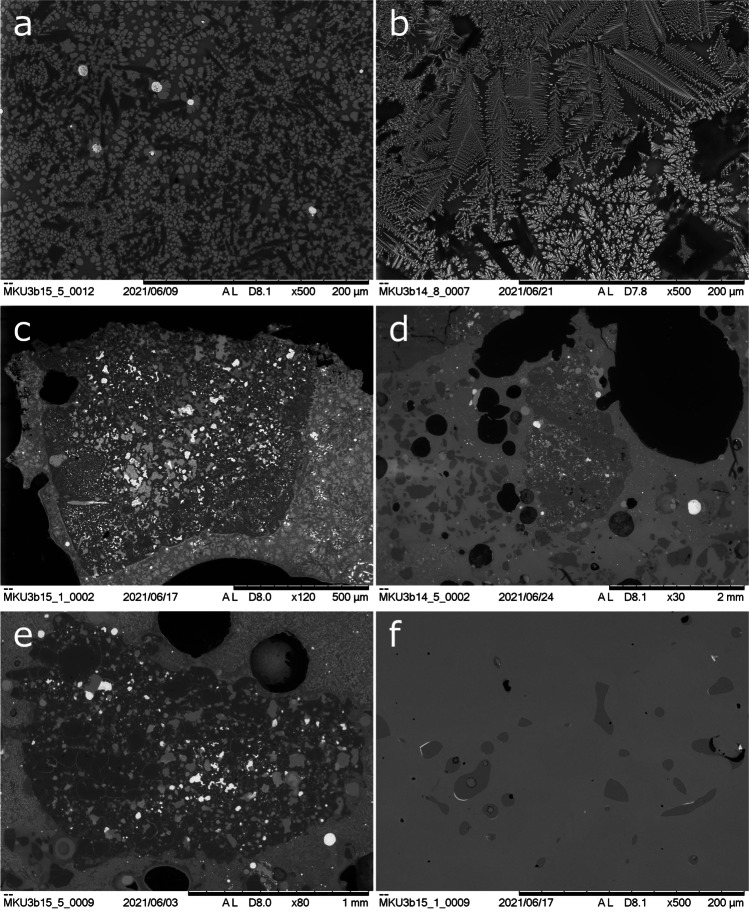
BSE images of Kingoyi samples. **a** Cuprite phases (mid-grey) in crucible slag MKU3b15_5. **b** Cuprite dendrites in slag lump MKU3b14_8. **c** Relict ore fragment (partially reacted quartz and copper) in crucible MKU3b15_13. **d** The same in slag lump MKU3b14_5. **e** The same in crucible MKU3b15_5. **f** Sulphide inclusions (dark grey) and metallic silver impurities (bright white) within a large copper prill of crucible MKU3b15_13

Low iron content means that Kingoyi slags are not fayalitic in structure (Table 6). They are instead characterised by glassy phases and inclusions of cuprite and pyroxenes, e.g. diopside (Fig. 15a, b). Cuprite dendrites indicate high temperatures, ca. 1200° C, and relatively poor reduction of malachite (Hauptmann 2020, p. 270; Rovira 2005, p. 91). The presence of calcium- and magnesium-bearing pyroxenes (diopside) is consistent with the elevated Ca and Mg values of the slags in suggesting calcareous/dolomitic gangue. There are also copper silicate phases related to the partial reduction of ores (Fig. 15c–e). A lack of high-temperature silica polymorphs (e.g. tridymite, cristobalite) in the FTIR analysis of crucible slag (MKU3b15_13) and slag lumps (MKU3b14_5) indicates that high temperature reactions were not sustained for very long periods (Supplementary information).

**Table 6:** Slag phases identified via SEM–EDS. *Lead-rich glass in KNA14_29. Abbreviations: *Del.* delafossite, *Spl.* spinel, *En.* enstatite, *Pyr.* pyroxene, *Di.* diopside, *Ol.* olivine, *Lct.* leucite

Sample	Type	CuO	Cu_2_O	CuSi	PbO	Del	Spl	En	Pyr. (e.g. Di.)	Ol	Lct	Glass	Ore
KNA14_11	Crucible	x						x				x	x
KNA14_29	Crucible	x					x		x		x	x*	
KNA14_9	Crucible	x	x					x	x			x	x
KNA14_37	Furnace	x			x							x	
KNA14_25	Slag	x		x		x	x						
KNA14_26	Slag	x					x			x		x	x
MKU3b14_3	Crucible	x							x			x	x
MKU3b15_13	Crucible	x	x						x			x	x
MKU3b15_5	Crucible	x	x	x					x			x	x
MKU3b14_5	Slag	x		x								x	x
MKU3b14_8	Slag	x	x	x				x	x			x	x

Relict ore grains are evident in all slag samples from Kingoyi (Fig. 15c–e) (< 4.3 mm, MKU3b14_5). Ore minerals contain copper prills within quartz, and, in MKU3b14_8, talc. Metal prills within the Kingoyi slags are invariably unalloyed copper. There are several size ranges, with prills < 100 μm in diameter throughout, and some larger prills up to a few mm in diameter (< 6.9 mm, MKU3b15_13). Unfortunately, the surrounding volume effect of the EDS system precluded quantification, necessitating a qualitative assessment of impurities (Table 7). Similarly, it is not possible to assess the relationship between the amount of iron in the copper prills as a proxy for understanding smelting conditions (Cooke and Aschenbrenner 1975; Craddock and Meeks 1987).

**Table 7:** Qualitative presence of impurities within copper prills identified by SEM–EDS. The high uptake of surrounding volume by the SEM–EDS system precludes meaningful quantification and as such it is more appropriate to note the presence/absence of various impurities. This matrix effect also makes it impossible to assess the percentage of iron in copper as a proxy for understanding smelting conditions

Site	Sample	Type	S	As	Ag	Pb
Kindangakanzi	KNA14_11	Crucible		x		x
Kindangakanzi	KNA14_29	Crucible				x
Kindangakanzi	KNA14_9	Crucible				x
Kindangakanzi	KNA14_37	Furnace				x
Kindangakanzi	KNA14_25	Slag				
Kindangakanzi	KNA14_26	Slag				x
Kindangakanzi	KNA14_10	Tuyère				
Kingoyi	MKU3b14_3	Crucible	x	x		
Kingoyi	MKU3b15_13	Crucible	x		x	
Kingoyi	MKU3b15_5	Crucible	x			
Kingoyi	MKU3b14_5	Slag	x		x	
Kingoyi	MKU3b14_8	Slag				
Kingoyi	MKU3b15_2	Tuyère			x	

Small copper sulphide inclusions (Fig. 15f) in prills in all three crucibles and one slag lump likely represent the persistence of trace amounts of sulphur within the secondary ores (malachite). While the exploitation of sulphide ores is not attested ethnographically in sub-Saharan Africa (Killick 2016; Miller 2002), the practice cannot be excluded based on the present data; indeed, sulphide ores are present in the Niari mineralisations and addition of small quantities of sulphide ores is attested in nineteenth century descriptions of the same area (Barrat 1895, p. 464; Loir 1911; Rademakers et al. 2018). Silver is evident in individual spot analyses, particularly in partially corroded areas where noble inclusions are more resilient (Martinón-Torres and Rehren 2014, p. 128). Its presence is consistent with the overall signature for natural impurities in ores from around Mindouli, as is arsenic within a prill from crucible slag MKU3b14_3.

#### Kindangakanzi

Crucible slag residues at Kindangakanzi are between 0.6 and 15.7 mm thick and range in colour from black to orange, with localised areas of red, and less frequently, green copper compounds. The interface between the ceramic and slag is a sharp horizon, indicating limited interaction (Fig. 8), and the slag layers are generally homogeneously glassy with numerous metallic prills, limited porosity, and limited mineral inclusions.

KNA14_11, with a thick slag layer characterised by large porosities (< 2.1 mm diameter) and a greater number of quartz inclusions and partial vitrification of the ceramic, is an exception, perhaps indicating a lengthier reaction or multiple uses. KNA14_11 also displays a degree of heterogeneity, with possible layering of slag around the largest metallic prill and largest void, in the middle of the residue (Supplementary information). KNA14_29 is slagged on both sides, as are several other sherds within the assemblage. The slag flows over a fresh break, not the rim, indicating that the vessel was either used in a fragmentary state or broke during use.

Kindangakanzi slag lumps are < 5 cm in maximum dimension. Macroscopically, they appear denser and more homogeneous than those from Kingoyi. Porosity is more localised and smaller, and surfaces are smoother with more signs of fluidity. The colours are mostly black and, less frequently, orange, with occasional small patches of red/green copper compounds. There are small mineral grains present and metal prills of varying sizes throughout. Charcoal impressions are present on many of the lumps.

Slags from Kindangakanzi display a wider variability in their composition, in particular concentrations of iron and lead, as shown in Table 5, a trend mirrored in the wider pXRF dataset (Figs. 13 and 14).

Iron is again generally low. Crucible KNA14_29 is atypical in being enriched in iron in the slag relative to the ceramic, suggesting a ferrous component to the gangue. KNA14_25 contains the highest amount of iron (26.6% FeO). The amount of copper trapped in the slag varies between 5.4% CuO (KNA14_29) and 39.6% (KNA14_25). The absence of lead in slag lump KNA14_25 is of particular interest, as it is otherwise present in the crucible slags and the other slag lump. These differences may be a reflection of the nature of mineralisation, with different parts of the exploited deposit richer or poorer in lead and iron.

Similarly, the small amounts of zinc in the slag likely derive from the Niari’s complex mineralisations and zinc’s extreme volatility and free energy of oxidation, meaning that even trace quantities result in disproportionate enrichment in slag and technical ceramics (Kearns et al. 2010).

**Fig. 16:**
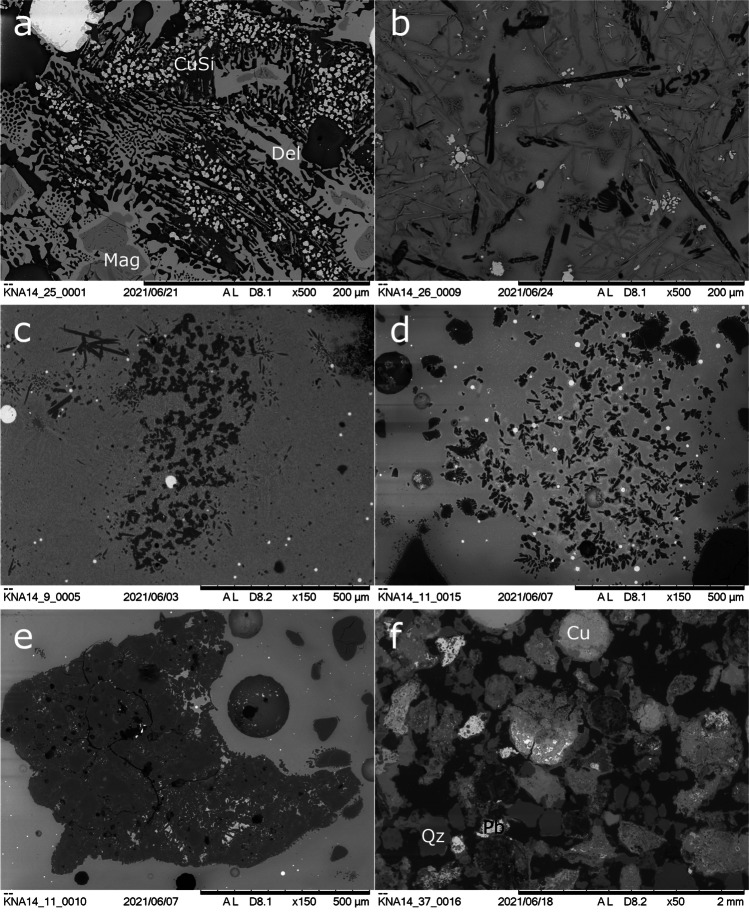
BSE images of Kindangakanzi samples. **a** Magnetite spinels (Mag) and delafossite (Del) in slag lump KNA14_25 together with semi-reacted copper silicate phases. **b** Forsterite (dark grey) in slag lump KNA14_26. **c** A mineral pseudomorph of enstatite in crucible KNA14_9. **d** Mineral pseudomorph of quartz altering to tridymite in crucible KNA14_11. **e** A relict ore comprised of quartz and copper- and lead-rich phases in crucible KNA14_11. **f** The furnace residue KNA14_37 shows a variety of quartz inclusions, and weathered copper and lead prills

Kindangakanzi slags contain glassy phases together with various pyroxenes but are otherwise more varied than their Kingoyi counterparts (Table 6). There is only one sample with copper silicate (KNA14_25), however, and one sample with cuprite (KNA14_9). The limited presence of these phases indicates on the whole a more reducing atmosphere. However, where iron is present, it has formed magnetite spinels, and, in the case of KNA14_25, spinels with delafossite rims (Fig. 16a), characteristic of relatively oxidising conditions and typical of relatively inefficient crucible smelting slags (Hauptmann 2020, p. 269). KNA14_26 lacks delafossite but contains the olivine phase forsterite, indicative of an abundance of magnesium relative to iron within the slag and also a relatively high temperature (Fig. 16b).

The presence of copper silicate in KNA14_25 indicates the partial reduction of ores with quartz gangue. Mineral pseudomorphs of enstatite (Fig. 16c), formed from the thermal alteration of talc above 800° C reflect additional gangue (Deer et al. 2013; Liu et al. 2014).

**Fig. 17:**
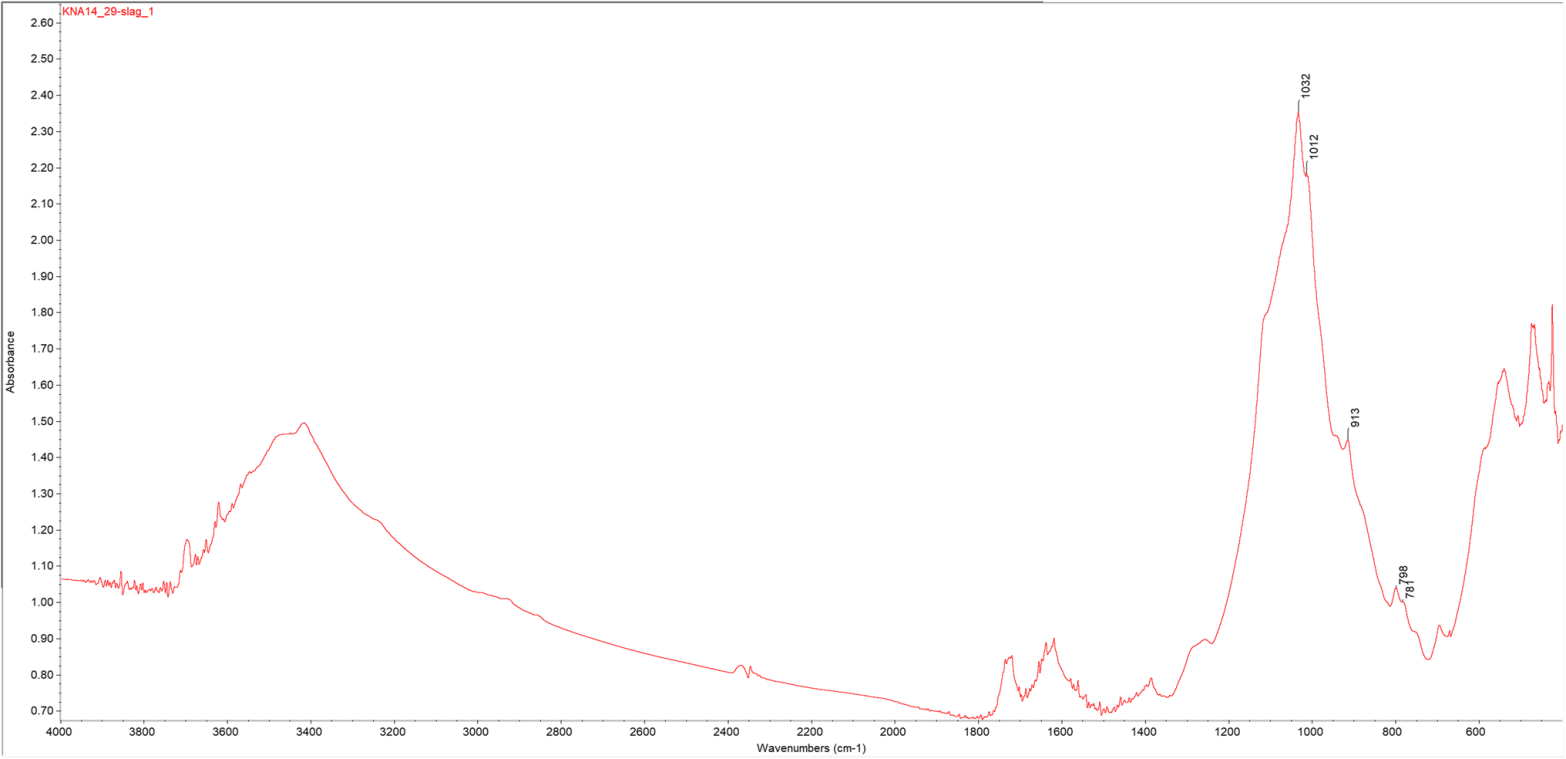
FTIR spectrum of slag layer from crucible KNA14_29. Note that the quartz doublet at 798, 781 is not as pronounced and is developing into the single peak of the high-temperature silica polymorph tridymite, a transformation that occurs between ca. 800 and 1050° C (Hauptmann 2020, p. 255; Weiner 2010, p. 298)

As at Kingoyi, there is a lack of high-temperature silica phases in the FTIR spectra. The quartz doublet at 798 and 779 cm^−1^ in crucible slag KNA14_29 appears to be transforming to the 791 single peak of tridymite (Fig. 17). This transformation occurs between 800 and 1050° C depending on fluxes (Hauptmann 2020, p. 255; Weiner 2010, p. 298). The peak is poorly developed and the spectrum lacks the other distinctive peaks of tridymite, possibly indicating a relatively short reaction. This is corroborated by the incipient alteration of quartz to tridymite visible within the slag layer of crucible KNA14_11 (Fig. 16d). Altogether, this suggests the lack of sustained high temperatures.

There are trapped ore fragments with quartz gangue in two of the crucibles and one of the slag lumps (< 4.5 mm, KNA14_26) (Table 6). Within KNA14_11, the ore also contains traces of lead, typical of ores around Boko-Songho (Fig. 16e). As Kindangakanzi-pottery is talc-based, large unreacted quartz grains are residual from the charge. Silica enrichment values corroborate this (Table 4).

There are rounded copper prills < 100 μm in diameter evenly distributed within the slag of all samples (< 2.2 mm, KNA14_29). With the notable exception of slag lump KNA14_25, there is typically a small percentage of lead in the prills (Table 7). It is not present in significant enough quantities to form phases within the copper, instead existing as small impurities. KNA14_11 also contained two prills with small amounts of arsenic.

### Tuyère and furnace residues

Tuyère slag residues are illustrative in confirming a link to copper metallurgy but are not as representative of reaction conditions as crucible residues or slag lumps, as they are in less contact with the charge. Their phase identification is not included. The slag layer on Kingoyi tuyère MKU3b15_2 contains copper prills with trace silver present, consistent with other debris at the site.

The slag layer on Kindangakanzi tuyère KNA14_10 contains large copper prills and 20.5% CuO, confirming the link to copper metallurgy. This tuyère contains the highest concentration of iron of residues from the site (22.0% FeO, Table 5). Unlike other residues at Kindangakanzi, this tuyère does not contain lead. As even small quantities of lead disproportionately contaminate technical ceramics, it is safe to assume its absence (Kearns et al. 2010). Similarly, the prills, up to ca. 0.8 mm in size, contain no traces of lead (Table 5).

The residue from the Kindangakanzi furnace (KNA14_37) is ca. 5.2 mm thick, irregular and friable. There are distinct rounded prills and mineral grains visible within the matrix (Fig. 16f). The furnace residue is compositionally akin to the crucible residues, i.e. high copper, relatively low iron, and moderate lead. Iron and silica are not enriched in the slag relative to the ceramic, suggesting a lack of ferrous gangue (Table 4). Magnesia and lime levels are low overall but enriched relative to the ceramic wall of the furnace, likely deriving from a combination of fuel and magnesia-rich gangue. Copper prills are generally large (< 0.9 mm, most ca. 0.3–0.8 mm in diameter) and contain small amounts of lead. Many display hexagonal grain boundaries outlined by copper oxide corrosion, evincing a slow cooling time. Grains of lead chlorophosphate (< 0.5 mm), formed from the weathering of metallic lead, and lead silicate slag grains indicate lead in the charge.

## Discussion

### Metallurgical chaînes opératoires

At a broad glance, copper production at Kingoyi and Kindangakanzi appears similar, in that decorated pottery was reused as crucibles for the smelting of copper based on liquefaction of the metal, not the slag, and air was supplied with tuyères and bellows. The use of tuyères for crucible smelting is perhaps surprising, as it would have been difficult to consistently maintain the position of straight tuyères pointed downward into the mouth of the crucibles, as opposed to their usual fixed positioning at the base of a furnace. It is unlikely that the tuyères were simply for the furnace, as there is no evidence for a furnace at Kingoyi and at Kindangakanzi the tuyère slag residue contains no lead, which is present in the furnace residue.

The crucible slag residues and slag lumps from Kingoyi and Kindangakanzi are characterised by a relatively high amount of trapped copper and by phases associated with variable redox conditions (e.g. cuprite, delafossite). The low iron and high copper content of the slags is more typical of a melting process (Bachmann 1982). However, the presence of relict ore grains in all three of the Kingoyi crucibles and all but one of those from Kindangakanzi clearly indicates smelting in these vessels. Quartz grains and the enrichment values for silica in the slag relative to the ceramic indicate the introduction of quartz gangue into the charge. Similarly, the enrichment in the slag of manganese oxide, known as a minor/trace element in Niari ores, and, in the case of Kindangakanzi, the similar enrichment of zinc- and lead-oxides from the ores around Boko-Songho, indicates that the slag derives from smelting. The crucibles were heated at the interior for these reactions, likely as complete vessels, given that slag layers gather near the shoulders and rims. The thickness of the crucible residues is again consistent with smelting.

The exception to this pattern is KNA14_29, broken and slagged on both sides, and with the least interaction between ceramic body and slag layer. There are two other such samples with overflowing slag examined via pXRF (KNA14_30, KNA14_34). These constitute three of the four members of the possible higher lead and iron subgroup at Kindangakanzi discussed above (Fig. 13). The slag in KNA14_29 is characterised by lead silicate and an absence of quartz or relict ore grains (Supplementary information). The higher concentration of iron and lead and slagging on both sides may indicate its use as a skimmer in a refining process to remove floating metal oxide dross. Such slag skimmers are found at Mapungubwe and Marothodi (Calabrese 2000; Hall et al. 2006). However, the slag layer of KNA14_29 is enriched in silica, iron, manganese, and zinc, indicating it may also have been used in smelting. Any breakage during this process would have encouraged oxidation, and as lead both oxidises and bonds with silica easily, this may have resulted in the formation of the observed lead silicate (Kearns et al. 2010). Both slag skimming and smelting are viable possibilities, and it is difficult to determine how this vessel was used.

**Fig. 18:**
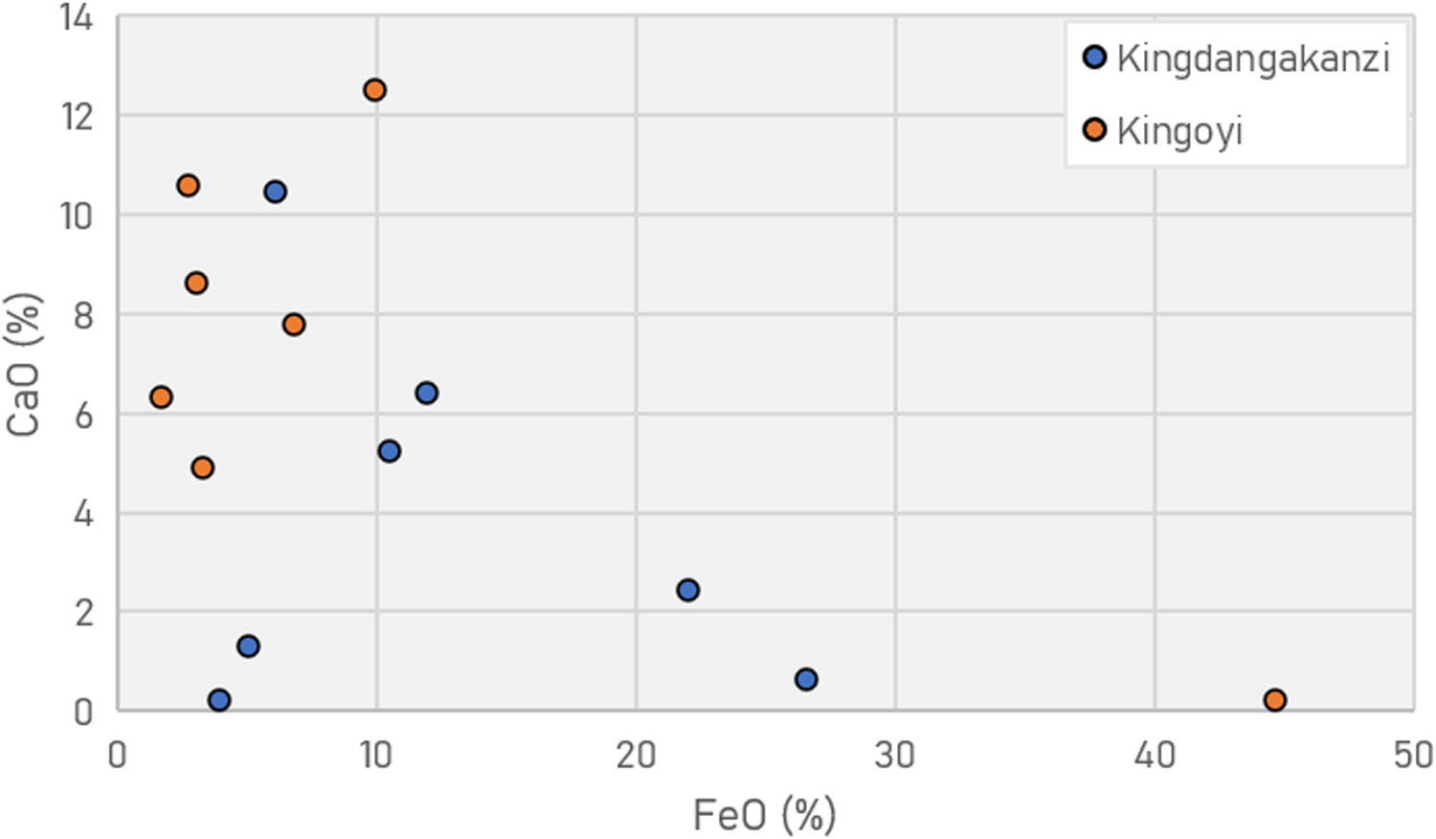
Plot of SEM–EDS data of metallurgical debris showing that, in general, Kingoyi slags and residues contain more CaO and less FeO than those from Kindangakanzi. The Kingoyi outlier is the iron-rich ore MKU3b14_9

The high concentrations of MgO and CaO and the high ratios of MgO:CaO in slags from both sites, and particularly at Kindangakanzi (6.1, KNA14_11), indicate a source of magnesia beyond the fuel. MgO:CaO ratio values for fuel ash are generally low, e.g. pine (0.3) (Misra et al. 1993, Table 4). A likely source is some combination of talc and dolomitised calcite of the Schisto-Calcaire Formation, depending on the CaO concentration. High MgO and CaO values derived from the presence of dolomitic gangue are known from Chalcolithic Faynan (Hauptmann 2007, p. 163) and near Agadez, Niger in the first millennium BCE (Killick et al. 1988). At Kingoyi, meanwhile, the higher CaO values and enrichment values suggest the presence of (dolomitised) calcite. Analysis of the ores revealed talc/calcite gangue, while relict ore grains visible in slags/crucible residues showed talc and also quartz gangue. The inverse correlation of CaO/FeO indicates the calcareous and ferrous components (Fig. 18), with Kingoyi more frequently containing calcareous gangue, and Kindangakanzi showing a more mixed signature.

A key question is the relationship between the slag lumps and the crucible slag residues and whether they derive from the same reaction processes. The qualitative prill data make it difficult to assess differences in composition between lumps and residues, which could clarify the loss of more volatile metals (e.g. zinc, lead, iron) and the persistence of more noble ones (e.g. silver) in more oxidising areas at the centre of the crucible (Murillo-Barroso et al. 2017). The bulk chemistry, however, indicates broad compositional similarities between the crucible residues and slag lumps for both sites. Variability at Kindangakanzi is more pronounced between leaded and unleaded debris than between crucible slag layers/lumps. It is likely that slag lumps at both sites derive from crucible smelting.

The Niari slags exhibit a high viscosity and heterogeneity associated with a poor slag-metal separation. The low iron, high copper, presence of elements enriched from gangue, absence of sulphur, and presence of phases associated with poorly reducing conditions all indicate the smelting of relatively high-grade secondary copper minerals (e.g. malachite) without the addition of ferrous fluxes. Similar crucible smelting slags are attested in Chalcolithic Europe (Hauptmann 2007, pp. 157–180; Murillo-Barroso et al. 2017; Queixalos et al. 1987; Radivojević et al. 2010; Rovira 2002). While there are limited phases indicative of high temperatures (e.g. cuprite dendrites, MKU3b14_8; forsterite, KNA14_26), the lack of well-developed high-temperature silica polymorphs suggests that the reactions did not maintain these high temperatures.

**Fig. 19:**
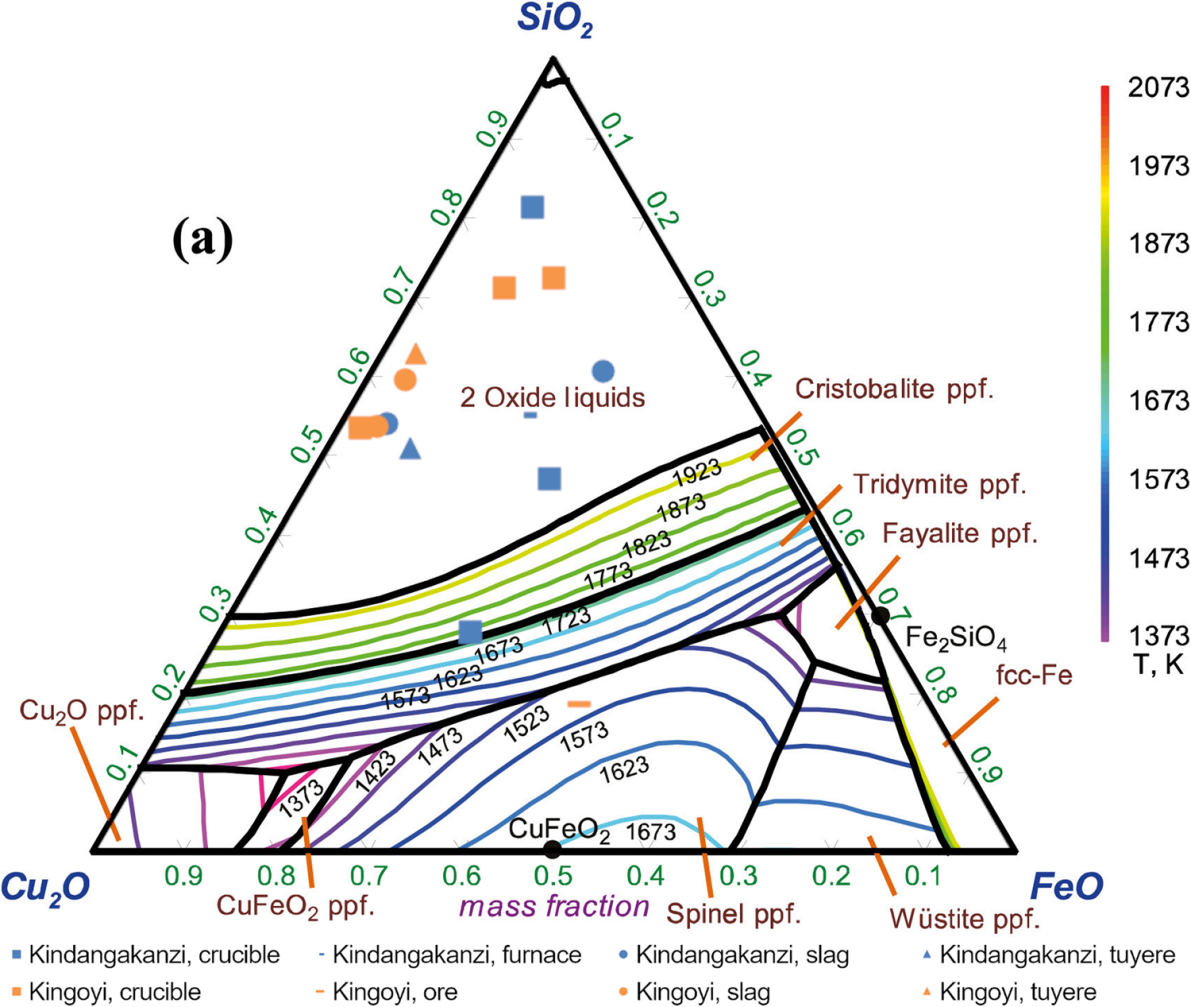
Projection of Kindangakanzi and Kingoyi metallurgical debris (SEM–EDS data) onto Cu2O-SiO2-FeO ternary system, indicating that the composition of the slags is by and large not conducive to a low melting point (Hidayat et al. 2017, Fig. 2a)

The FeO-SiO_2_-CaO ternary phase diagram is not very useful here, given that these oxides constitute only slightly more than 50% of the bulk composition, due to the high copper and low iron and calcium of Niari slags. The ternary plot Cu_2_O-FeO-SiO_2_ (Fig. 19) is used instead to demonstrate that the slag composition for both sites was in general poorly conducive to reaching a fully liquid state at a low temperature The process was essentially the opposite of a tap-slag furnace: rather than removing impurities as liquid slag, temperatures sufficient to melt the metal were reached while the only partially liquified slag remained (Hauptmann 2007, pp. 164–166). The addition of iron to the charge would have lowered the melting point, facilitating greater slag-metal separation. It is noteworthy, then, that at Kingoyi, despite the availability of ferrous copper ores (e.g. MKU3b14_9) smelters appear to have instead chosen ores low in iron.

The chaîne opératoire reconstructed for Kingoyi evinces a possible response to such poor slag-metal separation (Table 8). After smelting, slag could then be crushed to extract metal prills. A fragment of sandstone found at Kingoyi may have served as a stone anvil, and this crushing may have resulted in the formation of a concentrated aggregate found in excavation (Fig. 5a). Nevertheless, any crushed slag still contained copper trapped in the microstructure, evident in the copper silicate and cuprite phases identified via SEM–EDS. Furthermore, not all of the slag may have been crushed. While there are some slags with break fractures at the site, the slag lumps examined here do not display any and are larger compared to crushed slags at other sites, e.g. Shankare (average max dimension of Niari slags, 4.7 cm; of published Shankare samples, 2.1 cm) (Thondhlana et al. 2016, Fig. 6b). This suggests the examined lumps were not crushed, either because crushing was not always practised during this period or perhaps because only certain slags were selected for crushing, conceivably based on weight and perceived yield of metal.

**Table 8:** Comparative table of chaînes opératoires for Kingoyi and Kindangakanzi showing commonalities between the two sites in bold. Note that while there are some common materials (malachite, quartz-based clay), the more substantial and significant similarities in technological decisions lie in processes (the reuse of decorated pottery as crucibles, the use of those crucibles for smelting, and a similar system of air supply)

	Kingoyi	Kindangakanzi
*Potting*	Quartz-based clay Moubiri-type pottery	Talc-based clay Kindangakanzi-type pottery
*Mining*	**Malachite** (quartz, dolomite)	**Malachite**, malachite/galena (quartz, talc)
*Crushing*	Ore, stone hammers/anvils? Selection/rejection of ores	No direct evidence
*Technical ceramics*	**Quartz-based clay** Smaller, less conical tuyères	**Quartz-based clay**, talc-based clay Larger, more conical tuyères
*Smelting*	(Processed?) ore, fuel, **reused decorated pottery** **Crucible smelting** **Bellows and tuyère**	(Processed?) ore, fuel, **reused decorated pottery **and/or furnace **Crucible smelting**, furnace smelting **Bellows and tuyère**
*Slag crushing*	Slag, stone hammers/anvils?, soil aggregate	
*Melting *	Combining casting prills? (no direct evidence)	Refining and slag skimming?

Slag crushing is a common process to maximise metal yield, known elsewhere in sub-Saharan Africa, such as at Shankare, Marothodi, and Drierivier (Hall et al. 2006; Killick et al. 2016; Miller and Sandelowsky 1999; Thondhlana et al. 2016) and in copper production contexts from Chalcolithic Iberia (Bourgarit 2007; Murillo-Barroso et al. 2017; Rovira 2002). This process implies the remelting of retrieved prills into objects or ingots. The studied Kingoyi crucibles were clearly used for smelting, meaning that they were either reused with the formation of minimal melting slag, or crucibles beyond those studied here were used.

At Kindangakanzi, where iron is present in slags it crystallised into magnetite spinels and, in the case of KNA14_25, delafossite. Delafossite is an index mineral for oxidising conditions, and while it is known in melting or refining slags (Bachmann 1982, p. 16), its copresence with copper oxides and magnetite, rather than fayalite, is characteristic of poorly reducing crucible smelting slags (Bourgarit 2007; Burger et al. 2010; Chirikure et al. 2015; Hauptmann 2020, p. 269; Hook et al. 1991; Montes-Landa et al. 2020; Müller et al. 2004; Radivojević and Rehren 2016; Rovira 2002; Shugar 2000).

The presence of lead impurities in the ore, even in small amounts, increased the fluidity of the slag by lowering the melting point of the silica (Hauptmann 2007, p. 168). The limited amount of lead within the slag and the absence of lead-rich phases in the copper prills suggest it was not added deliberately; however, the final product may have been richer in lead than the analysed prills, due to lead concentrating in the denser metal pool at the bottom of the crucible during smelting.

A key question for Kindangakanzi is whether similar ore processing and selection to that at Kingoyi was carried out. Lead-rich ores may have been selected by the miners and smelters or they may have incidentally benefited from the complex ore deposits around Boko-Songho. A related issue concerns the co-presence of both crucible and furnace smelting. The crucibles, tuyère, and slags studied here come from the 0–10 cm layer of SVI, located 12 m to the SE of the furnace. It is conceivable that there were different activity areas on the site, leaving several possibilities:

1. *Different phases*: the radiocarbon analysis of charcoal from within the furnace established a 2σ date of 1457–1627 cal AD for its last use. While the top layers of SVI likely relate to this final phase of the furnace, the site has been eroded. It is possible that the two contexts represent multiple phases of the site, one where the furnace was in use and another where crucibles were used for smelting. Without radiocarbon dating from SVI, it is impossible to refute this possibility.
2. *Different groups*: if furnace and crucible smelting were indeed contemporaneous, the different processes may reflect different groups of people (kin groups? Outsiders/locals?). As with any chronological differentiation, it is difficult at present to assess this possibility.
3. *Different processes for different ores*: the furnace may have been used for the smelting of a different ore, perhaps either more or less lead-rich. The presence of lead within the slag layers of both the furnace and crucibles is clear; however, it is absent in one of the slag lumps (KNA14_25) and in the tuyère residue. There appear to have been ores of varying lead content, but whether these were processed differently is hard to determine.
4. *Different steps of the same process*: furnace smelting could have been followed by crucible refining to remove impurities, namely lead. The account that Olfert Dapper relates of seventeenth century smelting among the Nsundi, geographically imprecise but likely within the Niari, mentions the mixing of multiple ores, resulting in a grey copper that required refining (Dapper 1676; Herbert 1984, p. 142). The presence of lead in the charge of both the furnace and crucibles at Kindangakanzi is in accord with his mention of smelting multiple ores together, likely actually complex ores as analysed by Rademakers et al. (2018). Intentionally leaded copper is known archaeologically and ethnographically around Boko-Songho in the nineteenth–twentieth centuries (Dupont 1889; Pleigneur 1888; Rademakers et al. 2018; Volavka 1998). A two-step process of smelting and refining is also known in the historical copper metallurgy of the Copperbelt (Chaplin 1961; Miller 1994). Such a possibility is unlikely for the fifteenth–seventeenth century at Kindangakanzi, as the evidence for smelting is clear in two of the three examined crucibles from their relict ore grains and enrichment values. Furthermore, the analysis of prills indicates lead was a minor impurity in the copper. If KNA14_29 was used as a slag skimmer, there may, however, have been limited removal of slaggy dross at a later stage, although the evidence is not conclusive.

At present, it is difficult to distinguish between these different possibilities for the organisation of production, beyond likely ruling out a multi-step chaîne opératoire of furnace smelting and crucible refining. While lead was not added deliberately in this period, the sensory experiences of those smelting the lead-rich ores in the fifteenth–seventeenth centuries may have contributed to the later development of leaded copper alloys. Metalworkers surely recognised the effect of lead on the copper, tangible in its working properties and outwardly perceptible in its colour, especially given the importance of copper’s redness and lustre to its social significance within West Central Africa.

## Conclusion: communities of practice

The integration of technical data within a socio-cultural framework begins with describing the step-by-step sequence and variability within this, but also examines why and how choices in materials and technique were made, a key aspect of the chaîne opératoire approach that allows for a consideration of knowledge sharing among and between groups (Gosselain 2016; Martinón-Torres 2002; Pfaffenberger 1998; Sillar and Tite 2000). By way of conclusion, we explore the possibility of exchange of knowledge between people associated with Moubiri-type production at Kingoyi and Kindangakanzi-type at Kindangakanzi, and the role of the mining landscape in facilitating such interaction.

One way to theorise knowledge sharing is via ‘communities of practice’ (Lave and Wenger 1991; Roddick and Stahl 2016; Wenger 1998). Communities of practice are characterised by shared skills/knowledge based on learning pathways. Related yet distinct communities of practice can be associated as a ‘constellation of practice.’ Relationships between communities of practice within a constellation are mediated by ‘boundary objects’: shared aspects, places, things, etc. which reinforce relationships between places and people both in a physical sense and in a relational one (Gosselain 2016; Wenger 1998, pp. 105, 122–133).

The chaînes opératoires for each site indicate that Kingoyi and Kindangakanzi shared metallurgical principles: crucible smelting of carbonate ores without fluxes in reused domestic pottery. These commonalities were predicated on shared knowledge/learning and processes but occurred in spaces both geographically and socially distant from each other. We reconstruct, then, a constellation of practice made up of the discrete communities of practice of producers of Moubiri-type and Kindangakanzi-type pottery mediated by the mining landscape as the boundary object. In other words, sharing a space, like a copper mine, encouraged communities to share ideas and technologies, like crucible smelting, and thus form a constellation of practice around metallurgy. Unfortunately, the absence of finished metal products obviates comparisons beyond the stage of smelting, e.g. skill and different copperworking chaînes opératoires or aesthetic valency (Kuijpers 2017; Stahl 2013).

Shared raw materials acting as a boundary object for a constellation of practice is evident in the ethnographic record among potters in Niger and in the Lake Titicaca basin (Corniquet 2011; Roddick 2016). Groups of potters from different villages with their own traditions came together at communal clay pits where ideas and techniques (e.g. paste recipes) were shared. These were then taken up by the various communities of practice in contextually appropriate ways. Knowledge sharing about technical aspects of metallurgy such as fluxes and tuyères among second millennium ironworking communities in western Uganda may have been similarly facilitated by the location of furnaces near roads, encouraging interaction between different groups (Iles 2018).

In the fifteenth–seventeenth century Niari, such interaction is suggested by the presence of Kindangakanzi-type crucibles around Mindouli, and in particular may have crystallised at Mfouati, where both types of pottery are attested at sites around the mines. The relationship between these pottery communities of practice and ethnic/linguistic groups is unknown, but connectivity within the Niari Basin would have fostered interaction between communities. Historical sources mention the Niari as one of the main trade axes between the Loango coast and the Malebo Pool from the seventeenth century onwards (Martin 1972) and archaeological evidence suggests copper was traded along the Niari from at least the fourteenth century (Denbow 2014; Nikis 2018b). By the mid-seventeenth century, Loango was estimated to be exporting some 70,000 pounds per year (Herbert 1984, pp. 141–142; Martin 1972, pp. 41–44).

This knowledge centred on the reuse of decorated domestic pottery as crucibles, built on an understanding of the pottery’s suitability for use in metallurgy. The metallurgical practice around these crucibles was also similar, with short smelting reactions of high-grade ore in small, portable vessels. While not ideal for retrieving as much metal as theoretically possible, such a process was clearly sufficient for the purposes of the smelters and in addition to not requiring the sourcing of additional materials as flux may also have been suited to a lesser consumption of fuel. The landscape of the Niari is grassy and shrubby savannah, with few trees, meaning that access to fuel was likely a concern (Koechlin 1961, pp. 131–161; Schwartz et al. 2000). This may have been particularly acute during the period in question, as the sixteenth–nineteenth century in central Africa may have been more arid (Hubau 2013; Verschuren and Charman 2008). At Kindangakanzi, however, the furnace, possibly used for different ores and/or in a different phase, suggests variation in practice or organisation of production. Around Mindouli, furnaces at sites other than Kingoyi, e.g. Moubiri, suggest similar variation.

Knowledge sharing around metallurgy may also have been a function of the temporality of the mining and smelting processes. In the first half of the seventeenth century, Dapper records that Vili caravans came up to the Niari from the coastal Kingdom of Loango in the wet season (September-May), implying regularity and cyclicality (Dapper 1676; Herbert 1984, p. 142). These movements may have exposed the Vili to either new clay sources or to new groups of local people with whom they could trade for pottery to use in smelting. On the other hand, trade with Loango need not necessitate mining and smelting by the Vili themselves. Oral history conducted within the Niari indicated that in the nineteenth century, local inhabitants were in control of the mining and smelting (Volavka 1998, pp. 184–187). The situation may have been the same in prior periods, as Dapper records that it was the locals who smelted the different ores together, suggesting that any constellation of practice around mines may have included both locals and outsiders.

This sharing of resources and practices need not be not all-encompassing, however, as the pottery and technical ceramics in the Niari demonstrate. The absence of pottery subgroups, either decorative or compositional, makes it difficult to assess choices within communities of practice, and without a better understanding of chronological typology within the fifteenth–seventeenth century, it is currently impossible to track evidence for transmission of pottery making knowledge, e.g. apprenticeship (Wendrich 2013). However, variation in the use of technical ceramics is pronounced both within the fifteenth–seventeenth century and between the various periods of copper exploitation. While the reuse of domestic pottery as crucibles is paralleled elsewhere in sub-Saharan Africa, e.g. Mapungubwe (Chirikure et al. 2015) and Marothodi (Hall et al. 2006), the technological choice involved in the reuse of decorated domestic pottery as crucibles in the fifteenth–seventeenth century Niari is significant given that:

1. The reuse of domestic pottery as crucibles is a commonality between the two communities of practice.
2. Both communities of practice used these crucibles for smelting, as opposed to melting or refining, suggesting similar spheres of metallurgical knowledge.
3. Separate clay recipes, both refractory, were used by each community, despite the availability of both clays.
4. At Kindangakanzi, purpose-made technical ceramics (tuyères/furnace) were made in both fabrics.
5. Thirteenth–fifteenth century copper production associated with Misenga-type pottery and nineteenth century production around Boko-Songho and Mindouli both featured specialised crucibles and were all heated from the outside.
6. Talc-based fabrics were used in the thirteenth–fifteenth century for Misenga-type pottery but not for Misenga-type crucibles.

The selection of one clay or the other, then, was not simply a matter of geological availability. Instead, it reflected social, historical, and cultural processes of separate potting communities of practice: producers of Moubiri-type vessels, whose copper production was centred around Mindouli at the east and producers of Kindangakanzi-type around Boko-Songho at the west. Both groups used relatively highly refractory clays, enabling the reuse of the vessels as crucibles, remarkably thin-walled compared to typical smelting furnaces or crucibles. When knowledge about crucible smelting was shared, then, it occurred with already complete pottery. The mining and smelting locales may have acted as boundary objects, but only for sharing knowledge about the processes of metallurgy.

It is also significant that the mineralogical and chemical data are consistent with both types of pottery being made within the Niari, a fact that was not previously clear. In terms of trace elements, talc-based tuyères at Kindangakanzi generally group with Kindangakanzi-type pottery, and quartz-based tuyères at Kingoyi with Moubiri-type pottery. Purpose-made technical ceramics such as tuyères and furnaces are typically made and discarded at the site of use, particularly when not fired prior to use, as in the Niari. While this consistent geological signature is likely to be local to the Niari, the range of such a signature is unclear, particularly as both the quartz-rich Mpioka and talc-rich Schisto-Calcaire Formation extend throughout the Niari and beyond; indeed, talc-rich Misenga-type pottery of the thirteenth–fourteenth centuries found around Mindouli is consistent with fifteenth–seventeenth century Kindangakanzi-type pottery found around Boko-Songho, suggesting that there may be considerable overlap (Fig. 7) and other pottery groups collected south of the Congo River also display talc-related minerals in their fabrics (Tsoupra et al. 2022). Regardless, it seems likely that similar clay sources within the Niari were exploited diachronically, making Kindangakanzi-type pottery essentially a successor to Misenga-type in the use of talc-rich clays.

Given the use of talc-rich clays in Misenga-type pottery, it is interesting that a Misenga-type crucible analysed here was made of quartz-rich clay. In the earlier period of production, then, it appears that talc-based clays were known and used but not necessarily as technical ceramics, although analysis of more samples is necessary for further comment. In the fifteenth–seventeenth century, such clays were used to create magnesia-rich pottery which was then reused as refractory crucibles, a unique technological choice in sub-Saharan African metallurgy. Again, it must be stressed that while its suitability for reuse in metallurgy was surely recognised, it is not clear that the clay was chosen specifically for its refractoriness. Talc-based pottery, in particular vessels with woven decoration, may have been emulative of the texture of raffia cloth, highly valued within West Central Africa generally and the Kongo Kingdom specifically (Cranshof et al. 2018; Vansina 1998).

Within the context of fifteenth–seventeenth century copper metallurgy, it is plausible that an understanding of the refractoriness of Kindangakanzi-type pottery and its suitability for use in metallurgy was predicated on an understanding of its soapy feel. The recognition of refractory crucibles based on colour and texture is attested, for example, in post-Medieval Europe, where desirable Hessian and Bavarian crucibles were traded and even emulated by replicating their sensorial qualities (Martinón-Torres and Rehren 2009). A further possibility is that local potters and smelters in the fifteenth–seventeenth century realised that domestic pottery made from widely available and historically exploited local clays was suitably refractory, obviating the need to create specialised crucibles in this period.

Technical data from copper production evidence from Kingoyi near Mindouli and Kindangakanzi around Boko-Songho has facilitated a metallurgical reconstruction of copper production in the Niari Basin during the fifteenth–seventeenth century. Analysis of crucibles, tuyères, and a furnace has shed light on the materials and technological choices within the chaînes opératoires of the two sites and allowed for the recognition of a metallurgical constellation of practice formed of the discrete Moubiri-type and Kindangakanzi-type potting communities of practice. However, there are still many aspects of copper production in the Niari in the fifteenth–seventeenth century to be explored. Kingoyi and Kindangakanzi are just two of the numerous sites around Mindouli and Boko-Songho and analysis of others would help characterise the spectrum of variability within each region. Finally, now that there is a relative baseline available for the two types of production and shared metallurgical knowledge, the area around Mfouati, where both types occur, is ripe for a more explicit examination of how these communities of practice interacted.

## Supplementary Information

Below is the link to the electronic supplementary material.

Supplementary file1 (PDF 5405 KB)

Supplementary file2 (PDF 74 KB)
