# Supplementary material for: Smelting copper in decorated pottery: communities of practice in the Niari Basin, Republic of the Congo, fifteenth–seventeenth centuries CE
Source: Archaeol Anthropol Sci. 2022 Oct 6;14(11):210. doi: 10.1007/s12520-022-01653-9 (PMC9537208; doi:10.1007/s12520-022-01653-9)
Supplement: Supplementary file 1 — Supplementary file1 (PDF 5405 KB) [file 12520_2022_1653_MOESM1_ESM.pdf]

Supplementary Information

Supporting materials for:

Cordivari, B.W.*, Nikis, N., Martinón-Torres, M. Smelting copper in decorated pottery: communities of practice in the Niari Basin, Republic of the Congo, 15th-17th centuries CE. *Archaeological and Anthropological Sciences*.

*Institute for the Study of the Ancient World, NYU (bwc2354@nyu.edu)

Contents

Contents 1
Site descriptions 2
pXRF methodology 3
Samples analysed via pXRF 4
Certified Reference Materials 7
Comparison of pXRF and SEM-EDS results 8
Ore geology 9
Pottery 11
Tuyères 13
Supplementary BSE images 14
References 16
Catalogue of polished block, FTIR samples 17

Site descriptions

The sites of the 15th-17th century, as elsewhere in the Niari, suffer from extreme erosion. Kingoyi lies to the southwest of Mindouli, among a group of sites on the Plateau des Cataractes (**Fig. 1**). Over 7.75 kg of metallurgical debris was recovered at the site, including an aggregate of slag/soil (Nikis, 2018, vol. 2, table 3.2, p. 54). A fragment of sandstone was perhaps used for crushing ores and/or slags. Radiocarbon dating of charcoal from layer 3, which included the aggregate, yielded a 2σ date of 1465-1636 cal AD.

Kindangakanzi is located among a series of hills northeast of Boko-Songho. The site was poorly stratified but contained an ellipsoidal bowl furnace (**Fig. 2**). Over 2.75 kg of metallurgical debris (slags, tuyères, crucibles, and ores) was recovered (Nikis, 2018, vol. 2, table 17.2, p. 251). Radiocarbon analysis of charcoal from the furnace established a 2σ date of 1457-1627 cal AD. Lead isotope analysis of slag from Kindangakanzi indicated that it is consistent with the lead-rich copper ores from Djenguelé, a mine ca. 2 km from the site (Rademakers et al., 2018).

The crucible assemblages of both sites are highly fragmentary, with sherds only a few cm in size. Around 10-20% of ceramics at Kingoyi are slagged, and 30-60% from the various sondages at Kindangakanzi are slagged (Nikis, 2018, vol. 2, pp. 53, 248). Tuyères are less abundant at Kingoyi than at other sites near Mindouli (15% of metallurgical debris by weight, cf. 75% at Ntominsié); tuyères around Mindouli are similar in dimension, suggesting standardisation (Nikis, 2018, p. 308). Tuyères are common at Kindangakanzi (ca. 60% by weight).

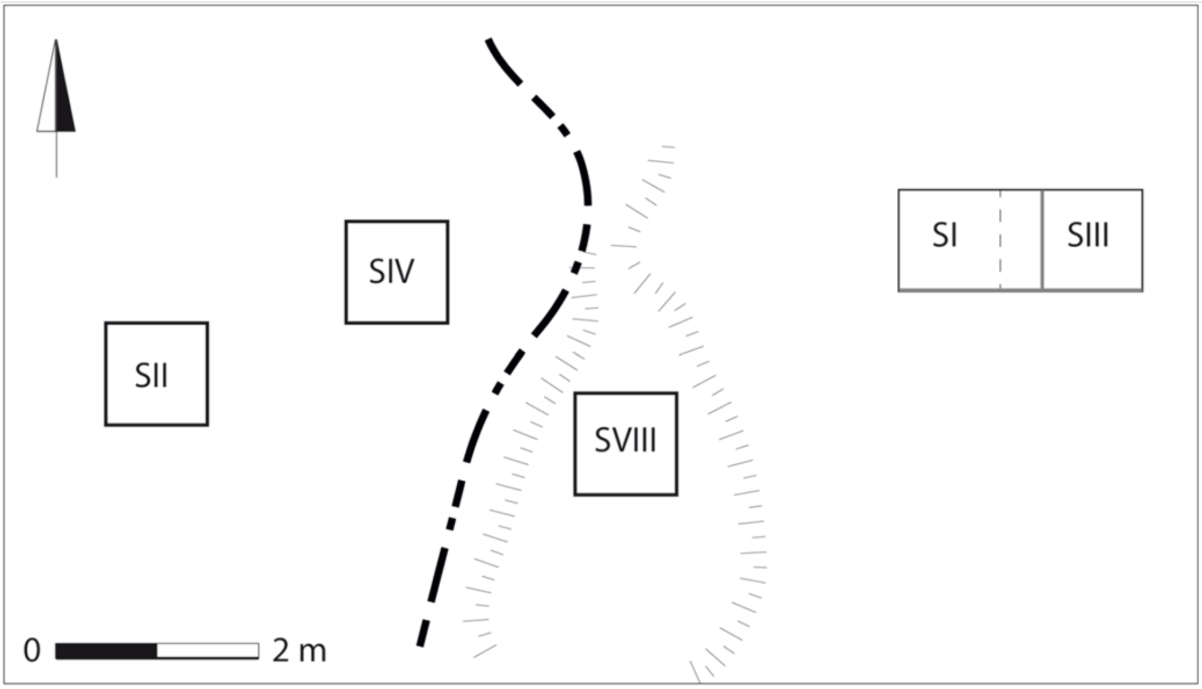

Fig. 1 Location of sondages at Kingoyi

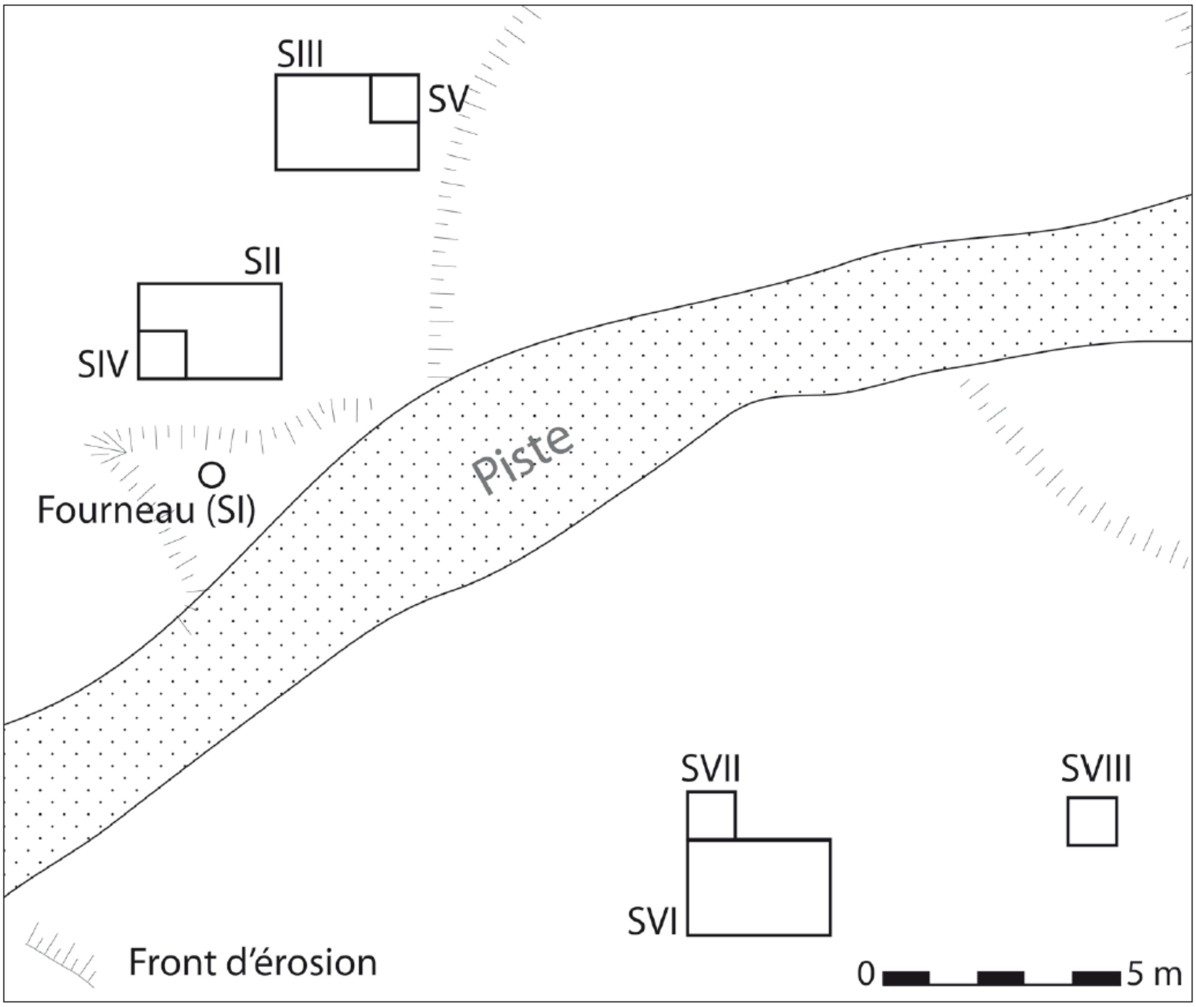

Fig. 2 Location of sondages at Kindangakanzi

Table 1 Geographical coordinates of sites mentioned in the main text. Geospatial data gathered via GPS according to WGS84 projection

Area	*Site*	*E*	*S*
**Mindouli**	Kingoyi	14° 19' 44''	4° 17' 43''
**Boko-Songho**	Kindangakanzi	13°38'00''	4°25'49''

pXRF methodology

Screening pXRF analysis were performed on unprepared surfaces using Olympus Vanta VMR instruments. Two different instruments of the same model and comparable settings were used. For each sample, between 1-3 analyses were conducted per surface (slag, ceramic). Analyses were performed using the 'GeoChem' mode, a fundamental parameters calibration supplemented with empirical optimisation and including two beams: one at 40 kV with an Al filter, and and another one at 10 kV with no filter for lighter elements, with measurement times of 30 s per beam. Beam currents are automatically adjusted by the system for each analysis, ranging 63-78 µA (Table 2). The elements quantified include: Mg, Al, Si,

P, S, K, Ca, Ti, V, Cr, Mn, Fe, Co, Ni, Cu, Zn, As, Se, Rb, Sr, Y, Zr, Nb, Mo, Ag, Cd, Sn, Sb, Ba, W, Au, Hg, Pb, Bi, Th, and U.

We analysed a soil standard using one of the instruments, demonstrating that light elements tend to be underestimated but that overall accuracy is acceptable for the elements used in our discussion (Table 4). Unfortunately, the same standard was not available when the second instrument was employed, but the use of identical settings and the correspondence between groups irrespective of the analytical batch is indicative of consistency in the results. It should be noted, however, that we analysed unprepared surfaces in order to preserve artefact integrity, and therefore analytical uncertainty and error are likely higher than suggested by the analysis of standards. In any case, SEM-EDS of a subset of samples prepared as polished blocks confirmed the chemical patterns and group attributions based on pXRF, which reinforces the validity of our sequential approach to sampling and analysis.

Table 2 pXRF specifications

Serial number	***Model***	***Tube***	***Method***	***Voltage (kV)***	***Current (µA)***	***Filters***
**805557**	Olympus Vanta	Rh	GeoChem	40	63	Al
				10	69	-
**841606**	Olympus Vanta	Rh	GeoChem	40	68	Al
				10	78	-

Samples analysed via pXRF

Table 3 Samples analysed via pXRF in the present study (n = 85), prepared as polished blocks (n = 17) and analysed via FTIR (n = 12). Within FTIR column ‘xx’ for crucibles indicates both ceramic body and slag layer analysed, while ‘x’ for crucibles indicates only ceramic body analysed

Sample	Area	Site	Type	Date	pXRF#	Polished block	FTIR
KNA14_29	Boko-Songho	Kindangakanzi	Crucible	15th-17th c.	805557	CA210025	xx
KNA14_30	Boko-Songho	Kindangakanzi	Crucible	15th-17th c.	805557		
KNA14_31	Boko-Songho	Kindangakanzi	Crucible	15th-17th c.	805557		
KNA14_32	Boko-Songho	Kindangakanzi	Crucible	15th-17th c.	805557		
KNA14_33	Boko-Songho	Kindangakanzi	Crucible	15th-17th c.	805557		
KNA14_34	Boko-Songho	Kindangakanzi	Crucible	15th-17th c.	805557		
KNA14_11	Boko-Songho	Kindangakanzi	Crucible	15th-17th c.	805557	CA210004	
KNA14_9	Boko-Songho	Kindangakanzi	Crucible	15th-17th c.	805557	CA210002	

KNA14_37	Boko-Songho	Kindangakanzi	Furnace wall	15th-17th c.	805557	CA210024	
KNA14_17	Boko-Songho	Kindangakanzi	Pottery	15th-17th c.	805557		
KNA14_18	Boko-Songho	Kindangakanzi	Pottery	15th-17th c.	805557		
KNA14_19	Boko-Songho	Kindangakanzi	Pottery	15th-17th c.	805557		
KNA14_20	Boko-Songho	Kindangakanzi	Pottery	15th-17th c.	805557		
KNA14_21	Boko-Songho	Kindangakanzi	Pottery	15th-17th c.	805557		
KNA14_22	Boko-Songho	Kindangakanzi	Pottery	15th-17th c.	805557		
KNA14_8	Boko-Songho	Kindangakanzi	Pottery	15th-17th c.	805557	CA210001	x
KNA14_36	Boko-Songho	Kindangakanzi	Sample heated clay	15th-17th c.	805557		
KNA14_23	Boko-Songho	Kindangakanzi	Slag	15th-17th c.	805557		
KNA14_24	Boko-Songho	Kindangakanzi	Slag	15th-17th c.	805557		
KNA14_25	Boko-Songho	Kindangakanzi	Slag	15th-17th c.	805557	CA210028	x
KNA14_26	Boko-Songho	Kindangakanzi	Slag	15th-17th c.	805557	CA210029	
KNA14_27	Boko-Songho	Kindangakanzi	Slag	15th-17th c.	805557		
KNA14_28	Boko-Songho	Kindangakanzi	Slag	15th-17th c.	805557		
KNA14_12	Boko-Songho	Kindangakanzi	Tuyere	15th-17th c.	805557		
KNA14_13	Boko-Songho	Kindangakanzi	Tuyere	15th-17th c.	805557		
KNA14_14	Boko-Songho	Kindangakanzi	Tuyere	15th-17th c.	805557		
KNA14_15	Boko-Songho	Kindangakanzi	Tuyere	15th-17th c.	805557		
KNA14_16	Boko-Songho	Kindangakanzi	Tuyere	15th-17th c.	805557		
KNA14_10	Boko-Songho	Kindangakanzi	Tuyere	15th-17th c.	805557	CA210003	
KNA214_1	Boko-Songho	Kindangakanzi 2	Crucible	15th-17th c.	805557		
GPSNN272-5	Boko-Songho	Malembe	Ore		841606		x
GPSNN272-9	Boko-Songho	Malembe	Rock		841606		x
GPSNN113_1	Boko-Songho	Montagne Koka	Pottery	13-14th c.	841606		
GPSNN113_2	Boko-Songho	Montagne Koka	Pottery	13-14th c.	841606		x
GPSNN286_1	Mfouati	Ntadiou-Moubouka	Crucible	15th-17th c.	805557		
GPSNN287_2	Mfouati	Ntadiou-Moubouka	Crucible	15th-17th c.?	805557		
GPSNN286_2	Mfouati	Ntadiou-Moubouka	Tuyere	15th-17th c.?	805557		

MKU3b14_2	Mindouli	Kingoyi	Crucible	15th-17th c.	805557		
MKU3b14_3	Mindouli	Kingoyi	Crucible	15th-17th c.	805557	CA210045	x
MKU3b15_12	Mindouli	Kingoyi	Crucible	15th-17th c.	805557		
MKU3b15_13	Mindouli	Kingoyi	Crucible	15th-17th c.	805557	CA210031	xx
MKU3b15_7	Mindouli	Kingoyi	Crucible	15th-17th c.	805557		
MKU3b15_9	Mindouli	Kingoyi	Crucible	15th-17th c.	805557		
MKU3b15_5	Mindouli	Kingoyi	Crucible	15th-17th c.	805557	CA210008	
MKU3b14_7	Mindouli	Kingoyi	Ore	15th-17th c.	805557		x
MKU3b14_9	Mindouli	Kingoyi	Ore	15th-17th c.	805557	CA210030	x
MKU3b14_1	Mindouli	Kingoyi	Pottery	15th-17th c.	805557		
MKU3b14_4	Mindouli	Kingoyi	Pottery	15th-17th c.	805557		
MKU3b15_10	Mindouli	Kingoyi	Pottery	15th-17th c.	805557		
MKU3b15_11	Mindouli	Kingoyi	Pottery	15th-17th c.	805557		
MKU3b15_6	Mindouli	Kingoyi	Pottery	15th-17th c.	805557		
MKU3b15_8	Mindouli	Kingoyi	Pottery	15th-17th c.	805557		
MKU3b15_3	Mindouli	Kingoyi	Pottery	15th-17th c.	805557		
MKU3b15_4	Mindouli	Kingoyi	Pottery	15th-17th c.	805557	CA210007	x
MKU3b15_21	Mindouli	Kingoyi	Slag	15th-17th c.	805557		
MKU3b14_5	Mindouli	Kingoyi	Slag	15th-17th c.	805557	CA210026	x
MKU3b14_6	Mindouli	Kingoyi	Slag	15th-17th c.	805557		
MKU3b14_8	Mindouli	Kingoyi	Slag	15th-17th c.	805557	CA210027	
MKU3b15_14	Mindouli	Kingoyi	Slag	15th-17th c.	805557		
MKU3b15_15	Mindouli	Kingoyi	Slag	15th-17th c.	805557		
MKU3b15_16	Mindouli	Kingoyi	Slag	15th-17th c.	805557		
MKU3b15_17	Mindouli	Kingoyi	Slag	15th-17th c.	805557		
MKU3b15_18	Mindouli	Kingoyi	Slag	15th-17th c.	805557		
MKU3b15_19	Mindouli	Kingoyi	Slag	15th-17th c.	805557		
MKU3b15_20	Mindouli	Kingoyi	Slag	15th-17th c.	805557		
MKU3b15_26	Mindouli	Kingoyi	Soil aggregate	15th-17th c.	805557		

MKU3b14_10	Mindouli	Kingoyi	Tuyere	15th-17th c.	805557	
MKU3b14_11	Mindouli	Kingoyi	Tuyere	15th-17th c.	805557	
MKU3b14_12	Mindouli	Kingoyi	Tuyere	15th-17th c.	805557	
MKU3b15_22	Mindouli	Kingoyi	Tuyere	15th-17th c.	805557	
MKU3b15_23	Mindouli	Kingoyi	Tuyere	15th-17th c.	805557	
MKU3b15_24	Mindouli	Kingoyi	Tuyere	15th-17th c.	805557	
MKU3b15_25	Mindouli	Kingoyi	Tuyere	15th-17th c.	805557	
MKU3b15_2	Mindouli	Kingoyi	Tuyere	15th-17th c.	805557	CA210005
KIS14_7	Mindouli	Kisaba	Pottery	13-15th c.	841606	
MKU3-14_5	Mindouli	Makuti 3	Pottery	13-15th c.	841606	
MKU3-14_4	Mindouli	Makuti 3	Tuyere	13-15th c.	841606	
ULA_14_3	Mindouli	Mpula	Crucible	15th-17th c.	805557	
ULA_14_6	Mindouli	Mpula	Crucible	15th-17th c.	805557	
ULA_14_4	Mindouli	Mpula	Pottery	15th-17th c.	805557	
NTM15_7	Mindouli	Ntominsie	Crucible	15th-17th c.	805557	
NTM15_9	Mindouli	Ntominsie	Crucible	15th-17th c.	805557	
NTM15_8	Mindouli	Ntominsie	Tuyere	15th-17th c.	805557	
GPSNN332_1	Mindouli	Ntomissengué	Crucible	15th-17th c.	805557	
GPSNN331_1	Mindouli	Ntompula	Crucible	13th-15th c.	841606	

Certified Reference Materials

Table 4 SEM-EDS analysis of USGS basalt reference standard BIR-1G. All values wt%

BIR-1G	Na_2O	MgO	Al_2O_3	SiO_2	P_2O_5	K_2O	CaO	TiO_2	FeO
spectrum 1	1.8	9.6	15.6	47.7	0.0	0.0	13.4	1.1	10.9
spectrum 2	1.7	9.5	15.5	47.8	0.0	0.1	13.4	1.0	11.0
spectrum 3	1.8	9.6	15.6	47.6	0.0	0.0	13.3	1.0	11.1
spectrum 4	1.7	9.7	15.5	48.1	0.0	0.1	13.4	1.0	10.6
spectrum 5	1.8	9.5	15.5	47.6	0.0	0.0	13.5	1.0	11.0
average	*1.7*	*9.6*	*15.5*	*47.8*	*0.0*	*0.0*	*13.4*	*1.0*	*10.9*
certified	1.8	9.7	15.5	48.0	0.0	0.0	13.3	1.0	10.4
offset	-0.1	-0.1	0.0	-0.2	0.0	0.0	0.1	0.1	0.5
SD	0.0	0.1	0.0	0.2	0.0	0.0	0.1	0.0	0.2
CV	1.9	0.7	0.1	0.3	200.0	22.4	0.6	2.5	1.5

Table 5: pXRF analysis of powdered soil reference material NIST 2711a using pXRF instrument 805557.

	Mg	Al	Si	P	K	Ca	Ti	V	Cr	Mn
	%	%	%	ppm	%	%	%	ppm	ppm	ppm
analysed	0.86	5.54	22.7	652	2.13	2.21	0.297	<110	80	583
certified	1.07	6.72	31.4	842	2.53	2.42	0.317	81	52	675

	Fe	Ni	Cu	Zn	As	Sr	Cd	Sb	Ba	Pb
	%	ppm	ppm	ppm	ppm	ppm	ppm	ppm	ppm	%
analysed	2.76	28	151	443	115	226	74	32	817	0.137
certified	2.82	22	140	414	107	242	54	24	730	0.140

Comparison of pXRF and SEM-EDS results

Exact correspondence between pXRF and SEM-EDS results cannot be expected, given the different sampling areas, but comparison of both datasets shows broad consistency. (**Fig. 3**). With the exception of Al_2O_3 in metallurgical slag, in general pXRF values are slightly lower, probably because of the higher porosity and irregularity of the surfaces leading to lower analytical totals.

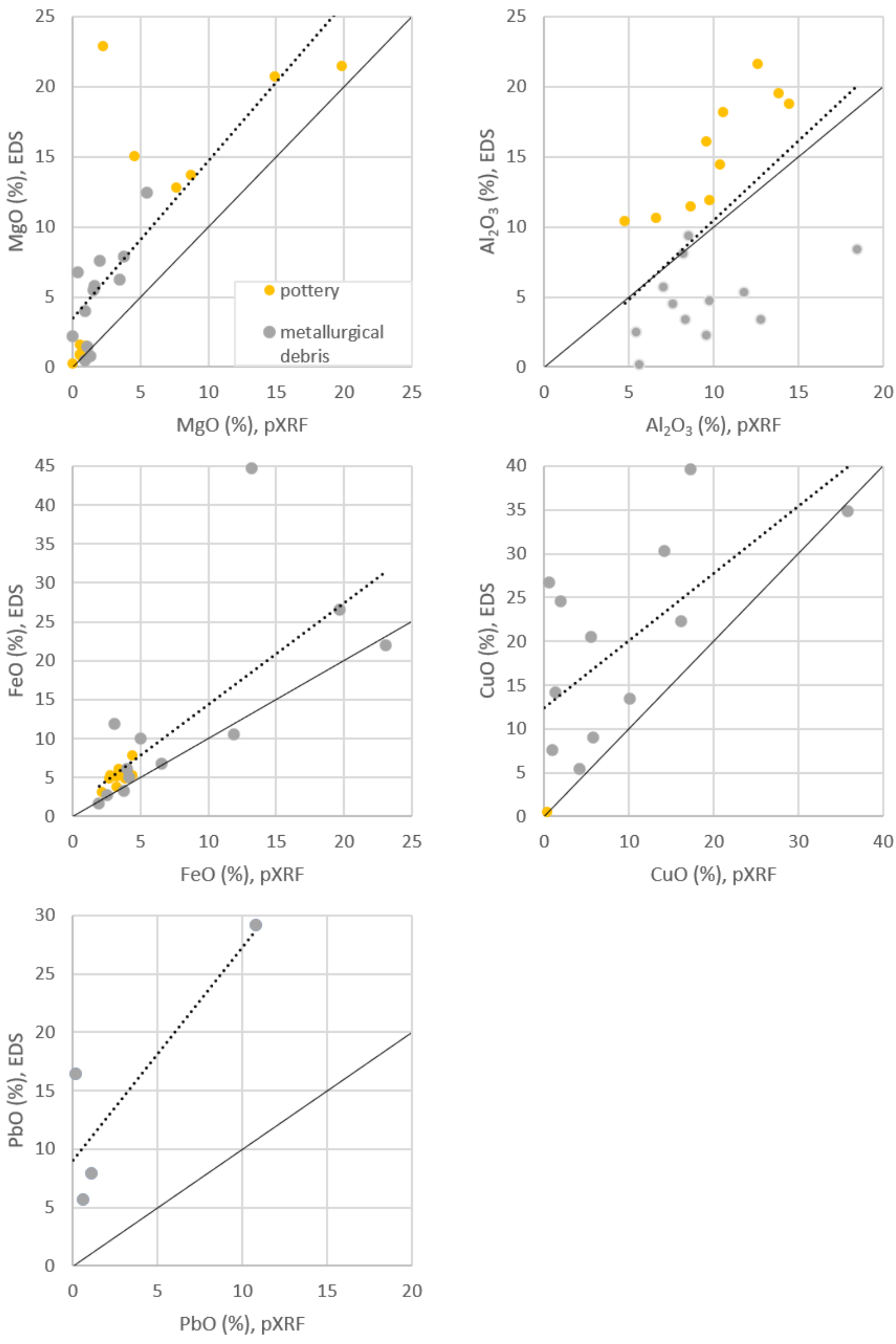

Fig. 3 Comparison of pXRF vs SEM-EDS results for select indicative elements. Dotted line is measured line of fit for all samples, solid line is expected line of y = x

Ore geology

Three samples of ore were analysed, two from Kingoyi and one from Malembe around Boko-Songho (see SI Catalogue). The fragments are <4 cm in maximum dimension. GPSNN272-5 from Malembe contains malachite intergrown with talc and calcite/dolomite. The ores from Kingoyi are not malachite. MKU3b14_7 is principally dioptase. MKU3b14_9 is principally

goethite, found in gossans and thus typical of weathered copper mineralisations, interlaced with veins of dioptase (**Fig. 4**). This composition is reflected in high FeO in the SEM-EDS bulk data presented in the main text (**Table 5**). The presence of iron-rich ores at Kingoyi is noteworthy, as iron concentrations in metallurgical debris are low.

Previously published chemical data indicated minor amounts of manganese (<2000 ppm) in Niari ores, elevated concentrations of silver (<3170 ppm) and arsenic (<6100 ppm) around Mindouli, in particular, and high lead values (<33%), including complex malachite-galena ores, around Boko-Songho (Rademakers et al., 2018). The ores examined here contain manganese, but are broadly similar in terms of silver, arsenic, and lead, likely due to the limitations of the pXRF screening approach.

Mineralisation in the Niari occurs within cracks of the clayey/dolomitic limestones of the Lukala Formation (Schisto-Calcaire Subgroup), and at the fault between the Lukala Formation and the quartzites/siltstones of the Mpioka Subgroup (Cailteux et al., 2015; Nikis and De Putter, 2015). These mineralisations are a complex suite of copper, lead, and zinc formed from hydrothermal alteration (Buffet et al., 1987; Koud, 1987). Supergene ores include vein-filling carbonates, principally malachite, intergrown with talc around Boko-Songho, as with GPSNN272_5 (Bigotte, 1955; Nikis and De Putter, 2016, 2015; Rademakers et al., 2018, OSI; Scolari, 1964). Trace amounts of lead and zinc exist in the ores around Boko-Songho, including complex malachite-galena ores at Djenguelé near Kindangakanzi (Rademakers et al., 2018). The main gangue components, then, are a combination of quartz, dolomitised calcite, and talc, as in the studied samples.

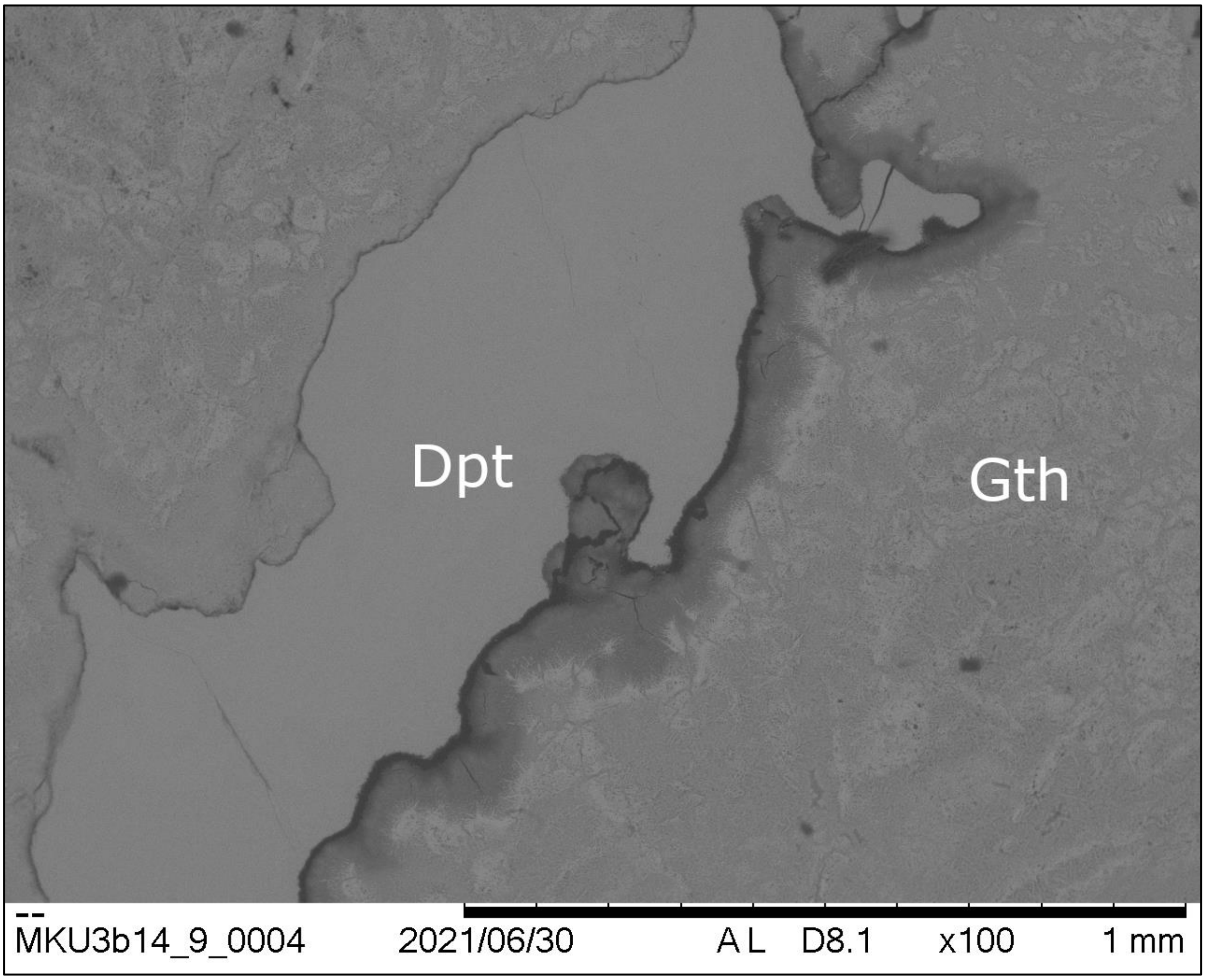

Fig. 4 BSE image of ore MKU3b14_9 showing vein of dioptase (Dpt) in matrix of goethite (Gth)

Pottery

The difference between the kaolinitic Moubiri-type pottery and the smectitic/saponitic Kindangakanzi-type is evident in the FTIR spectra of MKU3b15_13 (ceramic) and KNA14_8 (see SI Catalogue).

Some Kindangakanzi-type samples (KNA14_9, KNA14_29) display angular talc/saponite down to < 10 μm in KNA14_9 and KNA14_29 suggesting a residual clay, i.e., formed from the weathering of a talc vein (Quinn, 2013, pp. 119–122). On the other hand, other samples (KNA14_8, KNA14_11) display rounded inclusions more typical of secondary clays, i.e., from within a river valley. KNA14_8 contains both distinct talc oolites and a fragment of siltstone, a mixed signature from the two main geological formations: oolitic dolomite (Bangu) and siltstone (Mpioka) (see SI Catalogue). MKU3b14_3, a Kindangakanzi-type crucible found at Kingoyi, is akin in terms of macroscopic appearance, refractoriness, and trace element composition, but also contains apatite (**Fig. 5**).

There are peaks in the FTIR spectra of unslagged sherd KNA14_8 (see SI Catalogue) that appear as talc alters to enstatite as well as the development of a shoulder on the Si-O-Si peak, both of which occur around 800° C (Berna et al., 2007; Liu et al., 2014; Weiner, 2010, p.

305). This estimate is merely indicative that the vessels were not subject to a high pre-firing before their reuse as crucibles.

A comparison of bulk and matrix ceramic composition indicates overall similarity, reflective of the use of unmodified clay or the addition of aplastic inclusions from the same geological formation (**Fig. 6**).

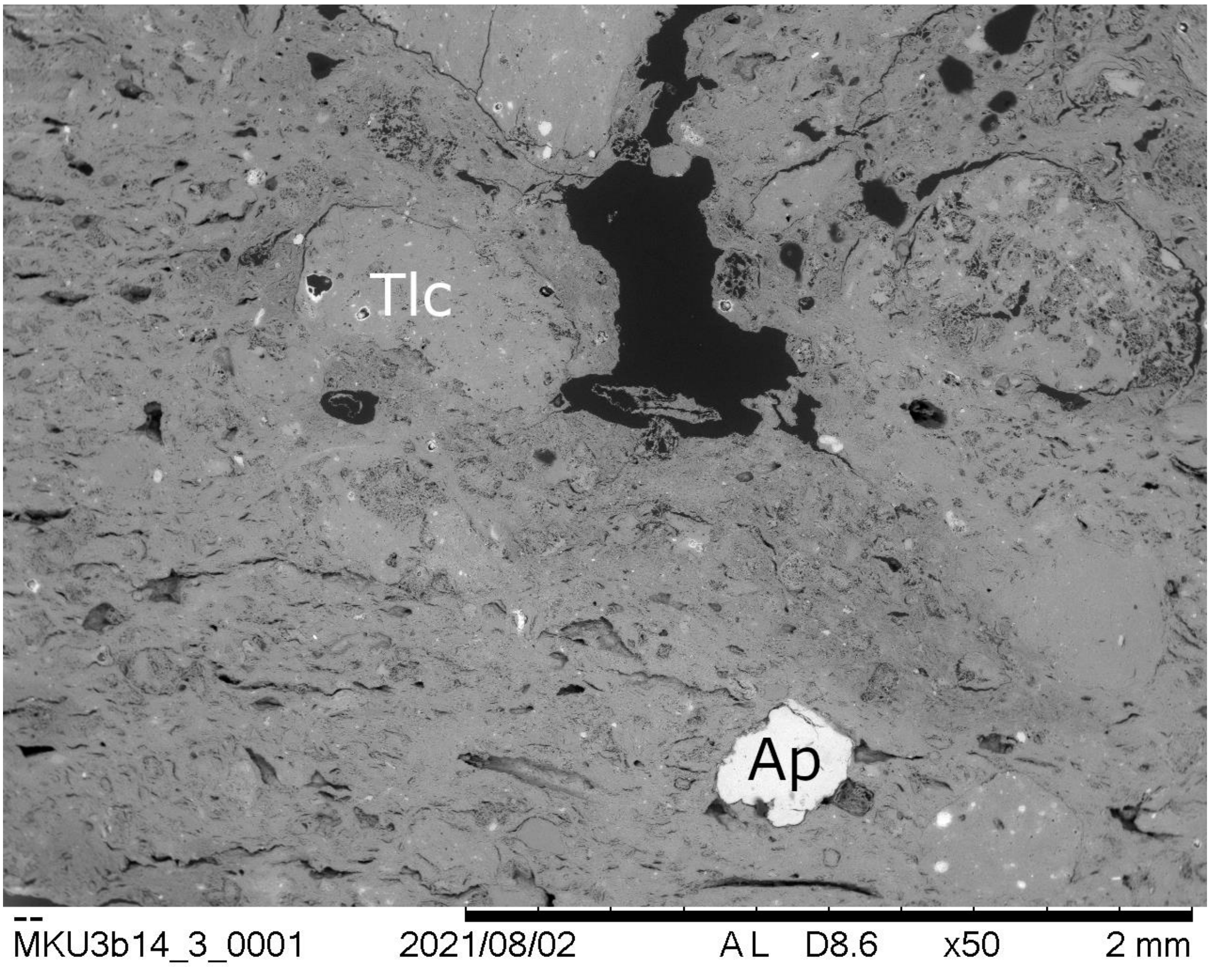

Fig. 5 BSE image of ceramic body of crucible MKU3b14_3 showing bright apatite inclusion (Ap). Note that the talc inclusions (Tlc) are the same colour as the matrix due to their compositional similarity

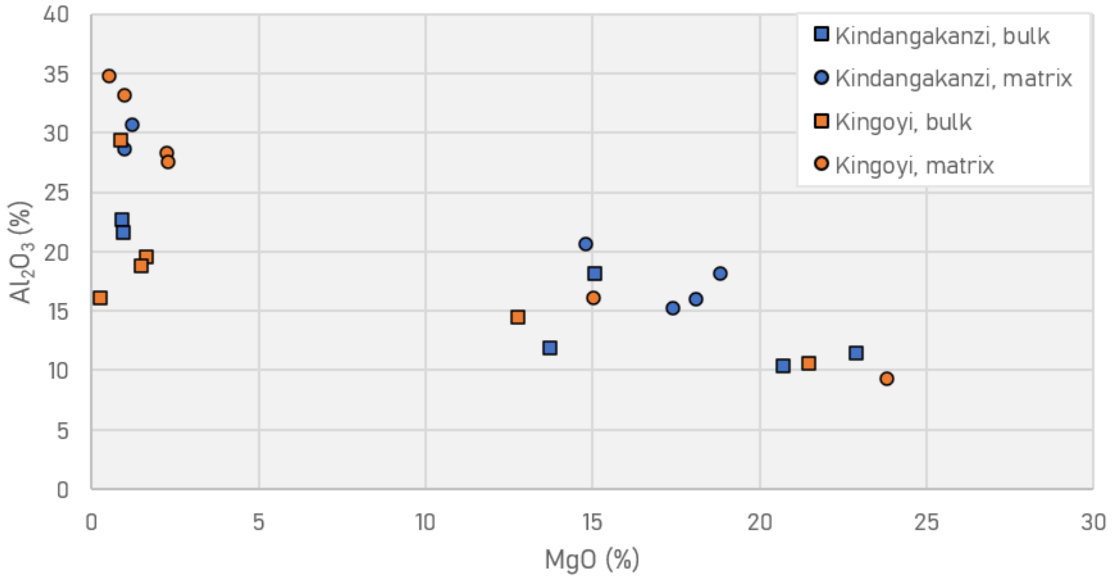

Fig. 6 Plot of Al_2O_3 vs MgO (SEM-EDS data) showing broad similarities in bulk/matrix composition for ceramics from both sites, indicating the use of either unmodified clays or tempering with plastic inclusions from the same geology (i.e., talc, quartz). Note that the alumina-rich ceramics from Kindangakanzi are technical ceramics (tuyère, furnace), while the magnesia-rich Kingoyi samples are decorated Kindangakanzi-type pottery found at Kingoyi

Capacity estimates for a typical Moubiri-type and Kindangakanzi-type vessel are shown in **Fig. 7**.

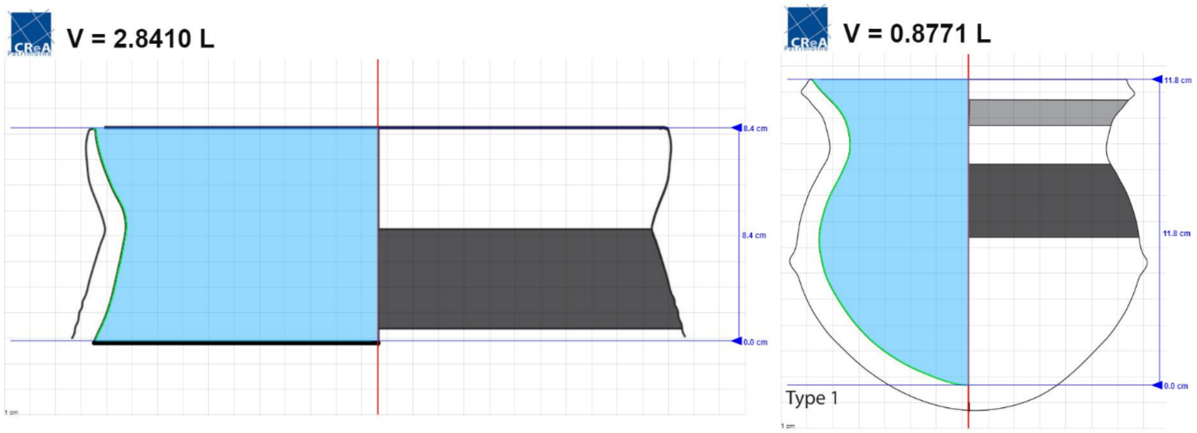

Fig. 7 Capacity estimate using the ULB online calculator (Engels et al., 2009). Left: Incomplete profile of Kindangakanzi-type vessel (Nikis, 2018, fig. 5.21). Right: restored Type 1 Moubiri-type (Nikis, 2018, fig. 5.18). The exact number is less important, especially since the vessels were not necessarily always filled all the way to the brim, but note that even the partial volume of a Kindangakanzi-type vessel is substantially larger than a typical Moubiri one, allowing for a larger charge when used as a crucible

Tuyères

The slag layer on Kingoyi tuyère MKU3b15_2 varies between 1.4-7.0 mm thick and is homogeneous with a smooth, regular surface. The tuyère is clearly linked to copper metallurgy and contains comparable concentrations of copper and iron to other debris. The

slag layer is not particularly enriched in silica or iron relative to the ceramic, indicating that these elements in the slag come from the melting of the quartz-rich ceramic, as visible in section. Prills (<1.2 mm) within the tuyère slag are considerably scarcer than within other slags, and there is trace silver present.

The Kindangakanzi tuyère KNA14_10 is heavily slagged, with a thick, dense black layer on the exterior between 1.2 and 5.5 mm thick. This slag layer contains the highest concentration of iron of residues from the site (22.0% FeO), and the slag layer is enriched in iron relative to the ceramic. The slag also contains large copper prills and 20.5% CuO, confirming its link to copper metallurgy. Notably, the tuyère residue does not contain lead.

Supplementary BSE images

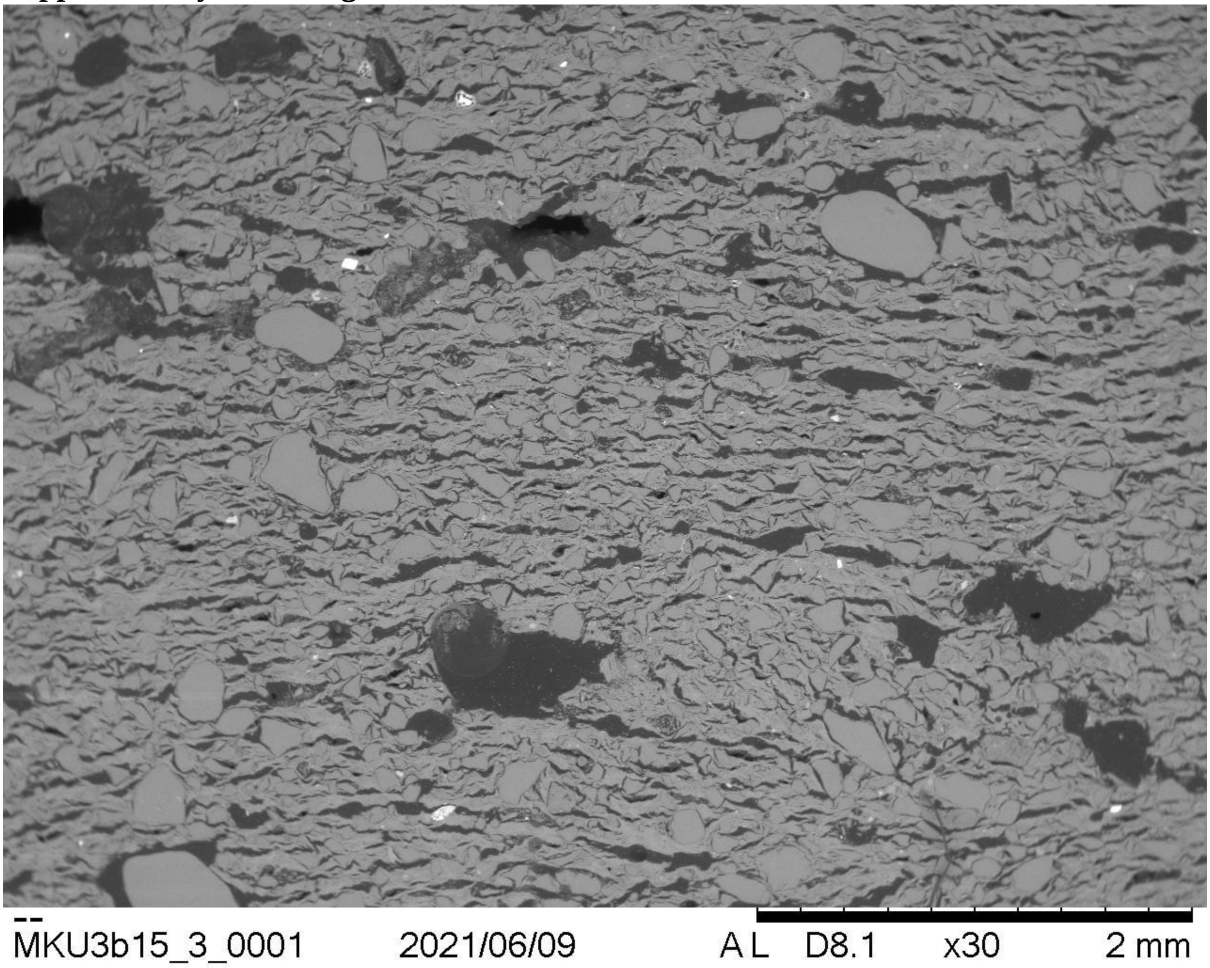

Fig. 8 BSE image of unslagged sherd MKU3b15_3 from Kingoyi showing typical Moubiri-type pottery fabric with quartz inclusions and smaller, brighter iron- and titanium-rich nodules

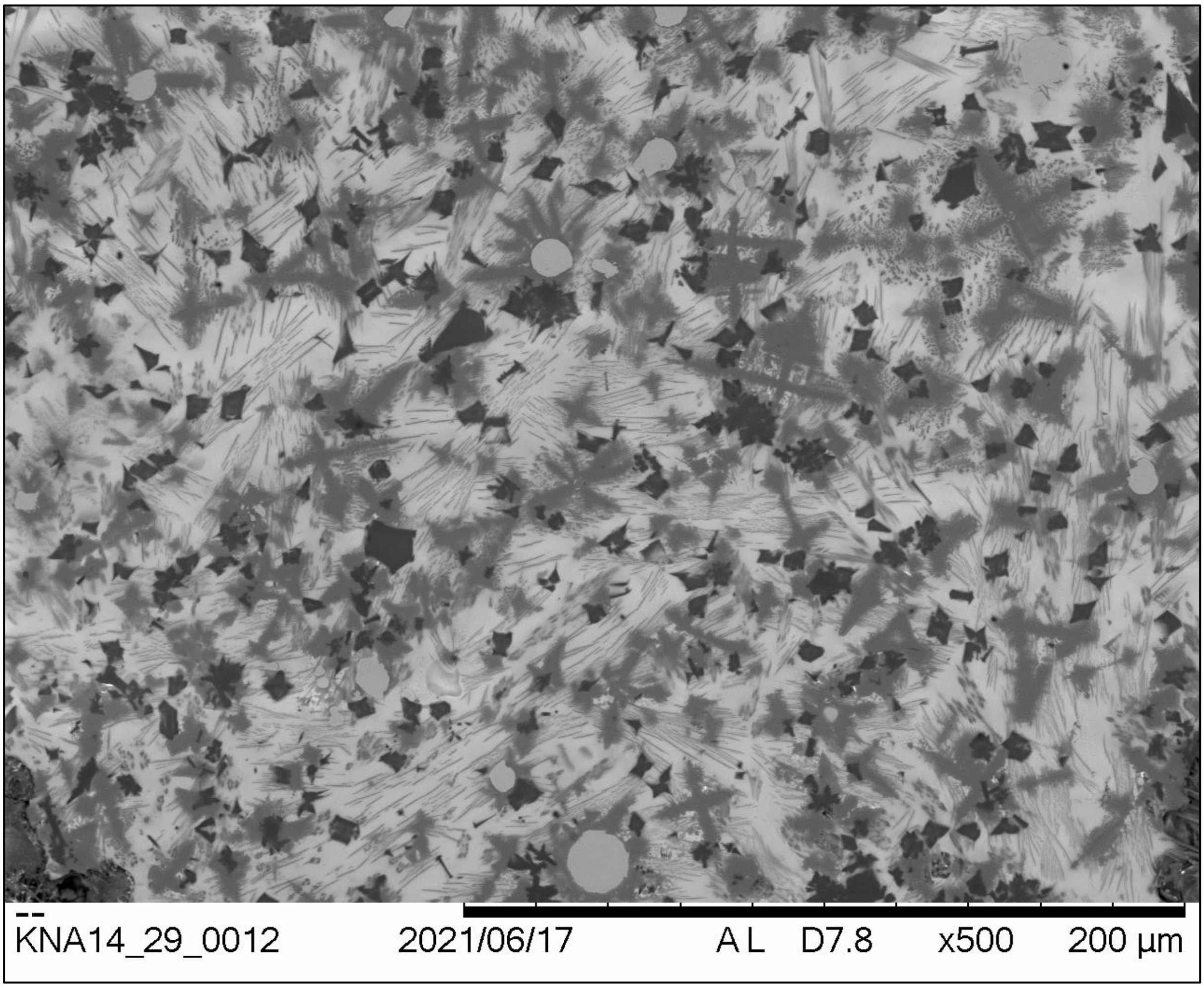

Fig. 9 BSE image of interior slag layer of crucible KNA14_29. Note the bright lead-silicate matrix with spinel (mid-grey) and rounded copper prills (light grey)

References

Berna, F., Behar, A., Shahack-Gross, R., Berg, J., Boaretto, E., Gilboa, A., Sharon, I., Shalev, S., Shilstein, S., Yahalom-Mack, N., 2007. Sediments exposed to high temperatures: reconstructing pyrotechnological processes in Late Bronze and Iron Age Strata at Tel Dor (Israel). J. Archaeol. Sci. 34, 358–373.

Bigotte, G., 1955. Contribution à la géologie du Bassin du Niari. Sédimentologie et métallogénie de la région minière (Ph.D.). Centre d'études nucléaires de Saclay, Gif sur Yvette.

Buffet, G., Amosse, J., Mouzita, D., Giraud, P., 1987. Geochemistry of the M'Passa Pb-Zn deposit (Niari syncline, People's Republic of the Congo). Arguments in favor of a hydrothermal origin. Miner. Deposita 22, 64–77.

Cailteux, J.L.H., Delpomdor, F.R.A., Ngoie Ndobani, J.-P., 2015. The Neoproterozoic West-Congo "Schisto-Calcaire" sedimentary succession from the Bas-Congo region (Democratic Republic of the Congo) in the frame of regional tentative correlations. Geol. Belg. 18, 126–146.

Engels, L., Bavay, L., Tsingarida, A., 2009. Calculating Vessel Capacities: A New Web-Based Solution, in: Tsingarida, Athéna (Ed.), Shapes and Uses of Greek Vases: (7th - 4th Centuries B.C.): Proceedings of the Symposium Held at the Université Libre de Bruxelles 27-29 April 2006, Etudes d'archéologie. Presented at the Symposium, Centre de Recherches en Archéologie et Patrimoine, Bruxelles, pp. 129–134.

Koud, J.M., 1987. Sur l'altération supergène des gisements cuprifères au Congo: Exemple de Mindouli et de Djenguile, in: Séminaire Régional Sur Les Latérites: Sols, Matériaux, Minerais : Sessions 1 et 3. ORSTOM, Paris, pp. 305–320.

Liu, Xiaowen, Liu, Xiaoxu, Hu, Y., 2014. Investigation of the thermal decomposition of talc. Clays Clay Miner. 62, 137–144. https://doi.org/10.1346/CCMN.2014.0620206

Nikis, N., 2018. Archéologie des métallurgies anciennes du cuivre dans le bassin du Niari, République du Congo. Université Libre de Bruxelles, Brussels.

Nikis, N., De Putter, T., 2016. Le cuivre du Niari, une ressource ancienne et prisée: Études géologique et archéologique des mines de uivre-plomb-zinc du bassin du Niari (République du Congo). Sci. Connect. 50, 35–39.

Nikis, N., De Putter, T., 2015. Recherches géo-archéologiques dans les zones cuprifères du bassin du Niari en République du Congo. Nyame Akuma 142–153.

Quinn, P.S., 2013. Ceramic Petrography: The Interpretation of Archaeological Pottery & Related Artefacts in Thin Section. Archaeopress, Oxford.

Rademakers, F.W., Nikis, N., Putter, T.D., Degryse, P., 2018. Copper Production and Trade in the Niari Basin (Republic of Congo) During the 13th to 19th Centuries CE: Chemical and Lead Isotope Characterization. Archaeometry 60, 1251–1270. https://doi.org/10.1111/arcm.12377

Scolari, G., 1964. Etude géologique du bassin du Niari oriental (République du Congo) et de ses minéralisations cuivre-plomb-zinc. Université de Paris, Paris.

Weiner, S., 2010. Microarchaeology: Beyond the Visible Archaeological Record. Cambridge University Press, Cambridge. https://doi.org/10.1017/CBO9780511811210

Catalogue of polished block, FTIR samples

Sample	*Area*	*Site*	*Context*	*Type*	*Date*
KNA14_9	Boko-Songho	Kindangakanzi	SVI 0-10	Crucible	15th-17th c.

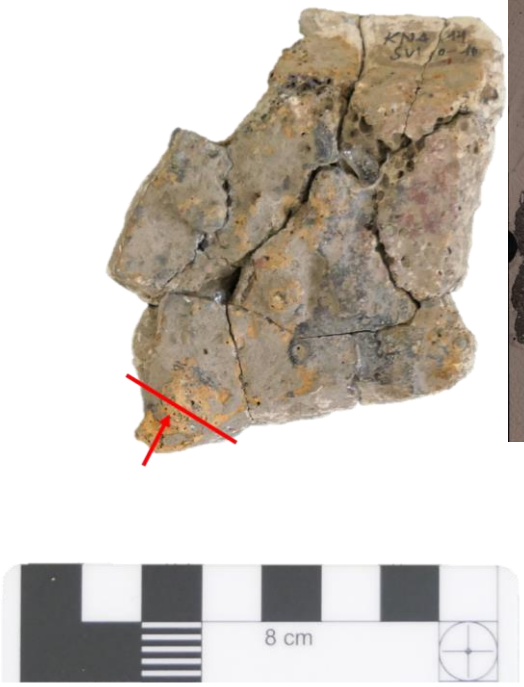

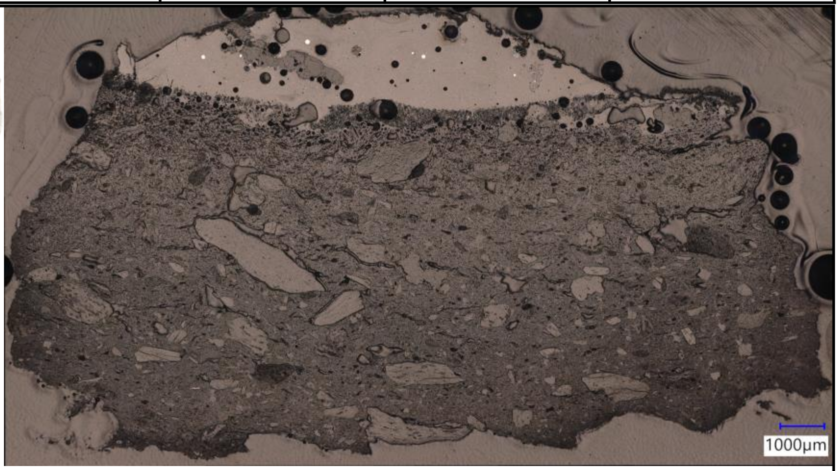

Sample location Cross-section

Sample	*Area*	*Site*	*Context*	*Type*	*Date*
KNA14_11	Boko-Songho	Kindangakanzi	Surface	Crucible	15th-17th c.

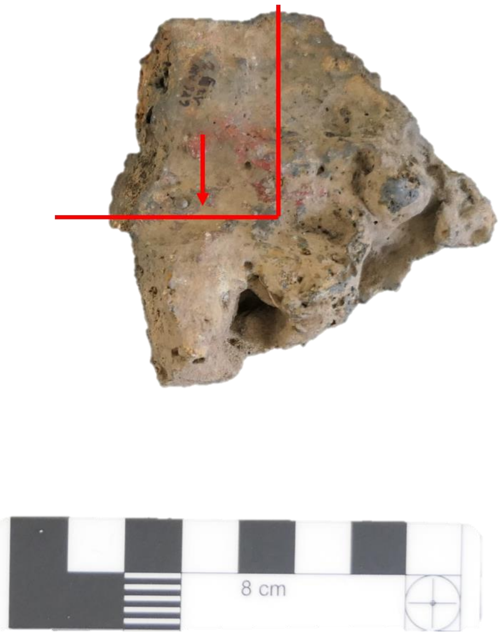

Sample location

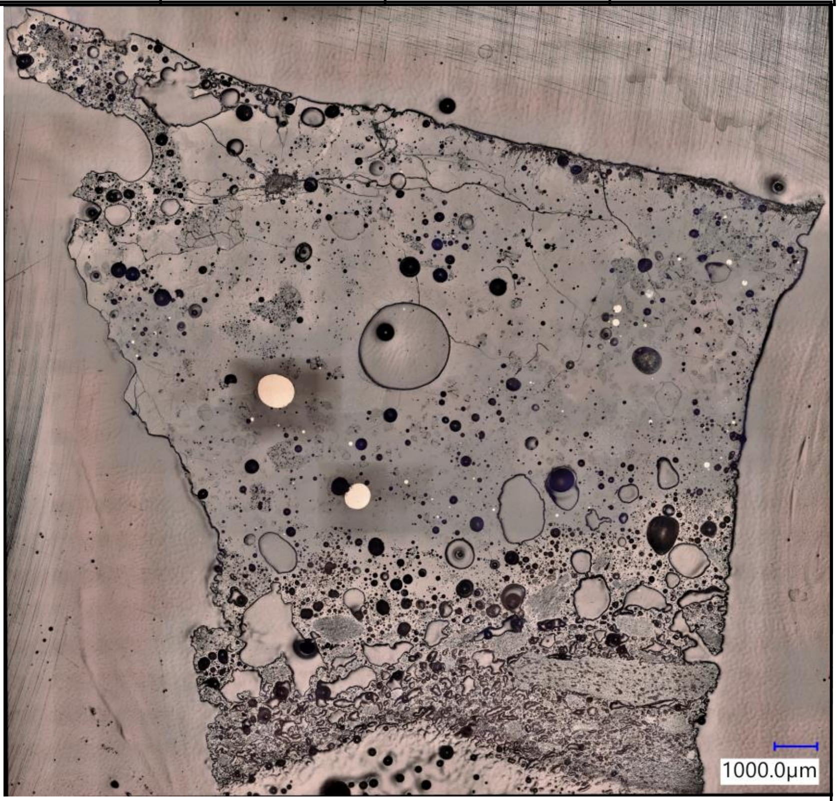

Cross-section

Sample	*Area*	*Site*	*Context*	*Type*	*Date*
KNA14_29	Boko-Songho	Kindangakanzi	SVI 0-10	Crucible	15th-17th c.

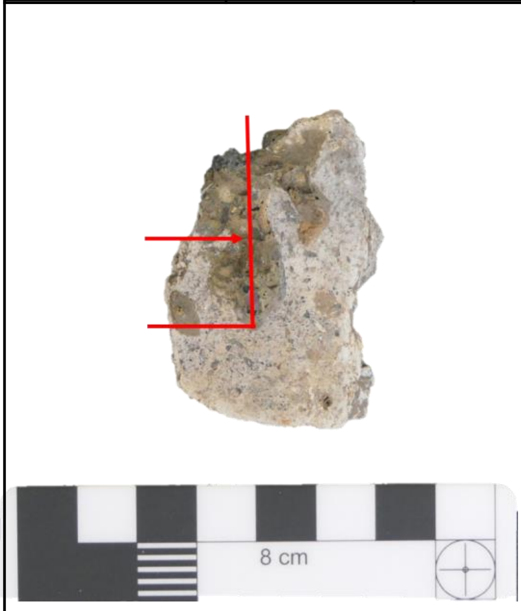

Sample location

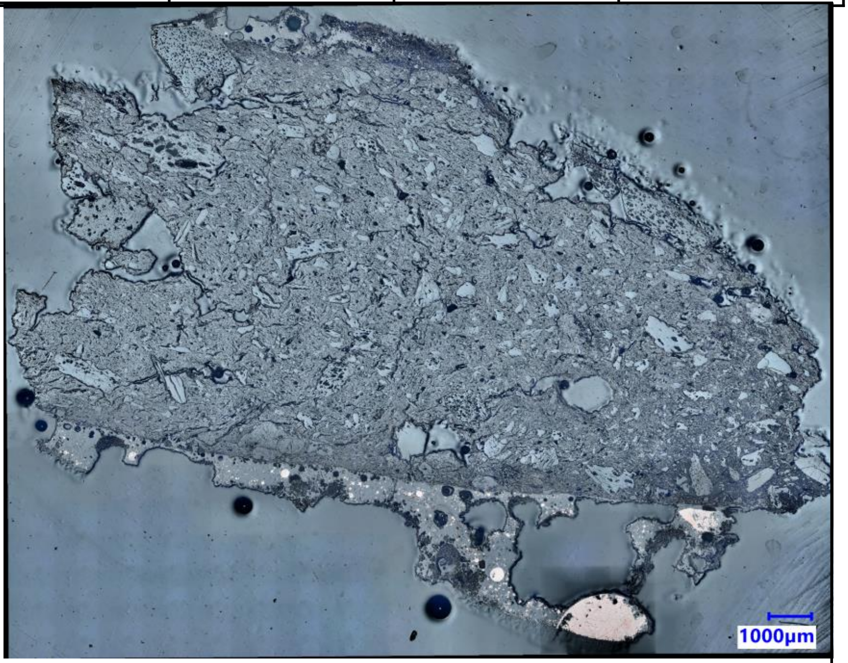

Cross-section

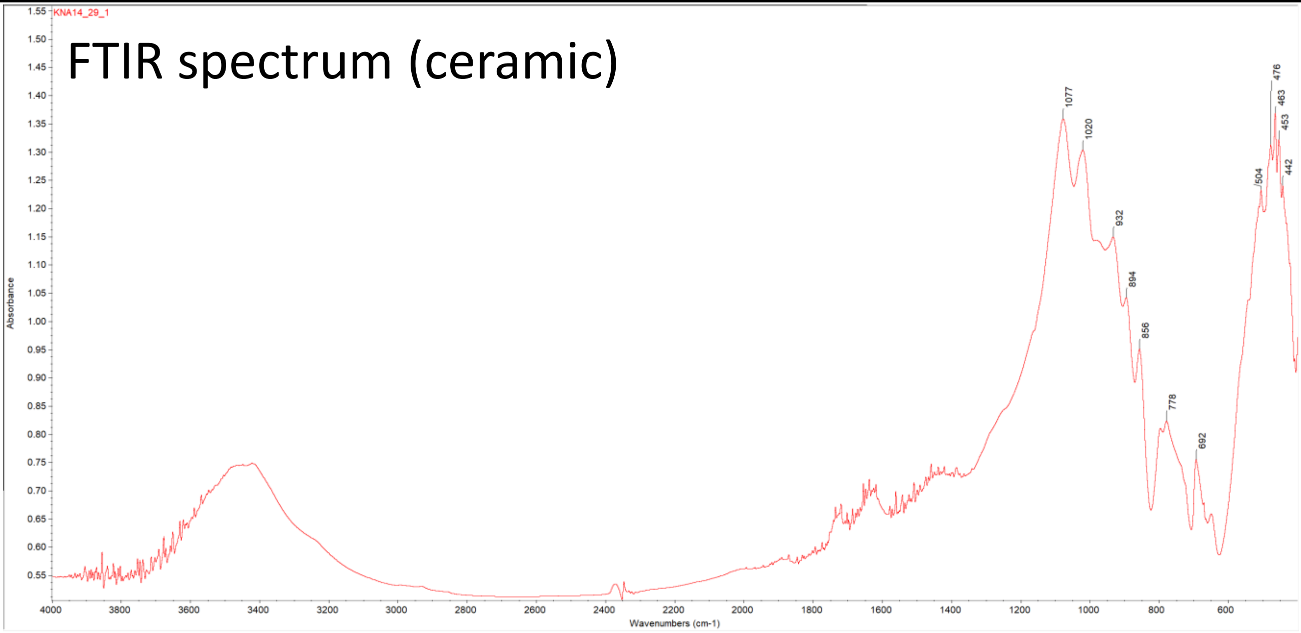

FTIR spectrum (ceramic)

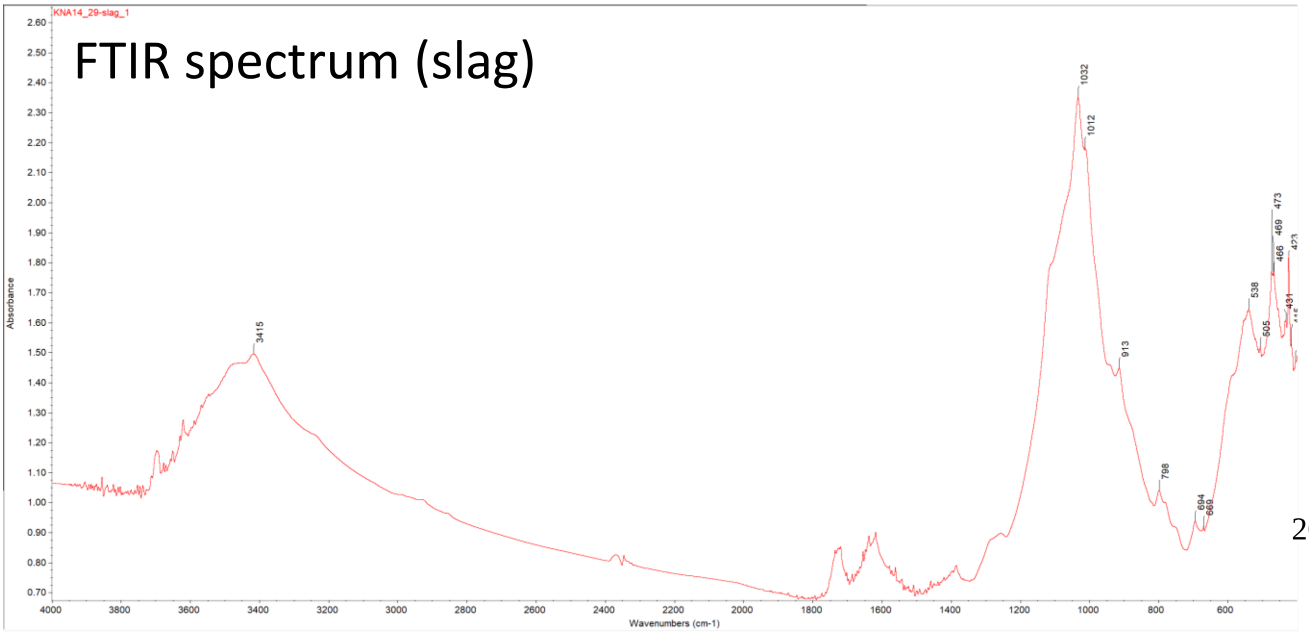

FTIR spectrum (slag)

Sample	*Area*	*Site*	*Context*	*Type*	*Date*
KNA14_37	Boko-Songho	Kindangakanzi	SI Furnace	Furnace wall	15th-17th c.

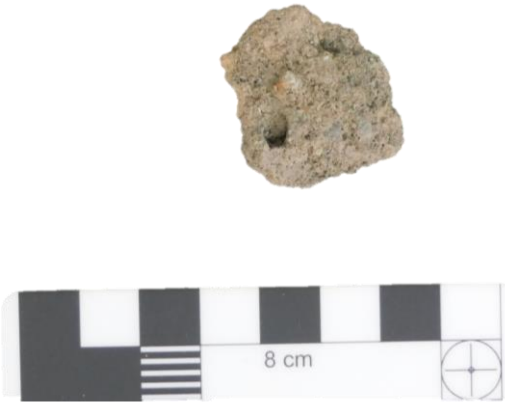

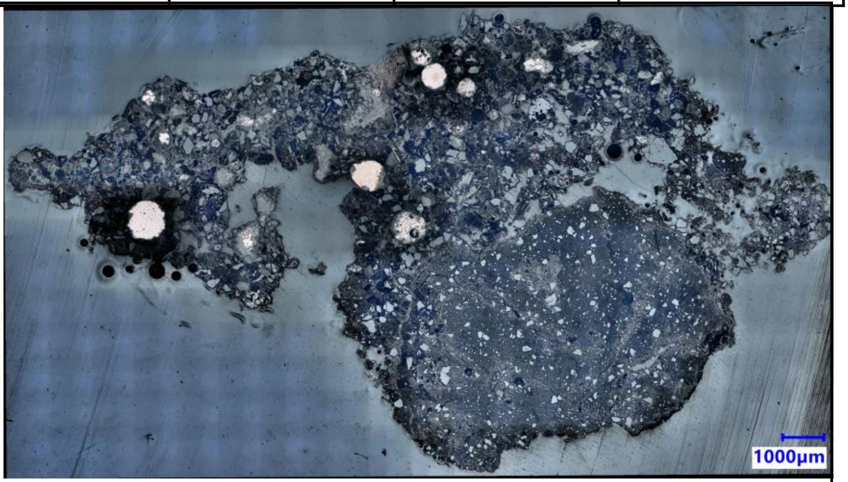

(very friable, exact sample location uncertain)

Cross-section

Sample	*Area*	*Site*	*Context*	*Type*	*Date*
KNA14_8	Boko-Songho	Kindangakanzi	Surface Zone 2	Pottery	15th-17th c.

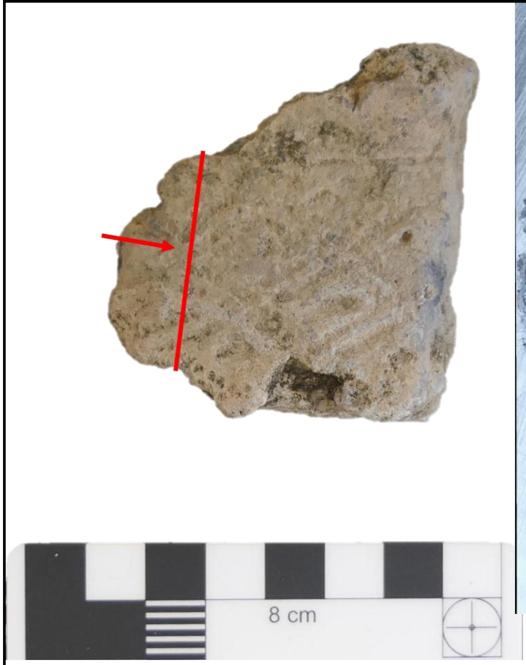

Sample location

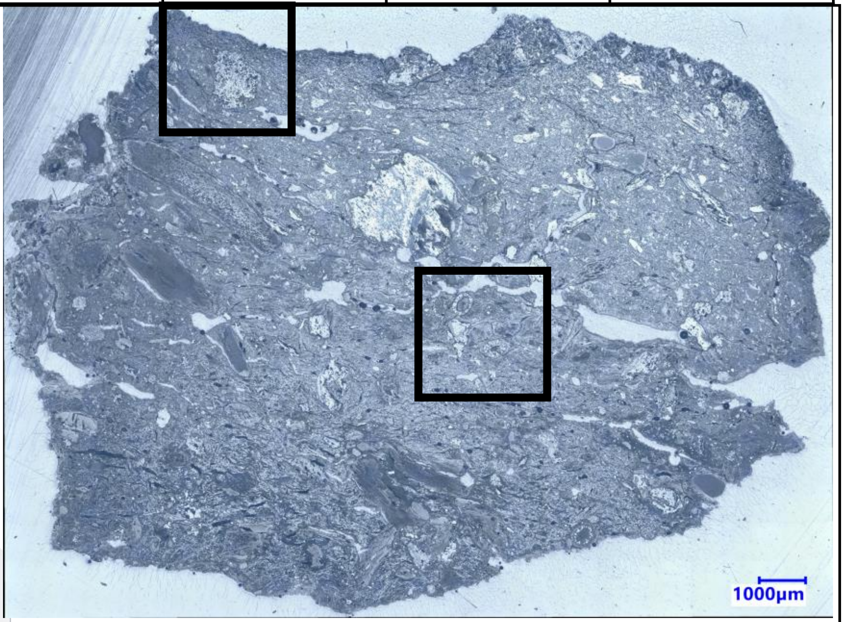

Cross-section (siltstone, talc oolites boxed)

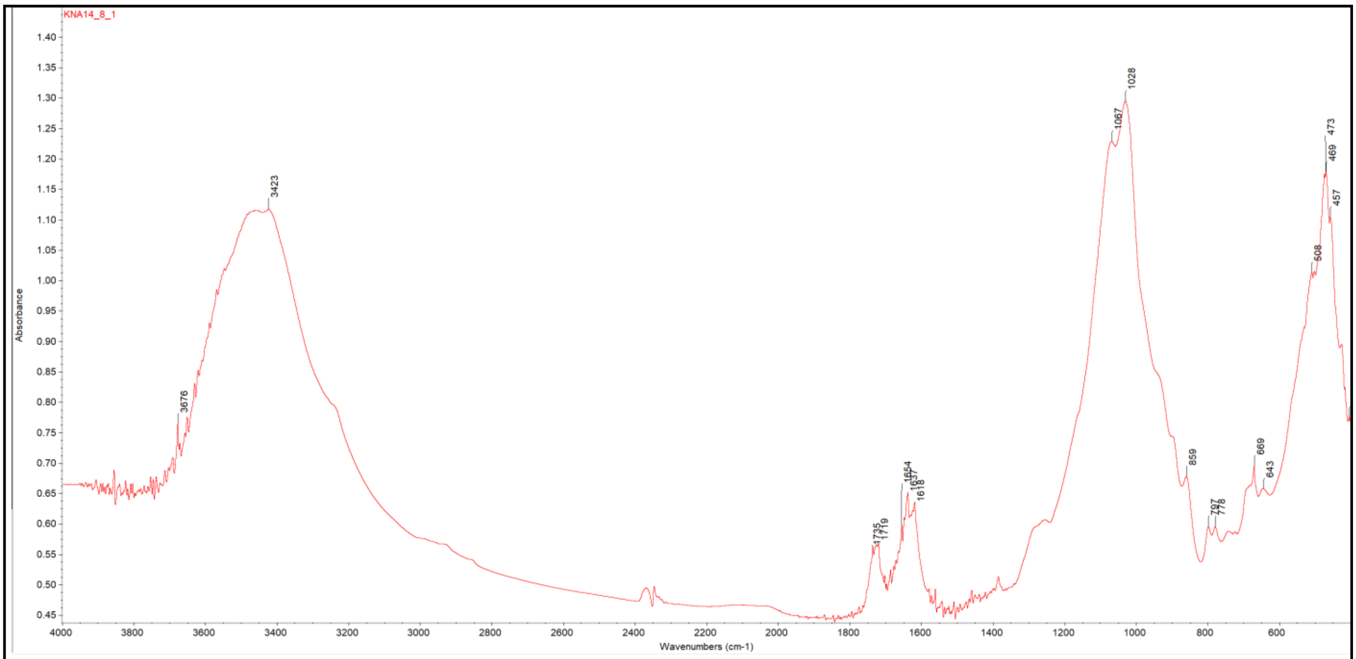

FTIR spectrum

Sample	*Area*	*Site*	*Context*	*Type*	*Date*
KNA14_25	Boko-Songho	Kindangakanzi	SVI 0-10	Slag	15th-17th c.

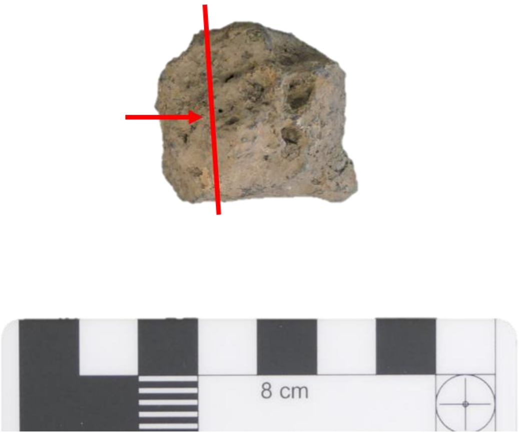

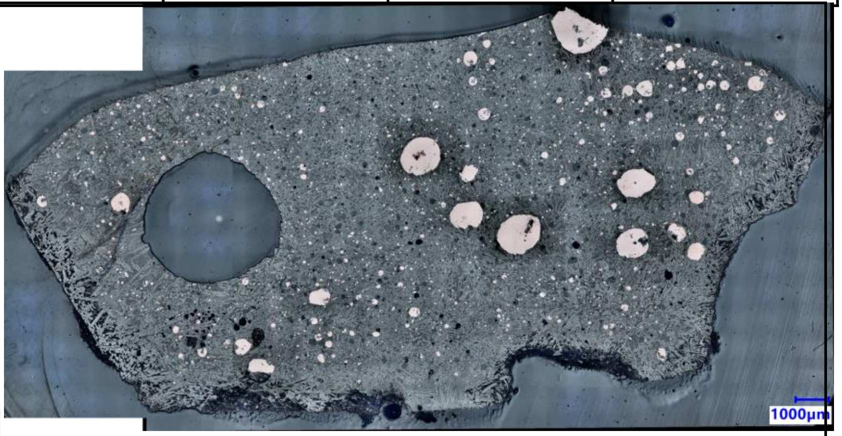

Sample location Cross-section

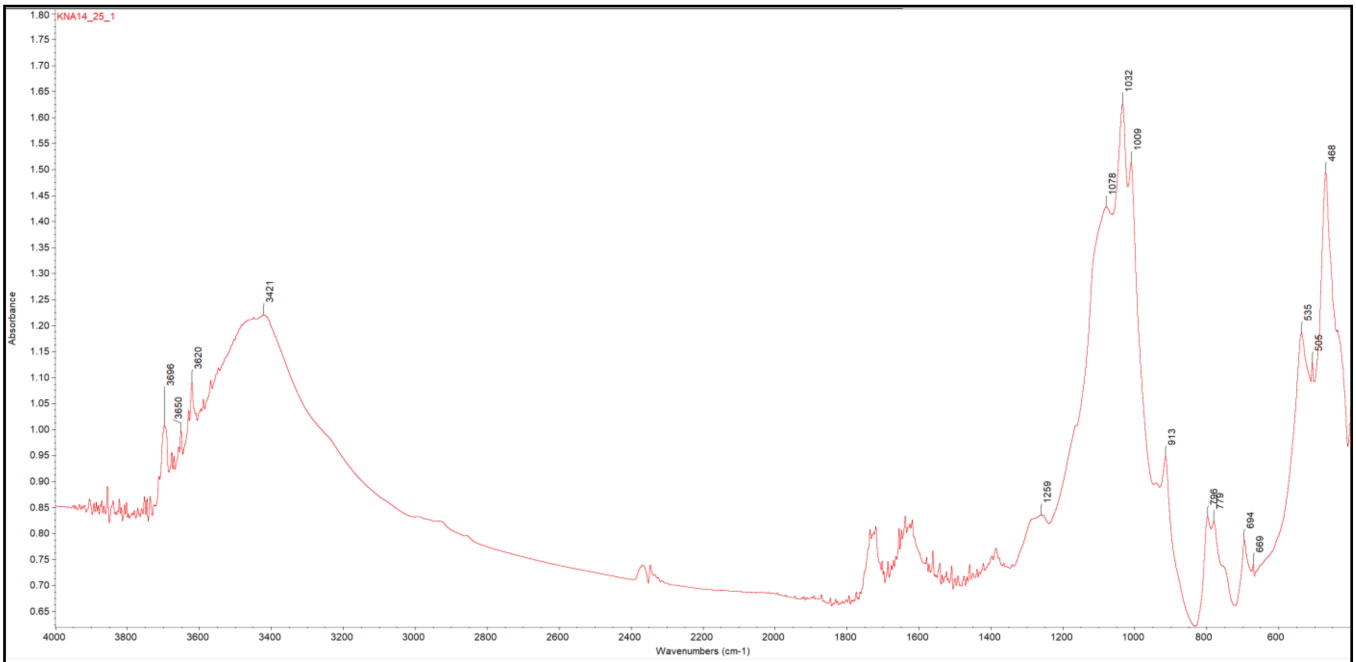

FTIR spectrum

Sample	*Area*	*Site*	*Context*	*Type*	*Date*
KNA14_26	Boko-Songho	Kindangakanzi	SVI 0-10	Slag	15th-17th c.

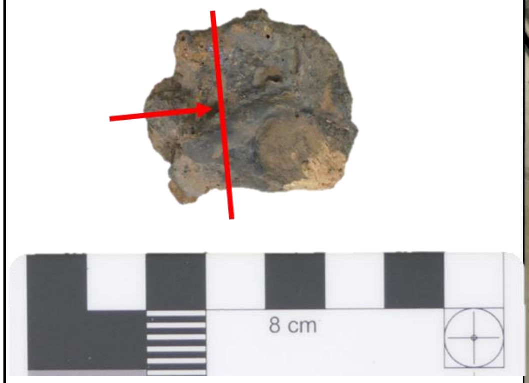

Sample location

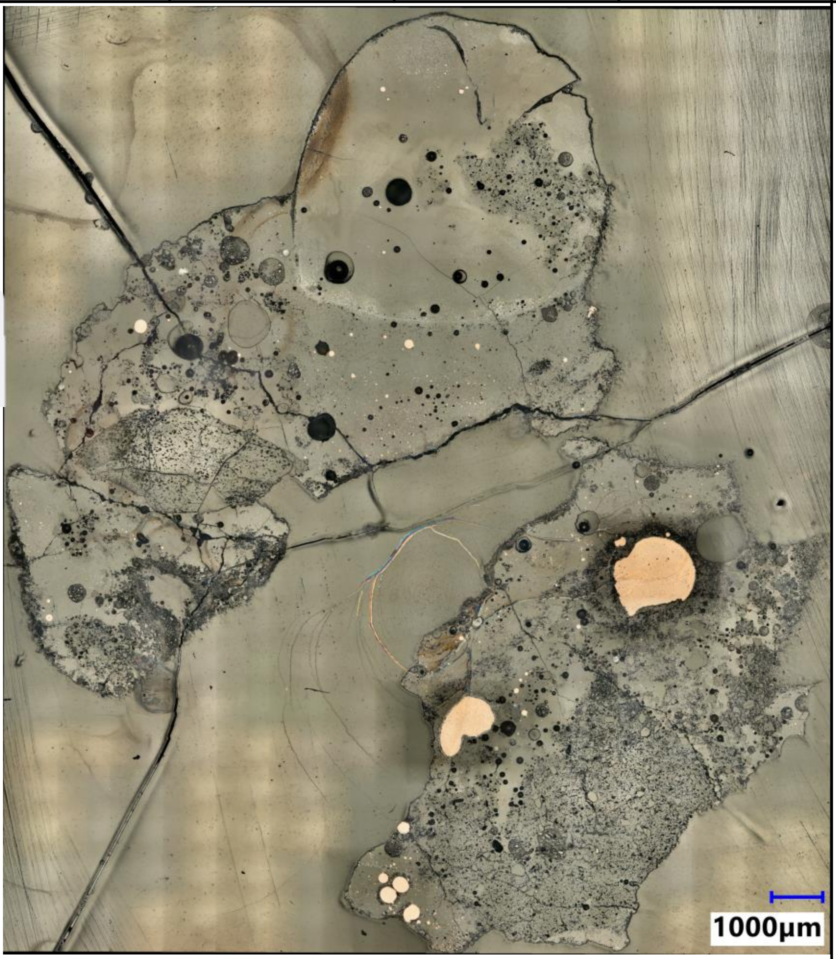

Cross-section

Sample	*Area*	*Site*	*Context*	*Type*	*Date*
KNA14_10	Boko-Songho	Kindangakanzi	SVI 0-10 cm	Tuyère	15th-17th c.

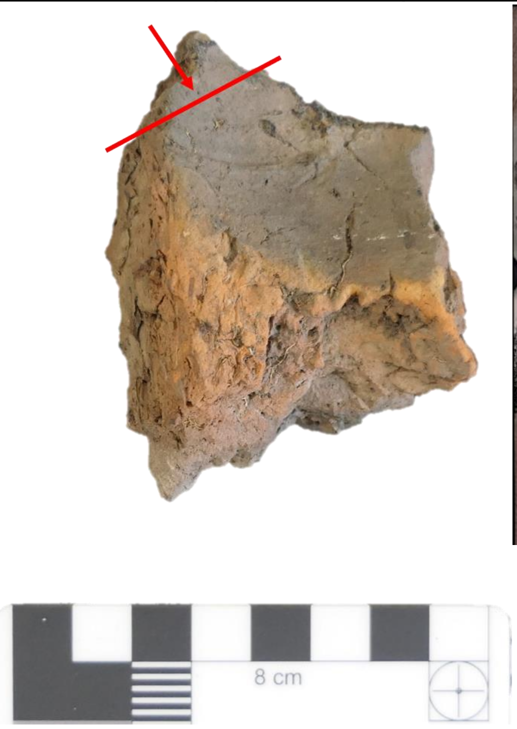

Sample location

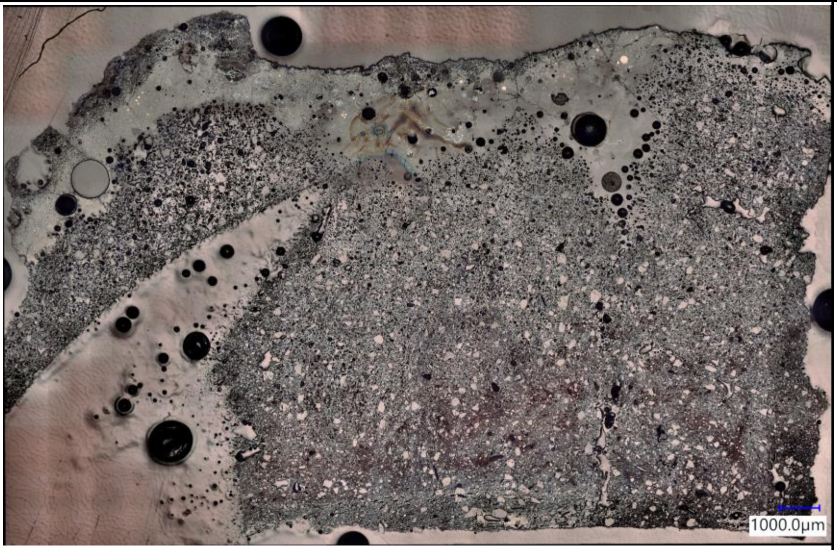

Cross-section

Sample	*Area*	*Site*	*Context*	*Type*	*Date*
MKU3b15_5	Mindouli	Kingoyi	SIII 20-30 cm	Crucible	15th-17th c.

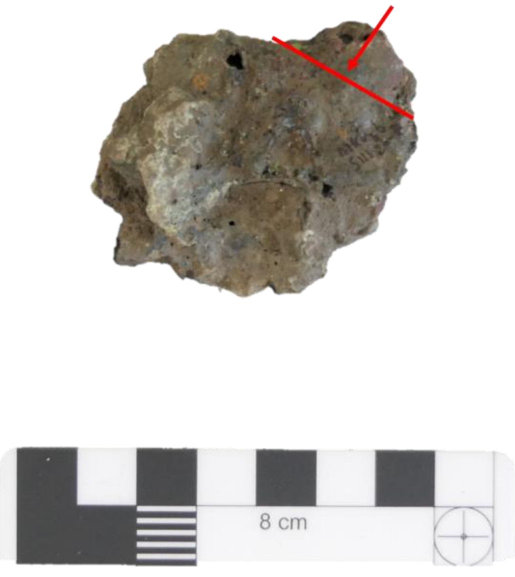

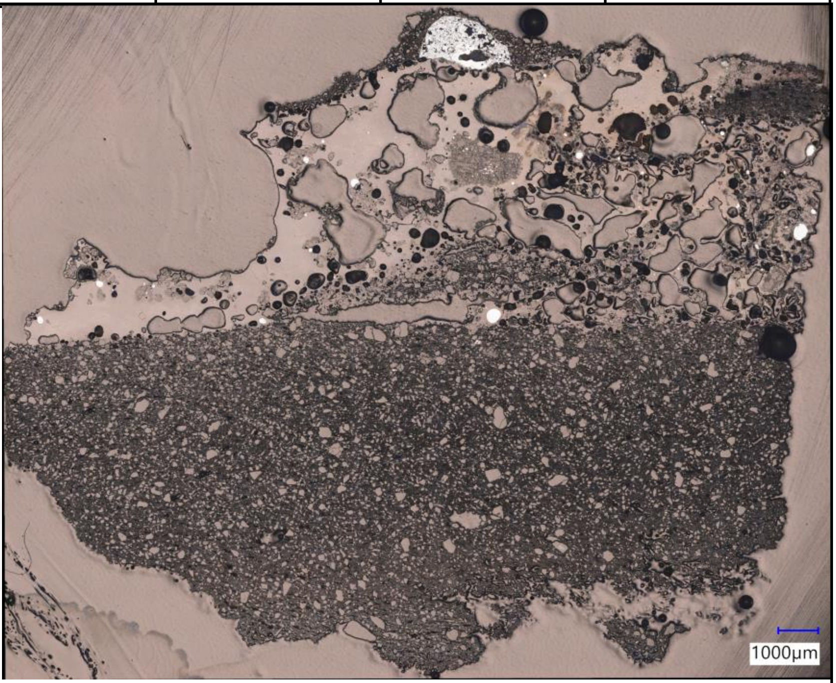

Sample location

Cross-section

Sample	*Area*	*Site*	*Context*	*Type*	*Date*
MKU3b15_13	Mindouli	Kingoyi	SIII 20-30 cm	Crucible	15th-17th c.

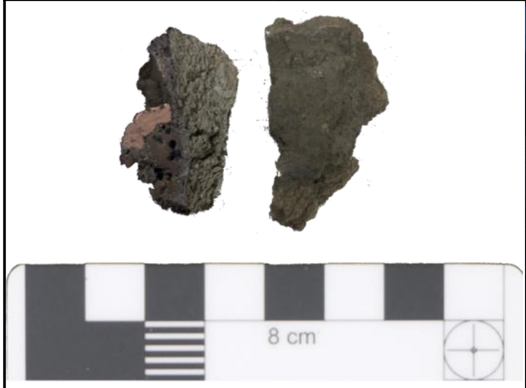

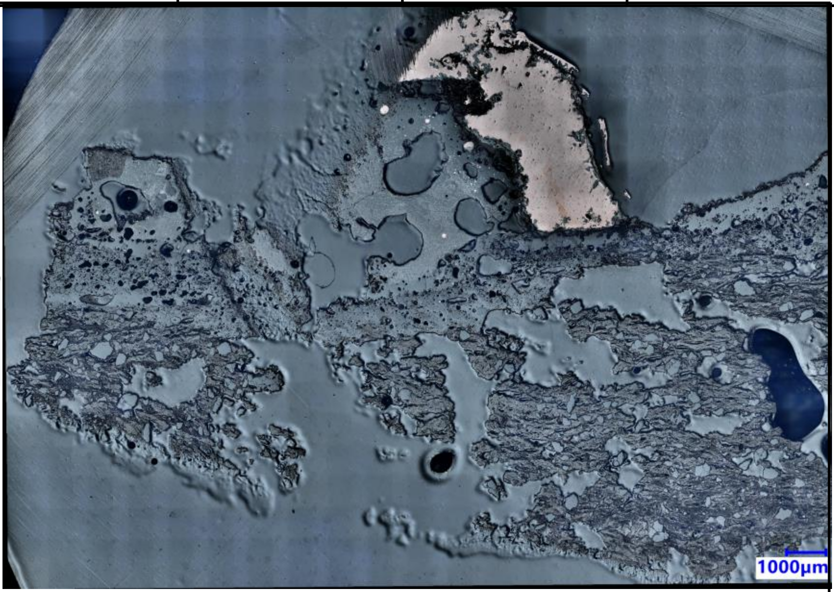

Sample location

Cross-section

FTIR spectrum (ceramic)

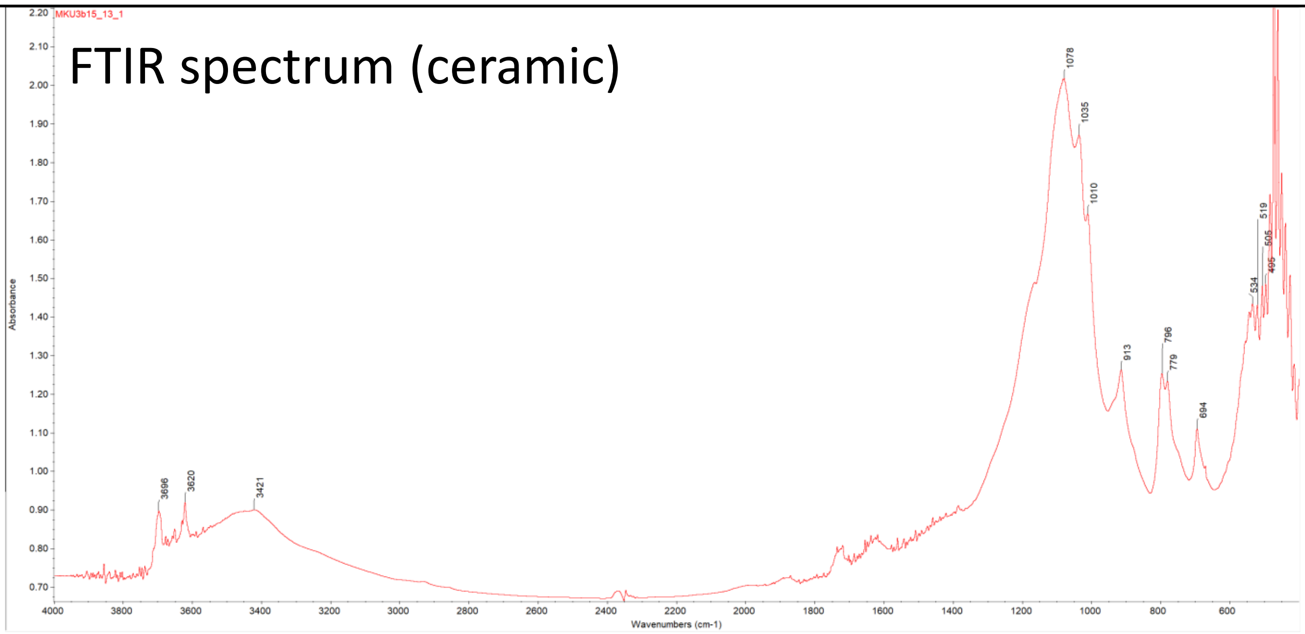

FTIR spectrum (slag)

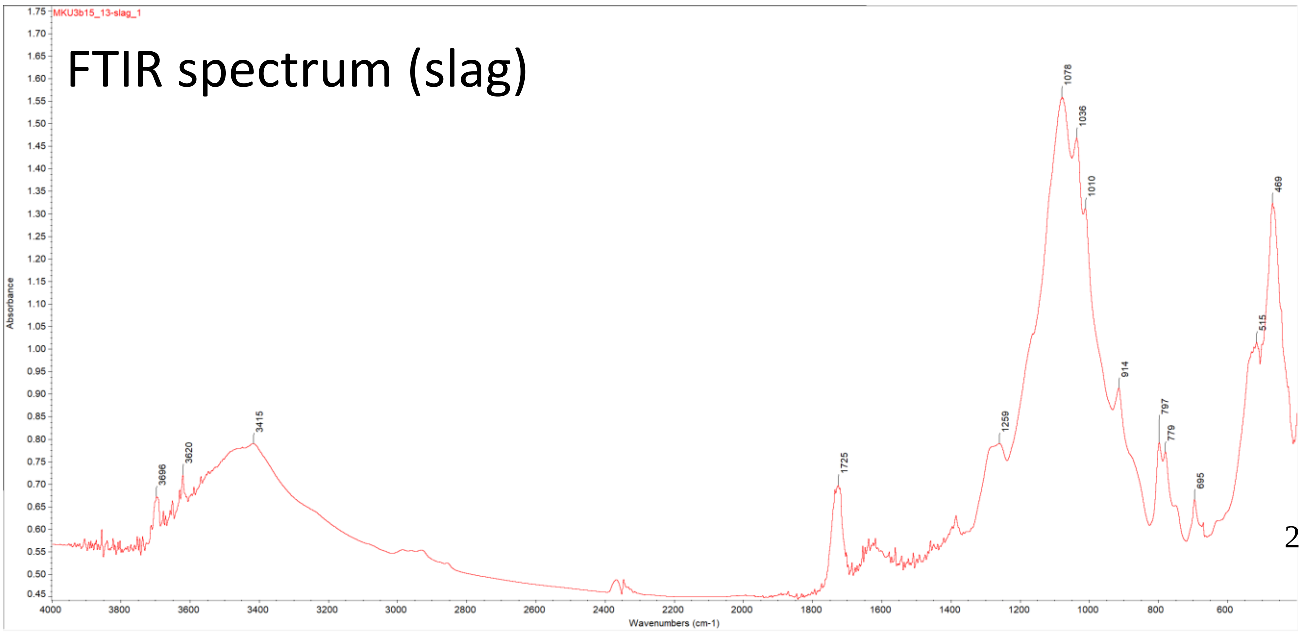

Sample	*Area*	*Site*	*Context*	*Type*	*Date*
MKU3b14_3	Mindouli	Kingoyi	SVIII 20-30 cm	Crucible	15th-17th c.

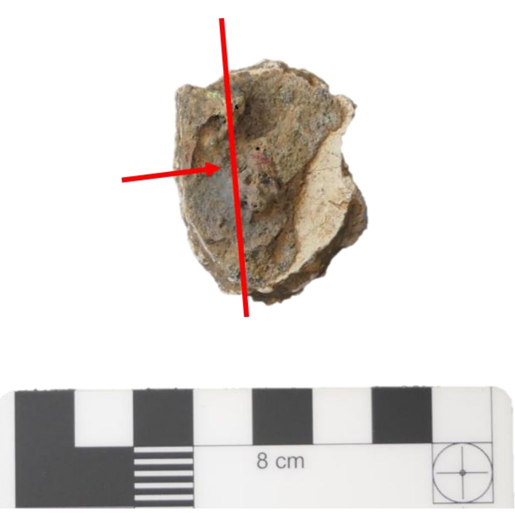

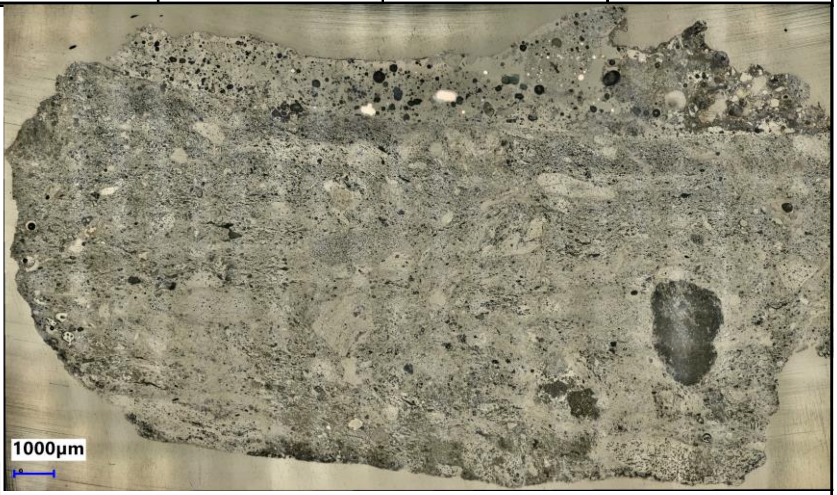

Sample location Cross-section

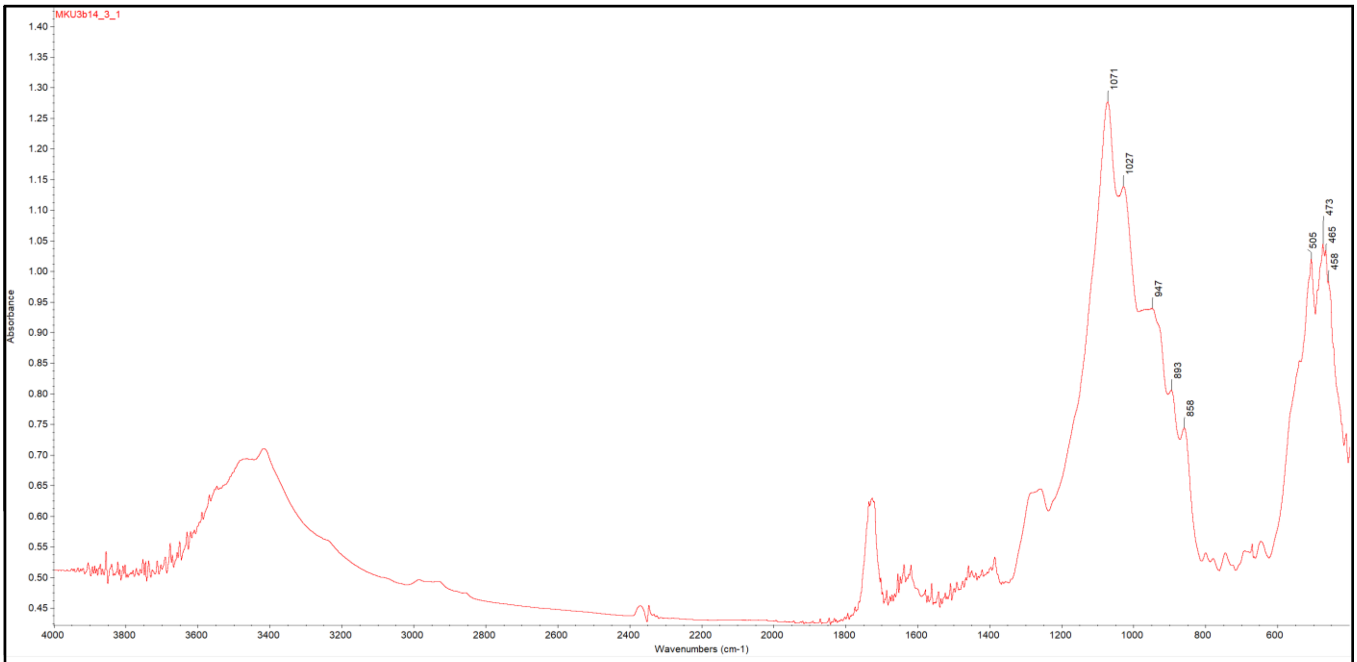

FTIR spectrum

Sample	*Area*	*Site*	*Context*	*Type*	*Date*
MKU3b14_9	Mindouli	Kingoyi	SVIII 10-20 cm	Ore	15th-17th c.

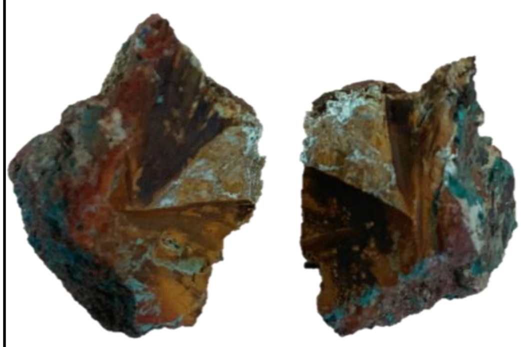

Sample location

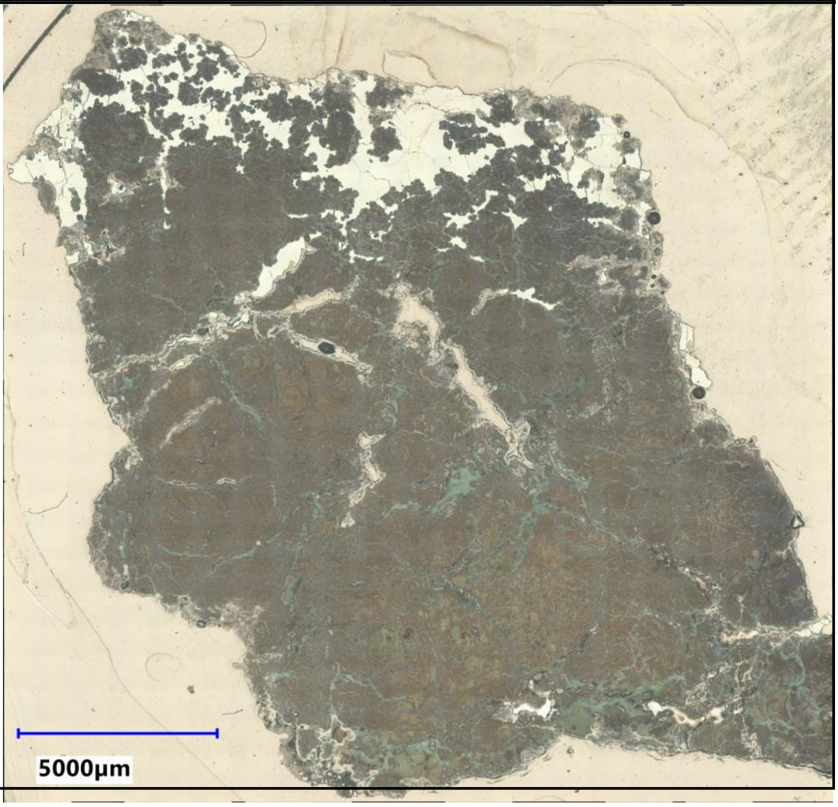

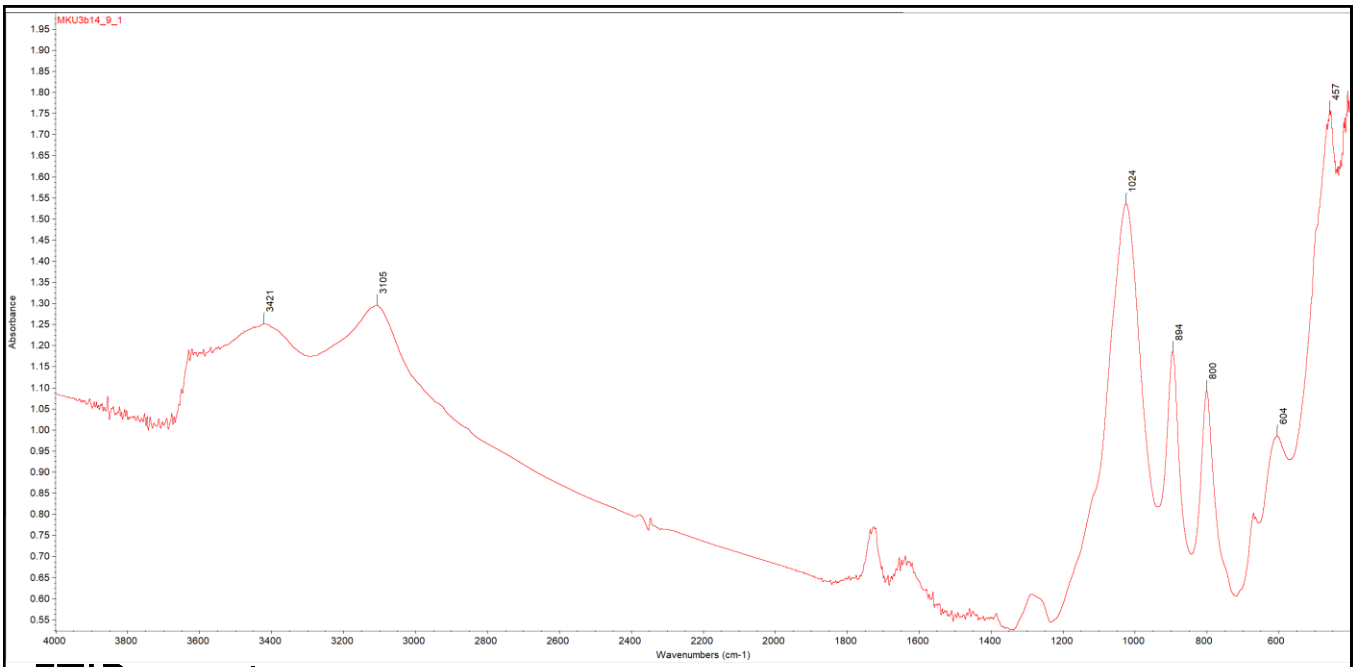

FTIR spectrum (goethite)

Sample	*Area*	*Site*	*Context*	*Type*	*Date*
MKU3b15_3	Mindouli	Kingoyi	SI 0-10 cm	Pottery	15th-17th c.

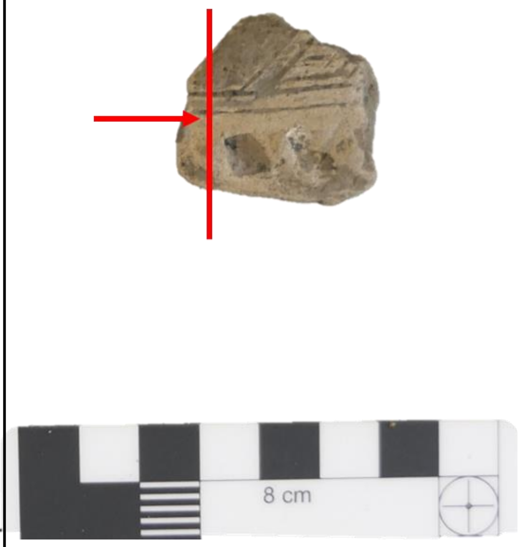

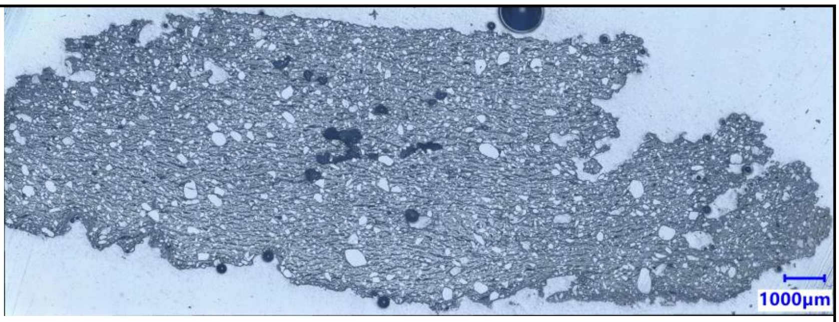

Sample location Cross-section

Sample	*Area*	*Site*	*Context*	*Type*	*Date*
MKU3b15_4	Mindouli	Kingoyi	SIII 10-20 cm	Pottery	15th-17th c.

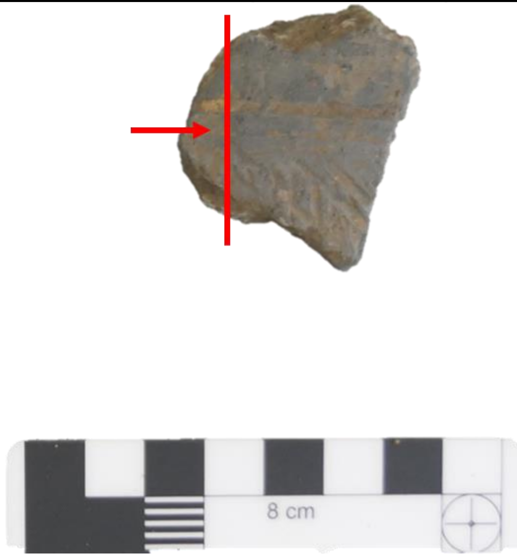

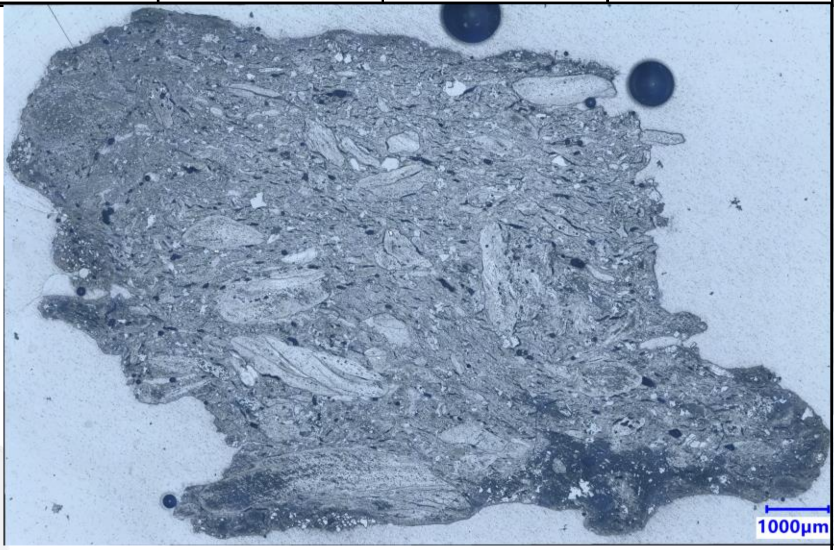

Sample location Cross-section

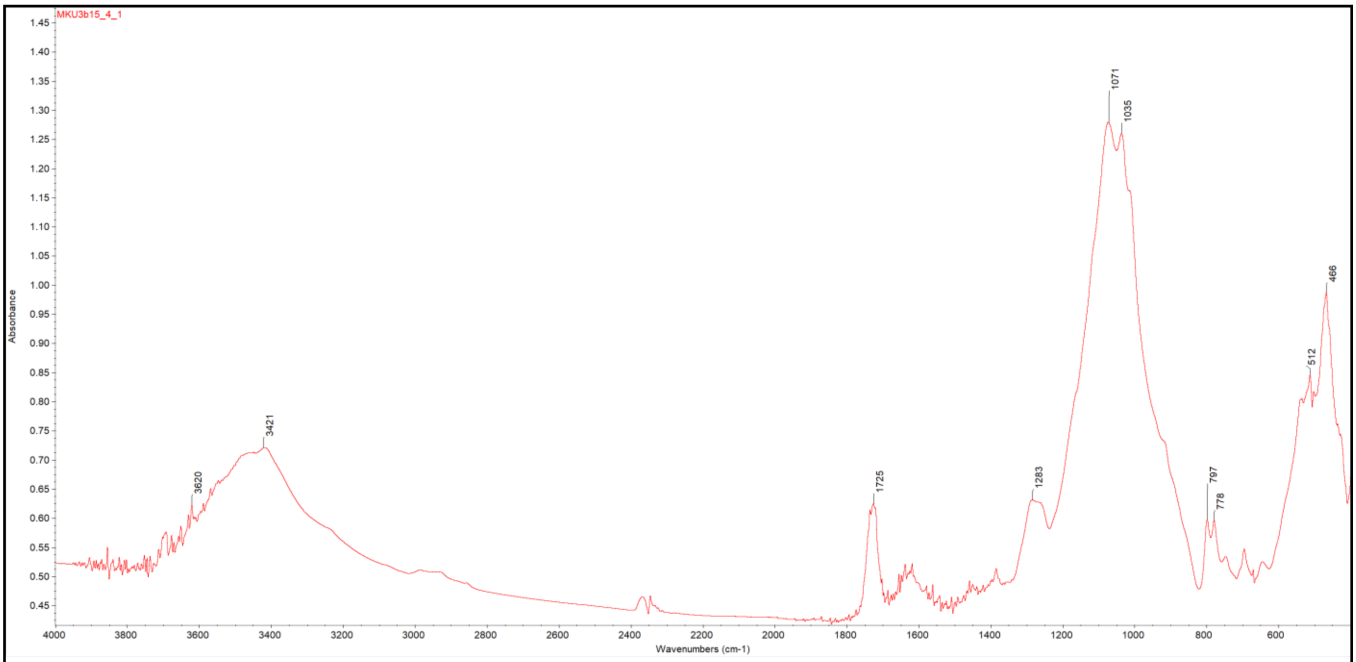

FTIR spectrum

Sample	*Area*	*Site*	*Context*	*Type*	*Date*
MKU3b14_5	Mindouli	Kingoyi	SIII 20-30 cm	Slag	15th-17th c.

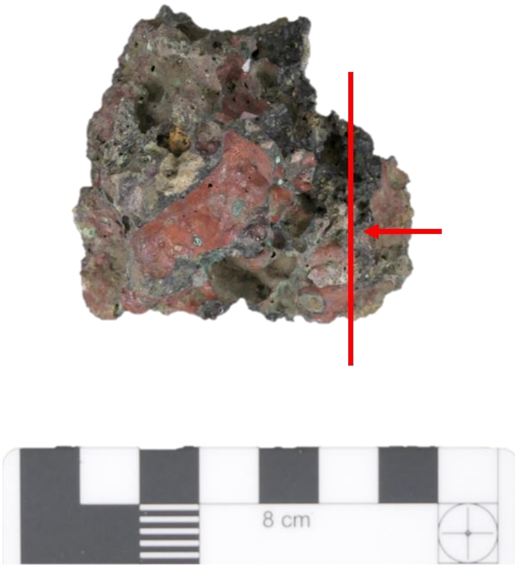

Sample location

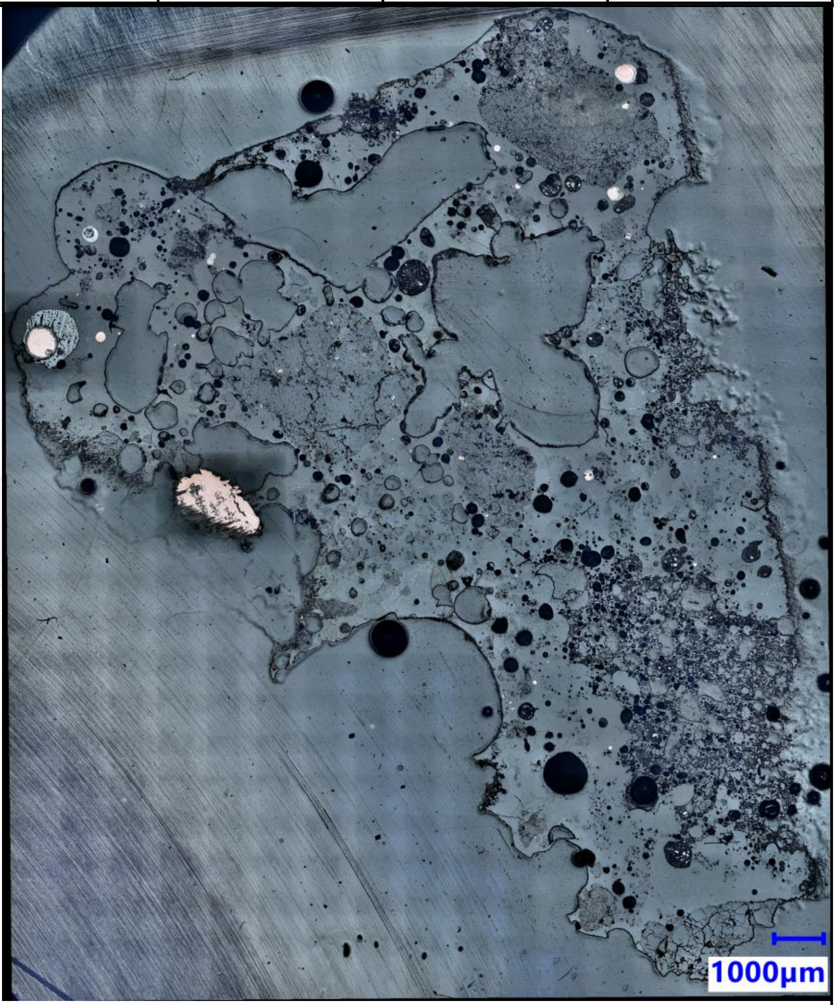

Cross-section

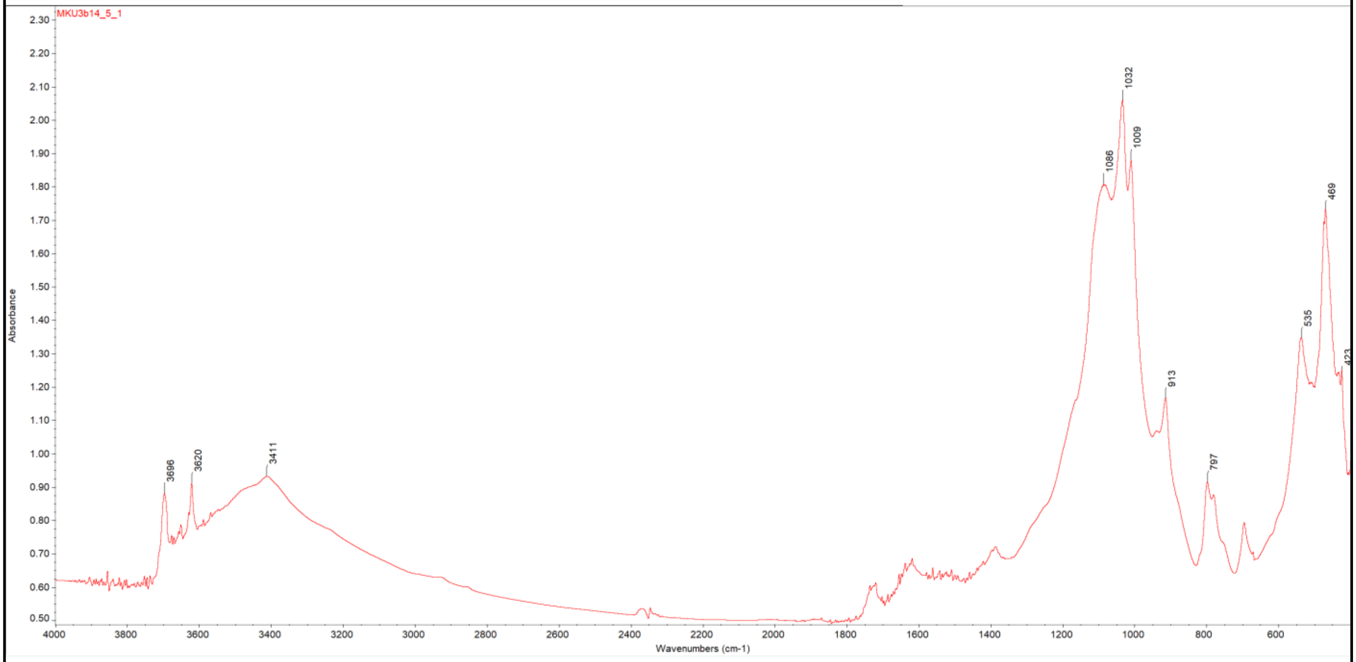

FTIR spectrum

Sample	*Area*	*Site*	*Context*	*Type*	*Date*
MKU3b14_8	Mindouli	Kingoyi	SVIII 10-20 cm	Slag	15th-17th c.

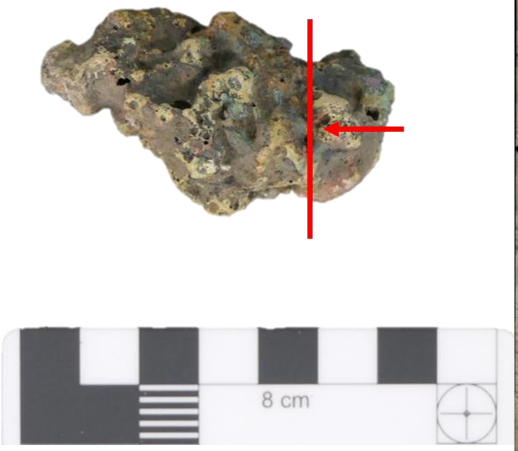

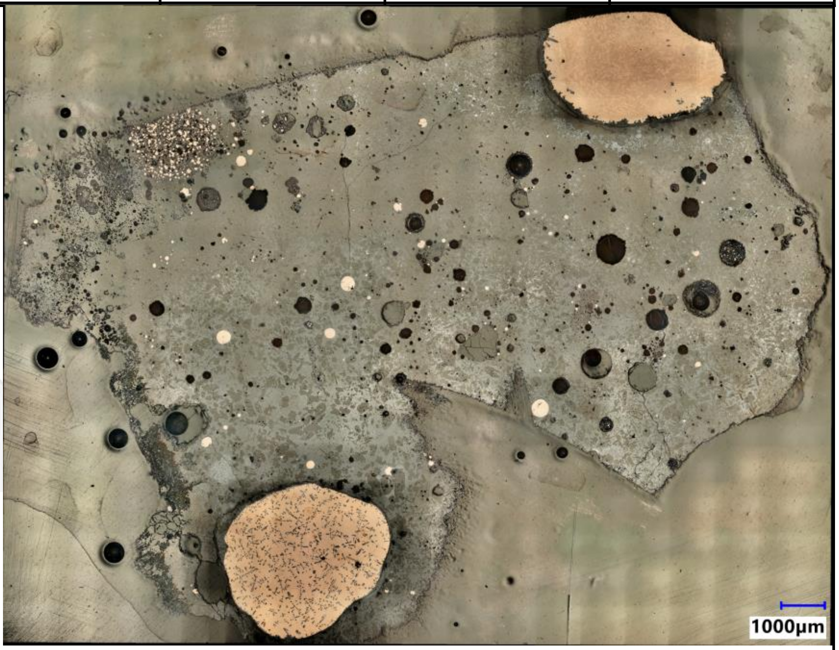

Sample location Cross-section

Sample	*Area*	*Site*	*Context*	*Type*	*Date*
MKU3b15_2	Mindouli	Kingoyi	SI-III 10-20 cm	Tuyère	15th-17th c.

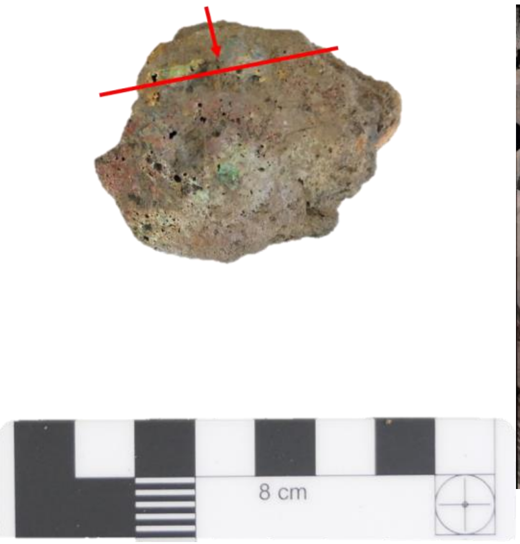

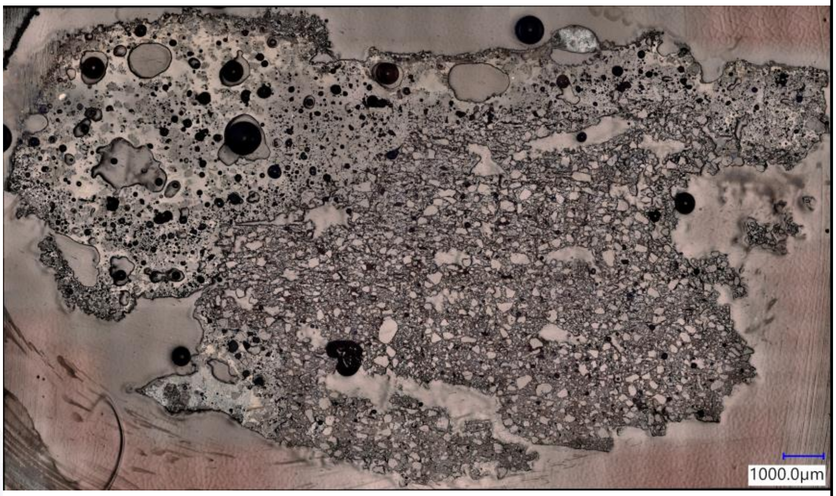

Sample location Cross-section

Sample	*Area*	*Site*	*Context*	*Type*	*Date*
MKU3b14_9	Mindouli	Kingoyi	SVIII 10-20 cm	Ore	15th-17th c.

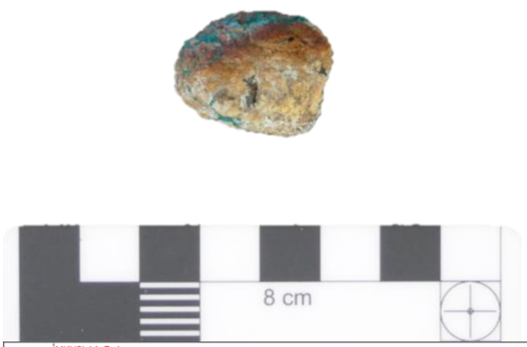

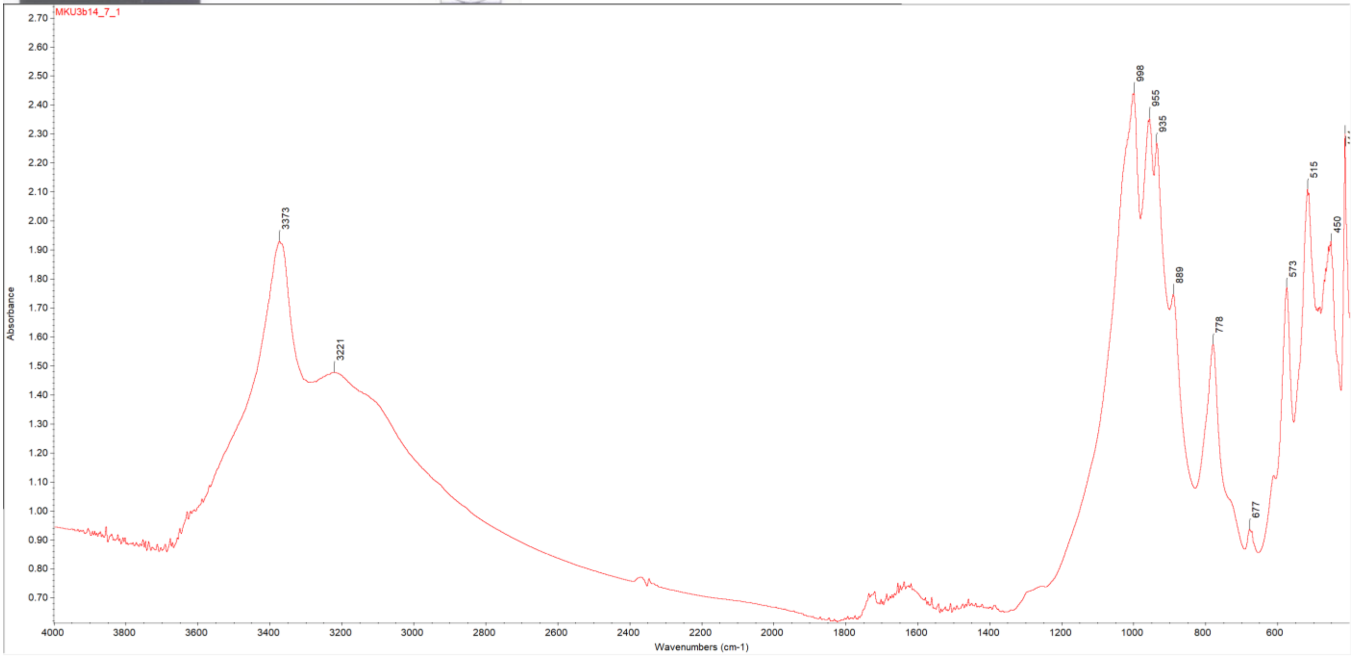

FTIR spectrum (dioptase)

Sample	*Area*	*Site*	*Context*	*Type*	*Date*
GPSNN272_5	Boko-Songho	Malembe	Surface	Ore	

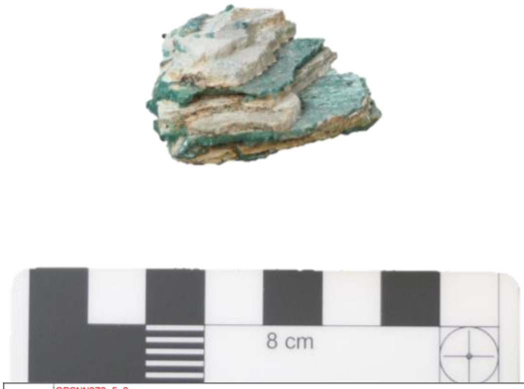

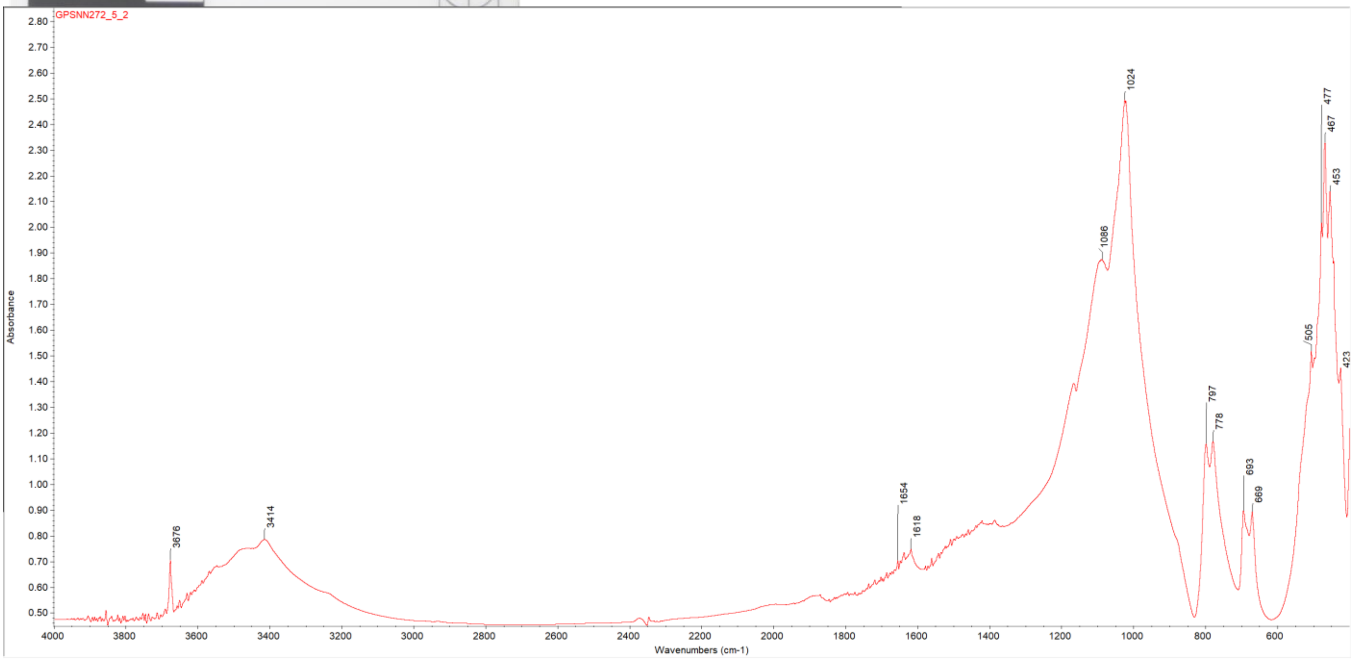

FTIR spectrum (talc)

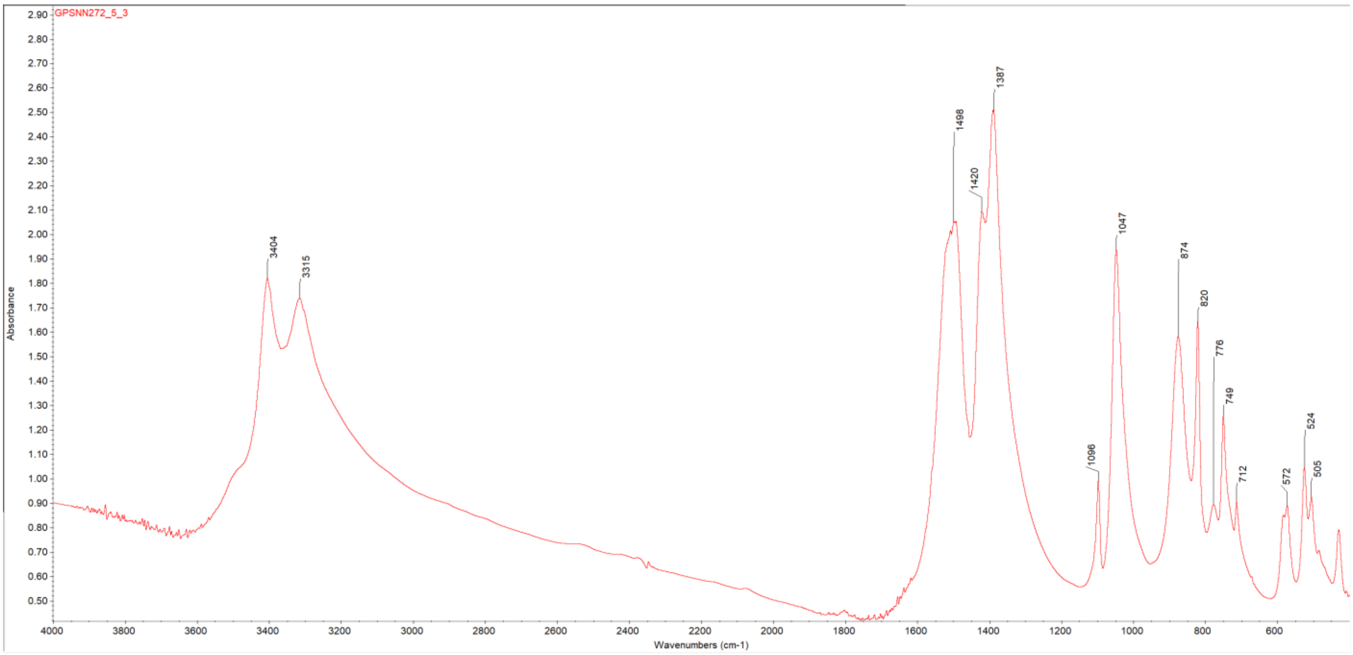

FTIR spectrum (malachite)

Sample	*Area*	*Site*	*Context*	*Type*	*Date*
GPSNN272_9	Boko-Songho	Malembe	Surface	Rock	

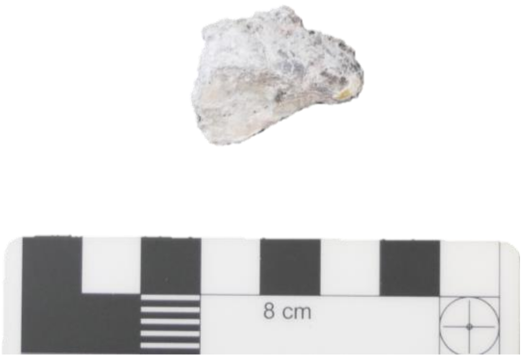

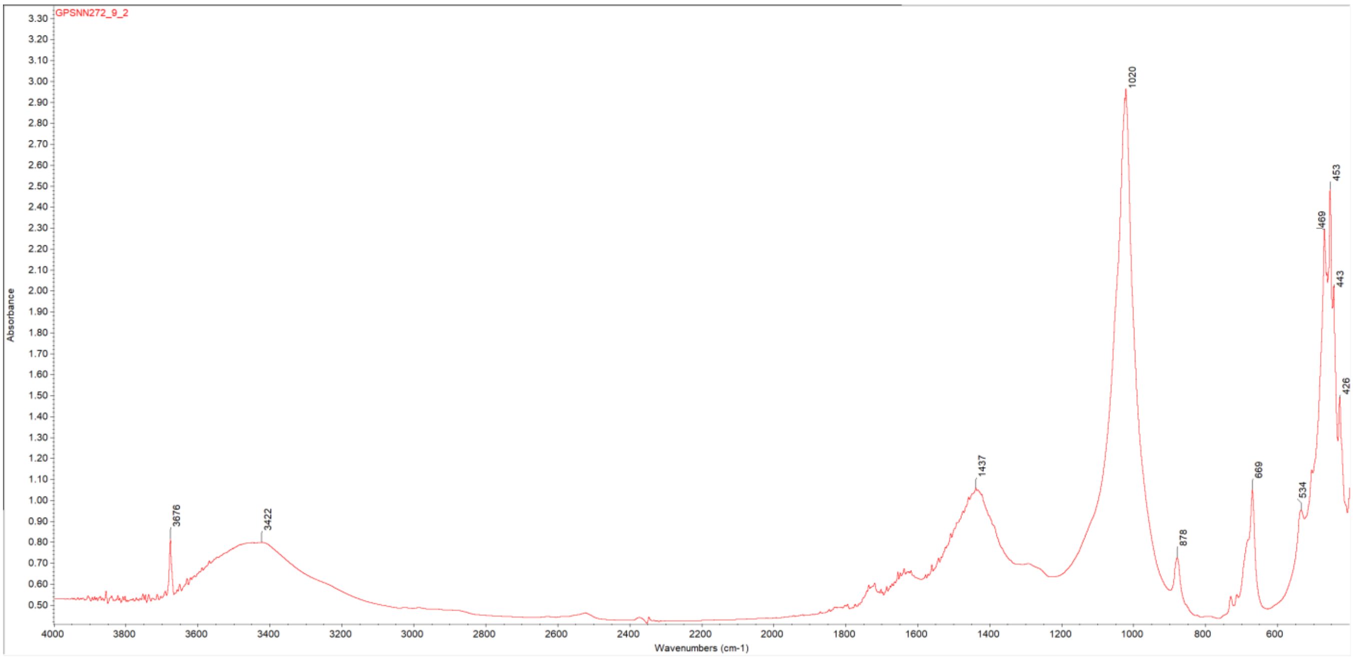

FTIR spectrum (talc)

Sample	*Area*	*Site*	*Context*	*Type*	*Date*
GPSNN113_2	Boko-Songho	Montagne Koka	Surface	Pottery	13th-14th c.

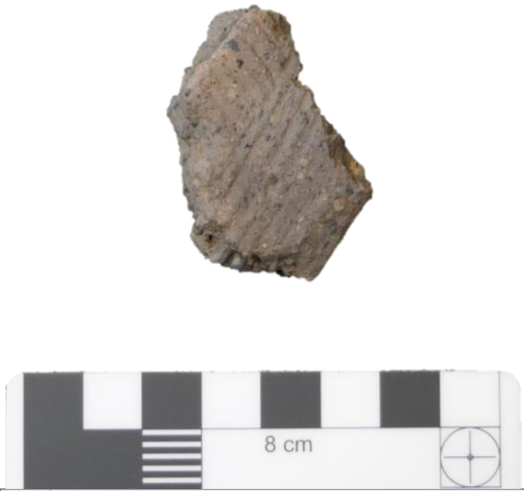

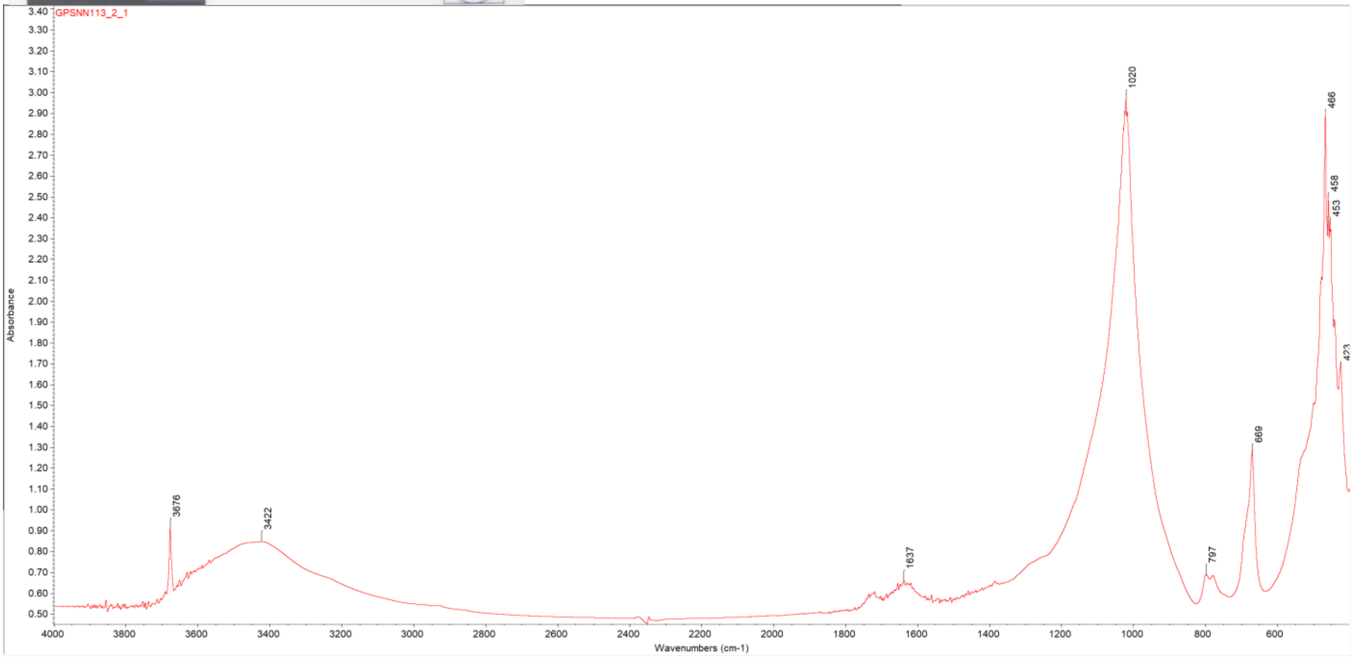

FTIR spectrum

Sample	*Area*	*Site*	*Context*	*Type*	*Date*
MKU3-14_5	Mindouli	Makuti 3	SIV 0-15 cm	Pottery	13th-15th c.

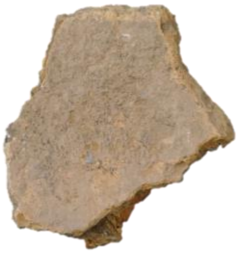

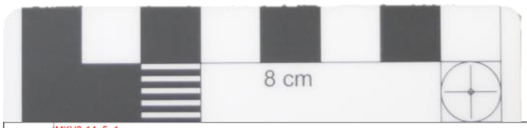

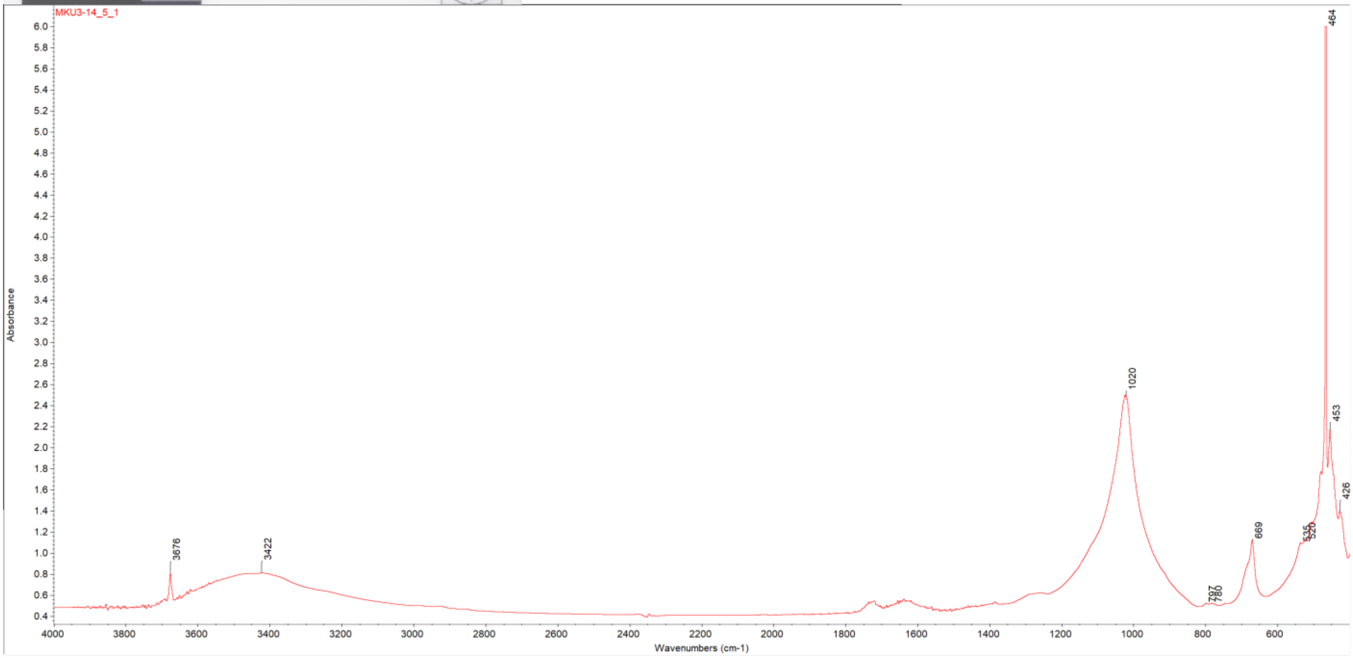

FTIR spectrum
