# Supplementary material for: Smelting copper in decorated pottery: communities of practice in the Niari Basin, Republic of the Congo, fifteenth–seventeenth centuries CE
Source: Archaeol Anthropol Sci. 2022 Oct 6;14(11):210. doi: 10.1007/s12520-022-01653-9 (PMC9537208; doi:10.1007/s12520-022-01653-9)
Supplement: Supplementary file 2 — Supplementary file2 (PDF 74 KB) [file 12520_2022_1653_MOESM2_ESM.pdf]

Smelting copper in decorated pottery: communities of practice in the Niari Basin, Republic of the Congo, 15th-17th centuries CE

Braden W. Cordivari (bwc2354@nyu.edu): University of Cambridge, United Kingdom*
https://orcid.org/0000-0002-8411-4894

Nicolas Nikis: Centre d'Anthropologie Culturelle, Université libre de Bruxelles; Heritage Studies Unit, Royal Museum for Central Africa https://orcid.org/0000-0002-1763-809X

Marcos Martinón-Torres: University of Cambridge, United Kingdom https://orcid.org/0000-0003-2124-2837

*current address: Institute for the Study of the Ancient World, New York University, USA

Abstract: This paper considers copper production in the Niari Basin, Republic of the Congo, during a period dated to the mid-15th-mid-17th centuries CE. Using a combination of pXRF, OM, SEM-EDS, and FTIR, it assesses the microstructure and composition of slags and technical ceramics from sites associated with two different regional pottery traditions: Moubiri-type at the site of Kingoyi near Mindouli and Kindangakanzi-type at Kindangakanzi near Boko-Songho. Both sites are characterised by crucible smelting and the reuse of refractory domestic pottery as crucibles. Moubiri-type pottery is alumina-rich, while Kindangakanzi-type pottery is formed from a magnesia-rich clay, a crucible type unique in sub-Saharan Africa. Similarities in chaînes opératoires at Kingoyi and Kindangakanzi suggest sharing of knowledge at mining and smelting sites, interactions we reconstruct as a metallurgical constellation of practice comprised of the distinct potting communities of practice.

Bokuse : Bazali kolobela koluka mpe kotimola mabulu mpona koluka ba cuivre na etando monene ya Niari, na ekolo mboka Congo, na ekolo oyo ebandaki tona ekeke nzomi na mitano kino na ekeke nzomi na sambo (15ième et 17ième siècle) na ntango na biso. Elobeli mingi mingi microstructure na bosangani ya scories na ba mbeki. Ewuti na bisika to ba mboka mibale oyo eza na lolenge mibale ya mikano mpe evandeli ya banto yakosala ba mbeki : lolenge ya Moubiri na mboka ya Kingoyi pembeni ya Mindouli na lolenge ya Kindangakanzi na Kindangakanzi pembeni ya Boko-Songho. Na kosangisa spectrométrie de fluorescence ya Rayon X portable (pXRF), microscopie optique, microscopie électronique des rayons X na bopeto esangisa spectroscopie yaba rayons X na kolimwa ya bokasi na spectroscopie IRTF. Na bisika yango mibale boluki ezalaki kosalema na kotimola ba mbeki ya misala ya ndako. Ba mbeki ya lolenge moubiri ezali na ebele ya oxyde d'aliminium, kasi oyo ya Kindangakanzi esalema na mabele etondi oxyde de magnesium, esangani na mabulu ya lolenge moko na Afrique subsaharienne. Bokokani ezali mingi na oyo esalemi na Kingoyi mpe na Kindangakanzi ezali kopesa makanisi ya bokaboli mayele na lolenge ya koluka mpe kosalela ba cuivre. Bosangani oyo elakisi bozaleli ya lolenge yako sala mabende esangisi bato basalaka ba mbeki ekeseni.

Résumé : Cet article aborde de la production de cuivre dans le bassin du Niari, en République du Congo, au cours d'une période datée du milieu du 15e au milieu du 17e siècle de notre ère. Il traite plus spécifiquement de la microstructure et de la composition des scories et des céramiques techniques provenant de sites associés à deux différentes traditions régionales de production de poterie : le type Moubiri sur le site de Kingoyi près de Mindouli et le type Kindangakanzi à Kindangakanzi près de Boko-Songho. Les analyses combinent spectrométrie de fluorescence X portable (pXRF), microscopie optique, microscopie

électronique à balayage et spectroscopie à dispersion d’énergie (SEM-EDS), et spectroscopie IRTF. Sur les deux sites, la réduction se fait au moyen de creusets constitués de poteries domestiques réfractaires. La poterie de type Moubiri est riche en oxyde d’aluminium, tandis que la poterie de type Kindangakanzi est formée d'une argile riche en oxyde de magnésium, composition de creuset unique en Afrique subsaharienne. Les similitudes dans les chaînes opératoires à Kingoyi et à Kindangakanzi suggèrent un partage des connaissances sur les sites d'extraction et de réduction de cuivre. Ces interactions reflèteraient l’existence d’une constellation de pratiques métallurgiques regroupant des communautés de pratique de la poterie distinctes.
